# Reconstruction of historical malaria transmission in Senegal using multiplex serocatalytic models

**DOI:** 10.1371/journal.pcbi.1013630

**Published:** 2026-07-27

**Authors:** Gaëlle Baudemont, Thomas Obadia, Laura Garcia, Camille Lambert, Françoise Donnadieu, Fatoumata Diene Sarr, Joseph Faye, Cheikh Sokhna, Inès Vigan-Womas, Aissatou Toure-Balde, Chris Drakeley, Makhtar Niang, Michael T. White

**Affiliations:** 1 Infectious Disease Epidemiology and Analytics Unit, Department of Global Health, Université Paris Cité, Institut Pasteur, Paris, France; 2 Hub de Bioinformatique et Biostatistique, Département Biologie Computationnelle, Institut Pasteur, Paris, France; 3 Pôle Epidemiology, Clinical Research and Data Science, Institut Pasteur de Dakar, Dakar, Sénégal; 4 Institut de Recherche pour le Développement, Laboratoire de Paludologie, Dakar, Sénégal; 5 Pôle Immunophysiopathologie and Maladies Infectieuses, Institut Pasteur de Dakar, Dakar, Sénégal; 6 Department of Infection Biology, London School of Hygiene and Tropical Medicine, London, United Kingdom; 7 Immunophysiopathology and Infectious Diseases Department, Institut Pasteur de Dakar, Dakar, Sénégal; Florida Atlantic University, UNITED STATES OF AMERICA

## Abstract

The advent of multiplexing technologies, allowing antibodies to hundreds of antigens to be measured in a single test, has led to enormous increases in the amount of data generated by serological surveys. New modelling methods are required to exploit this data. This study extends serocatalytic models to consider up to three antibody responses targeting the same pathogen simultaneously. These models were fitted to data from cross-sectional serological surveys of *Plasmodium falciparum* malaria in the Senegalese villages of Dielmo and Ndiop, and model predictions were validated against 22 years of longitudinal epidemiological data. The most accurate reconstruction of historical clinical incidence of *P. falciparum* was provided by a combination of antibodies to Apical Membrane Antigen 1 (*Pf*AMA1) and Glutamate-Rich Protein (*Pf*GlurpR2). This model estimated a 76% (95% CrI: 61% - 86%) drop in transmission in 2004 (95% CrI: 2001 – 2008) coinciding with changing anti-malarial treatment. Multiplex serocatalytic models provided more accurate estimates of past clinical incidence than singleplex serocatalytic models, with the most accurate multiplex model (*Pf*AMA1 + *Pf*GlurpR2) outperforming all singleplex models (KruskalWallis p < 0.01). Finally, models with three antigens did not provide more accurate estimation than models with two antigens.

## Author summary

Multiplex serological assays are accelerating the uptake of serology as a surveillance tool for malaria. However, there has been a lag in the development of analytic tools for the analysis of this rich multiplex data on measurements of antibodies to multiple malaria antigens. In this study, new mathematical models are developed to account for multiplex data. We demonstrate that these models provide more accurate estimates of past malaria transmission in Senegal, with lower levels of uncertainty compared to models based on data from antibodies to a single antigen. By providing more reliable estimation, we anticipate that our work can form the basis of an important new tool for malaria surveillance.

## Introduction

The WHO warns that the malaria burden is increasing compared to the pre-COVID pandemic period [1], with more than 600,000 deaths reported, the majority of which occur in sub-Saharan African countries [1]. 90% of deaths due to malaria are caused by *Plasmodium falciparum* [2], with young children and pregnant women being most affected [3,4]. Overall, 40% of the world’s population live in malaria endemic countries [5]. While this increase in malaria cases comes partly from the interruption of public health systems during the pandemic, it can also be linked to global warming, notably extreme weather events (flooding, heatwaves), the emergence of drug resistance against artemisinin-based combination therapies [6], and a more challenging environment for funding of global health interventions. This emphasizes the necessity of sustained research efforts toward malaria control and elimination and the development of improved surveillance tools to better inform intervention strategies and policy decisions.

Serological surveillance is an important tool to assess malaria transmission [7,8]: it can be implemented in low resource areas and can be more reliable than Entomological Inoculation Rate (EIR) in low-endemic and pre-elimination settings [9,10]. Serological assays detect antibodies induced by both recent and past infections [11], as well as asymptomatic infections, allowing for a detailed overview of population-level exposure. Technologies such as multiplex bead-based assays allow for simultaneous quantification of hundreds or thousands of antibody responses from a few microliters of plasma [12, 13]. Multiplex serological assays implemented on Luminex platforms have a comparable broad dynamic range and robust reproducibility as traditional Enzyme Linked Immunosorbent Assays (ELISA) [14]. In the case of *P. falciparum*, many antibodies have been described from the entire life cycle of the parasite and validated for serological surveillance [7,15,16]. Differences in sensitivity, specificity, longevity, and biological relevance allow antibodies to be used for different objectives, from diagnostic development to assessment of vaccine candidates [17].

Serocatalytic models have been demonstrated to efficiently reconstruct historical exposure to infectious diseases by fitting to age-stratified seroprevalence [18,19]. They are especially useful in places where clinical surveillance is sparse or asymptomatic infections prevail. Data requirements are minimal, typically just age and seropositivity status. Sample sizes are dependent on transmission intensity, but typically a few hundred samples are sufficient [20]. However, the process of dichotomization of continuous antibody measurements can be challenging as there is no one-size-fits-all method for selecting a cutoff for seropositivity [21]. Serocatalytic models account for long term trends of cumulative exposure and tend to smooth out seasonal effects. While the most basic form of the model considers a constant transmission setting, it can be modified to capture changing transmission and waning immunity. Although there have been many important methodological advances for serocatalytic models, analytic tools for interpreting multiplex data are notably lacking. This study extends serocatalytic models to multiplex data and demonstrates their potential for improved serological surveillance. Multiplex serocatalytic models were applied to two Senegalese villages, Dielmo and Ndiop, in which extensive surveillance of malaria has been conducted over the past 30 years. Information on both cross-sectional serological surveys and longitudinal recording of all cases of fever due to *P. falciparum* infection were used to fit and validate these models.

## Results

### Antibody responses to malaria

We measured antibody responses in a total of 1,304 samples. In Dielmo 311 participants were included in 2016 and 208 in 2018. Ages ranged from 5 to 94 years old and are representative of the Senegalese age pyramid. Notably we did not have samples from children under 5 years. In Ndiop, 385 participants were included in 2016 and 316 in 2018. Females were slightly overrepresented in this group with 38% of participants being males. Participants were aged from 4 months old to 82 years old with a median age of 17 years old, and 11% being children under 5 years old. From the SeroPed cohort of French negative controls, 84 randomly selected samples were included. Only adults were part of this sub sample with a median age of 53 [21,90] years old. Information on participants is summarised in Table 1.

**Table 1:** Participant characteristics.

	Dielmo		Ndiop		France
	2016 May-June N = 311 n(%)	2018 December N = 208 n(%)	2016 May-June N = 385 n(%)	2018 December N = 316 n(%)	2020 N = 84 n(%)
**Age**					
<= 5	0 (0%)	0 (0%)	78 (20%)	0 (0%)	0 (0%)
5-10	51 (16%)	35 (17%)	68 (18%)	59 (19%)	0 (0%)
11-25	129 (41%)	78 (38%)	125 (32%)	139 (44%)	8 (9.5%)
26-50	84 (27%)	52 (25%)	70 (18%)	78 (25%)	31 (37%)
51-75	45 (14%)	40 (19%)	43 (11%)	37 (12%)	27 (32%)
75+	2 (0.6%)	3 (1.4%)	1 (0.3%)	3 (0.9%)	18 (21%)
**Sex**					
Female	161 (52%)	107 (51%)	243 (63%)	192 (61%)	47 (56%)
Male	150 (48%)	101(49%)	142 (37%)	124 (39%)	37 (44%)

Number of samples available for analysis and epidemiological data from Dielmo and Ndiop in two cross-sections and from French samples used as negative controls.

**Fig 1:**
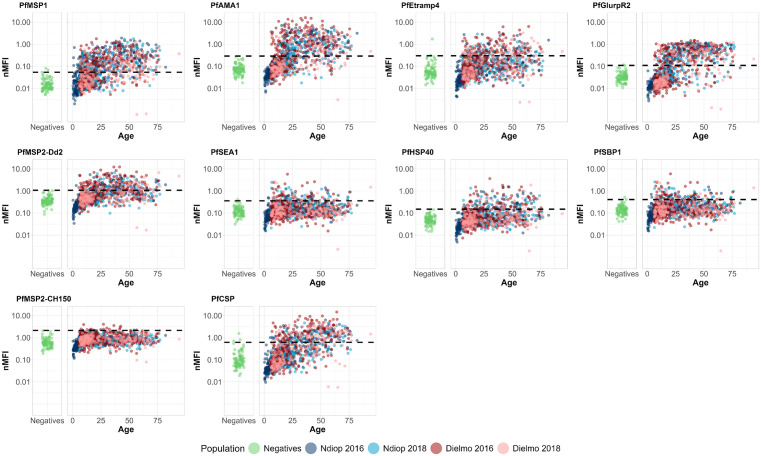
Antibody responses. Age stratified quantification using multiplex technology of ten *P. falciparum* antigens, observed in two villages from two cross-sections. Results are given in normalised Median Fluorescence Intensity (nMFI) and are compared to French negative controls. The dashed black line represents the selected cutoff to distinguish seropositive from seronegative participants. Circumsporozoite protein (*Pf*CSP), Apical Membrane Antigen 1 (*Pf*AMA1), Merozoite Surface Protein 1 (*Pf*MSP1), Glutamate Rich Protein C-terminal repetitive segment (*Pf*GlurpR2), the two major allelic types of Merozoite Surface Protein 2 (*Pf*MSP-2.Dd2 and *Pf*MSP-2.CH150), Sporozoite Binding Protein 1 (*Pf*SBP1) and Sporozoite Exported Antigen 1 (*Pf*SEA1), Heat Shock Protein 40 (*Pf*HSP40) and Early Transcribed Membrane Protein 4 (*Pf*Etramp4).

A pattern of increased antibody response with age is observed for all tested antigens which is typical of endemic settings with continuous exposure to malaria (Fig 1). The distribution of the antibody responses for Dielmo and Ndiop in both 2016 and 2018 overlaps considerably. For antibodies to all the studied antigens, seroprevalence was higher in Dielmo than in Ndiop, and higher in 2016 than in 2018, with the majority of differences being statistically significant (Table A and B in S1 Appendix). Negative control French samples had antibody responses in the lower range of the distribution. The lowest antibody responses were observed in children under five years of age from Ndiop.

### Seropositivity classification

**Fig 2:**
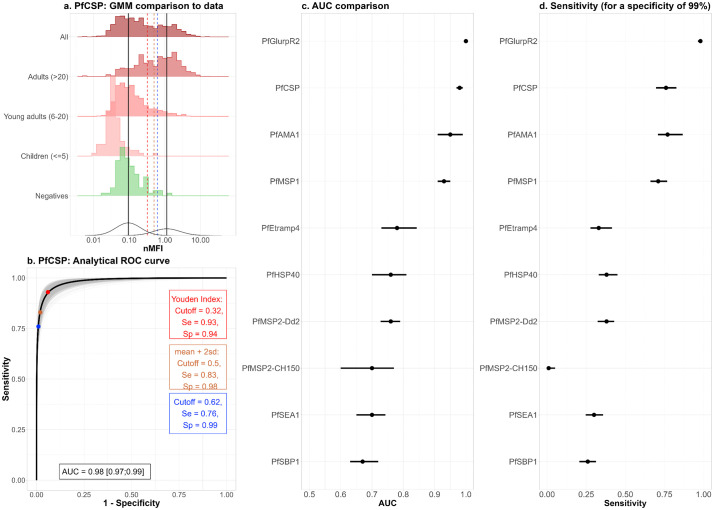
Antigen performance for seropositivity determination. Panel A shows the distribution of PfCSP antibody responses compared to the bimodal distribution estimated by the GMM. Black vertical lines are the estimated means of the components representing the negative and positive samples. Dashed coloured lines are the selected cutoffs. Panel B shows the analytical ROC curve from the GMM fitted to PfCSP as well as the different cutoffs considered and their associated sensitivity and specificity. Panel C presents a ranking of antigens based on their performance to distinguish negative and positive samples as measured by AUC. Panel D shows the sensitivity associated with a cutoff corresponding to a specificity of 99%. Estimates are shown as posterior medians with 95% credible intervals.

Gaussian Mixture Models (GMMs) applied to data from Senegalese and French samples were capable of recapturing the observed distribution of antibody responses as a combination of two components: shown in Fig 2B for *Pf*CSP (Circumsporozoite protein), and in the supplementary information for the other antigens. The French negative control samples fall in the lower component, while for the Senegalese samples the proportion of samples falling in the upper component increases with age. For three antibody responses, *Pf*MSP-2.CH150 (Merozoite Surface Protein 2), Sporozoite Binding Protein 1 (SBP1) and Sporozoite Exported Antigen 1 (SEA1), the degree of overlap of the positive and negative distributions is too great to allow for informative classification of the samples (AUCs ≤ 0.7 Figs 2C and *Fig A-C in* S1 Appendix). These antigens are not well suited to discriminate between participants that have been exposed or not to *P. falciparum*. Four antigens *Pf*GlurpR2 (Glutamate Rich Protein C-terminal repetitive segment), *Pf*CSP, *Pf*AMA1 (Apical Membrane Antigen 1), and *Pf*MSP1 (Merozoite Surface Protein 1), have bimodal distributions and are able to classify samples (AUCs ≥ 0.9 Figs 2 and *Fig G-I in* S1 Appendix).

### Reconstruction of historical malaria transmission

**Fig 3:**
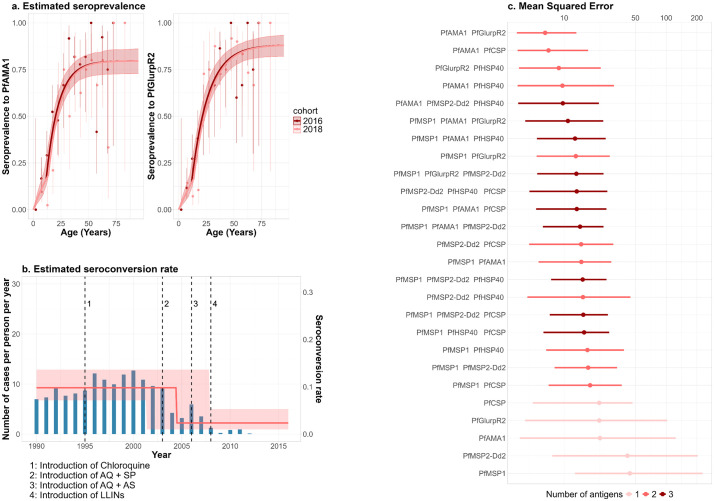
Model comparison to data and validation. Panel A compares Dielmo’s observed and model-predicted age-stratified seroprevalence for a multiplex serocatalytic model including both PfAMA1 and PfGlurpR2 and considering one drop in transmission. In Panel B the seroincidence estimated by the same model over 25 years prior to the 2016 cross-section is compared to external validation data on the observed number of cases per person per year. Panel C ranks every model that converged depending on their capability of recapturing the validation data. Mean Squared Error (MSE) between observation and model estimation and the associated 95%CI were computed after a conversion coefficient was applied to the posterior distribution of the seroincidence λ.

A simple serocatalytic model including one change in transmission was first fitted to each antibody response observed in Ndiop where samples from children under five years old were available. Convergence issues occurred for four antibody responses with low sensitivity (Erythrocyte Binding Antigen 4 (Etramp4.Ag2), *Pf*MSP-2.CH150, SBP1 and SEA1) (Fig 2D) and were ruled out of further analysis. Estimated seroreversion rates were used as informative priors for models fitted to data from Dielmo. Models with up to three antigens were fitted to the Dielmo data. All models that converged were capable of recapturing age-stratified seroprevalence. All models estimated a sharp drop in transmission within the period of the four public health interventions with a decrease in transmission of greater than 50%. The best model, based on its ability to reconstruct the number of cases per person per year, includes both *Pf*AMA1 and *Pf*GlurpR2, two long-lived blood stage antigens [22]. *Pf*AMA1 is known to be very immunogenic and is recommended for serological surveillance in low transmission settings [23,24]. Here, the estimated probability to seroconvert if exposed is 73% (56%, 88%) for *Pf*AMA1 and 60% (48%, 72%) for *Pf*GlurpR2 (Fig 3A). This model estimated a drop in transmission in 2004, 11.6 (8.2, 14.6) years prior to 2016, with a seroincidence rate of 0.10 (0.07, 0.14) year^-1^, before 2004 and a decrease of 76% (61%, 86%) afterwards (Fig 3B). This coincides with the implementation of combination drugs (AQ + SP and AQ+AS) that were introduced systematically in Dielmo in 2003 and 2006. Models estimating a second drop in transmission were tested, but did not converge indicating that the data were not sufficiently informative to identify two changes in transmission.

### Multiplex serocatalytic models provide more accurate estimation of transmission than singleplex models

The performance of models to reconstruct past transmission was validated against longitudinal data on cases of fever due to *P. falciparum* infection and the past implementation of public health interventions. The ranking of models based on mean squared error to this validation data differs statistically depending on the number of antigens included (Kruskal-Wallis p ≤ 0.01). The most accurate multiplex model (*Pf*AMA1 + *Pf*GlurpR2) outperformed all singleplex models. Overall, models including only one antigen provided less accurate estimates of past clinical incidence and show systematically higher MSE than models with multiple antigens. However, there were limited differences in MSE between models with two and three antigens. Not only are the models with one antigen less accurate at recapturing past transmission, but their uncertainty is wider as well (Fig 3C). The same thing is found when looking at the posterior distributions of each parameter: singleplex serocatalytic models give less precise and more variable estimates, than multiplex models (*Fig N-W in* S1 Appendix).

## Discussion

In Dielmo, between 1990 and 2012 public health interventions have been implemented according to Senegalese guidelines for *P. falciparum* case management. Following the development of parasite resistance to chloroquine, combination therapies were introduced, first amodiaquine plus sulfadoxine-pyrimethamine, and then amodiaquine plus artesunate [25]. The impact of these new first line treatments, as well as the introduction of insecticide treated bed nets, can be observed in the reductions in parasite prevalence and case incidence in longitudinal data collected over a 22 year period [25]. We were able to reconstruct this reduction in transmission by fitting serocatalytic models to measured antibody responses from cross-sectional surveys in 2016 and 2018. While serocatalytic models fitted to ten antibody responses independently give similar estimates, synthesizing these outputs into a unique measure of transmission is challenging. The multiplex serocatalytic models presented here allow antibody responses to two to three antigens to be combined, resulting in parameter estimates with lower uncertainty and more reliable reconstruction of validation data.

Not all markers were capable of reconstructing historical *P. falciparum* transmission. Antibody responses to SBP1, SEA1 and *Pf*MSP-2.CH150 did not provide clear discrimination between exposed and unexposed individuals. Conversely, antibodies to *Pf*GlurpR2, PfCSP, *Pf*MSP1 and *Pf*AMA1 had wide dynamic ranges, allowing discrimination between exposed and unexposed populations. *Pf*AMA1 has been shown to be highly immunogenic in many different transmission settings [24,26]. Combining *Pf*AMA1 with *Pf*GlurpR2 resulted in an estimated 76% drop in transmission. However, uncertainty in the estimated time at which transmission drops is too wide to attribute the reduction to a particular intervention.

There were several limitations to our modelling framework. Firstly, the GMM assumes that the French population is seronegative and that the Senegalese population is composed of both seronegative and seropositive individuals. As there have been reported differences in total IgG antibody levels between European and African populations [27], this may introduce some bias to estimated cutoffs. Secondly, dichotomization of continuous antibody titres to determine serostatus involves loss of information. Alternative models have been developed to avoid this assumption allowing serocatalytic models to be fitted directly to continuous antibody titres [28]. Further work could extend such methods to multiplexed serocatalytic models. Thirdly, serocatalytic models do not account for boosting of antibody responses in seropositive individuals. In higher transmission settings, individuals are expected to have higher antibody levels which have been demonstrated to be associated with lower seroconversion rates [29], violating the assumption of constant seroreversion. Therefore, seroreversion rates may not be constant across differing transmission settings.

Although many residents of Dielmo were included in both the 2016 and 2018 cross-sections, repeat samples from the same individuals were treated independently in the likelihood. Development of a modelling framework accounting for longitudinal sampling may lead to improved estimates of seroconversion and seroreversion rates. Seroprevalence has been demonstrated to vary seasonally, but serocatalytic models recapture historical cumulative exposure over many years and are unable to account for short-term transmission trends.

There were limitations to the data used in this study, most notably the absence of samples from children under 5 years from Dielmo. Serological data from young participants are particularly informative in serocatalytic models. The absence of data from these samples prevents reconstruction of changes in malaria transmission in the past five years. This was mitigated by informing priors with the posterior distribution from singleplex models fitted to data from Ndiop which contained samples from people of all ages. The framework would benefit from additional validation of the association between seroconversion rate and historical clinical incidence in different epidemiological settings.

With each antigen added, the number of serostatuses, and so the number of equations in the system increases exponentially while the number of parameters increases linearly. This suggests that although adding more antigens may not cause identifiability issues, the complexity of the models and computation time would become limiting. Furthermore, the information gained from each antigen added is decreasing due to high correlation between antibody responses. For these reasons, a maximum of three antibody responses were included simultaneously in the model. Models estimating multiple changes in transmission are theoretically possible, but a practical limitation is that these models do not tend to be identifiable for more than two changes in transmission.

The model failed to converge for antigens that were of poor quality (e.g., PfMSP2-CH150) or for antigens that do not generate a long-lasting antibody response, resulting in a lack of age-dependent increase (e.g., PfSEA1, PfSBP1, PfHSP40). For 2 antigen models, 11/15 (73%) evaluated models converged, and for 3 antigen models 10/20 (50%) evaluated models converged. The lower proportion of models converging for larger number of antigens may be attributable to correlations between antibody responses to different antigens.

This modelling study contributes to broader efforts to leverage multiplex serological data now available, especially as a new study highlights the relevance of serocatalytic models for decision making [30], and to model antibody responses and the correlation between them. While models taking into account cross reactivity have been developed [28] to our knowledge none have been implemented to look at multiple antibody responses to the same pathogen.

In this study, new models for serological surveillance have been applied to inform malaria transmission in an endemic region in Senegal. Required conditions to implement these models are that each individual antigen has the capacity to discriminate exposed from nonexposed individuals, and that different antigens provide independent information. This methodology could also be used on a wide variety of pathogens (arboviruses, respiratory viruses, enteric viruses) and, like serocatalytic models they can be applied to populations in a broad variety of transmission settings.

## Materials and methods

### Ethics statement

The Senegalese samples were collected as part of the ongoing Dielmo and Ndiop project which was examined and approved by the Senegalese National Health Research Ethics Committee (CNERS Sénégal). Approval to measure antibodies to malaria in these samples was granted by CNERS Sénégal (N 00000007 MSAS/CNERS/Sec). Written informed consent was given by participants and parents or guardian of children enrolled.

The French cohort was approved by the Comité de Protection des Personnes Nord Ouest IV (NCT04644159) and samples were processed in line with the Commission Nationale de l’Informatique et des Libertes reglementation.

### A. Data

#### Serological assay.

The antigens used in this study spanned the whole life cycle of *P. falciparum* in humans. All antigens were generated and expressed by *E. Coli* except for *Pf*AMA1 which was expressed in *Pichia Pastoris*. Antigens were selected based on their association with infections among children [11] or exposed volunteers [31] and their use was validated for malaria surveillance [11,12,31]. Circumsporozoite protein (CSP) is present on sporozoites and is the antigen included in the WHO recommended RTS,S/AS01 and R21/Matrix-M malaria vaccines. Apical Membrane Antigen 1 (AMA1) is expressed on blood-stage merozoites, and to a lesser degree on sporozoites. Merozoite Surface Protein 1 (MSP1), Glutamate Rich Protein C-terminal repetitive segment (GlurpR2), and the two major allelic types of Merozoite Surface Protein 2 (MSP-2.Dd2 and MSP-2.CH150), are targeted at merozoites’ surface. Etramp4.Ag2, HSP40.Ag1, SBP1 and SEA1 are targeted in infected red blood cells. Additional information on these antigens’ characteristics including production system and sequence information is reported in van den Hoogen *et al* [31] and Wu *et al* [12].

The selected antigens were coupled to Luminex microspheres to develop a multiplex bead-based assay [32,33]. Briefly, using a KingFisher Duo Prime magnetic particle processor, a panel of 200 antigens including these ten were coupled to bead regions for identification. They were then incubated with the participant sera diluted at 1/400. After 3 washes, those complexes were incubated with a secondary antigen conjugated to R-phycoerythrin specific to IgG (Jackson Immunoresearch, UK) for quantification. After another round of washing and resuspension, samples were analysed by the Intelliflex system and results were given in Median Fluorescence Intensity (MFI). To take into account inter plate variability, MFI were standardised by dividing by the MFI value of a 1/400 dilution of a pool of sera exposed to malaria analysed on the same plate. These Normalized MFI (nMFI) were then used for analysis.

#### Samples.

Dielmo is a village of about 400 inhabitants in western Senegal, north of the Gambian border, about four hours’ drive from the capital Dakar. The village is composed of traditional houses built with mud walls and villagers are mostly settled agricultural farmers. Small herds of cattle are also present in the vicinity of habitations. Malaria transmission is perennial [25, 34]. The Sudanian savanna climate of this region combined with the vegetation of the marshy banks of the river Nema support the presence of mosquitoes all year long, mostly *An gambiae* and *An funestus*. The majority of malaria cases are caused by *P. falciparum*, with a small proportion caused by *P. malariae* and *P. ovale*. At the time of initiation of the project in Dielmo, malaria transmission was holoendemic and prevalence was extremely high (86% of children in 1989 [34]), but it dropped in the following decades (<1% of children in 2012 [25]). Another village, Ndiop, was included in the study a few years later. Located a few kilometres south, Ndiop is very similar to Dielmo apart from the fact that transmission is concentrated during the rainy season (end of June to mid-October) [25].

Cross-sectional surveys to investigate the acquisition and maintenance of natural immunity to malaria have been routinely conducted as part of a prospective longitudinal study in Dielmo and Ndiop villages in Senegal over the past 30 years [35–39]. Blood samples were collected via venous bleed or finger prick from all consenting participants. Plasma and red blood cells were stored at -20°C after separation by centrifugation. This study focuses on the cross-sections from 2016 and 2018. In both villages, sampling was performed at the end of the dry season from May to June 2016, and then at the beginning of the dry season in December 2018.

In parallel, a prospective longitudinal study conducted between 1990 and 2012 in Dielmo recorded all cases of fever due to *P. falciparum* infection. Results from this systematic recording were given in number of cases per person per year. The treatment administered in case of infection depended on the national guidelines at the time and long lasting insecticides treated bed nets were introduced in the population during this period.

In 2020, a cohort was conducted in Oise (France) to study seroprevalence to SARS-CoV-2. Children and adults (n = 1,132) attending hospital were recruited and blood samples drawn for routine medical care have been used in previous study for serological surveillance [32]. In this study, a subset of 84 randomly selected samples were used as negative controls as participants are considered to not have been exposed to *P. falciparum*. Only adults were part of this sub sample with a median age of 53 [21, 90] years old, 44% of them were males.

### B. Modelling

#### Seropositivity classification.

As serocatalytic models are fitted to seroprevalence data, continuous antibody levels measured in nMFI must be dichotomized as seropositive or seronegative to each antigen. A Gaussian Mixture Model was fitted to data on measured antibody responses, assuming that antibody levels in a population are a combination of two log normal distributions (one for the positive samples and one for the negatives). Here, Senegalese samples from both villages, whose serostatus is unknown, and French negative controls were included in the model allowing to account for all available information. Having negative controls was also helpful to inform the model in cases where the bimodal pattern was not clearly defined in the Senegalese data.

Overall, the data can be described as such:


$$\begin{array}{c} \left\{ \begin{array}{c} @c{AB}_{unkown}\sim (\text{1}-\;\theta )N\left(\mu_{neg},\;\sigma_{neg}\right)+\theta N\left(\mu_{pos},\;\sigma_{pos}\right)\\ {AB}_{neg}\sim N\left(\mu_{neg},\;\sigma_{neg}\right) \end{array}\right. \end{array}$$

where ${AB}_{unkown}$ is the measured antibody response of Senegalese samples, ${AB}_{neg}$ the one of French negative controls, θ the proportion of positive samples in the Senegalese population and $\mu_{neg}$, $\sigma_{neg}$, $\mu_{pos}$, $\sigma_{pos}$ the mean and standard deviation of the distribution of the antibody levels of the negative and positive samples respectively.

The estimated distributions were subjected to a Receiver Operating Characteristics (ROC) analysis to assess the trade-off between sensitivity and specificity. Sensitivity was estimated as the proportion of the CDF for the positive distribution higher than a selected cutoff, and specificity as the proportion of the CDF for the negative distribution below the selected cutoff. Three cutoffs were considered: one corresponding to the Youden Index, one corresponding to the estimated mean + 2*sd and one corresponding to a specificity of 99% but the later was preferred to the others that had too much variability in sensitivity and specificity between antigens. Calculating an analytical ROC curve based on the cumulative distribution functions of the estimated negative and positive distributions has been shown to have very similar results as an empirical ROC curve built on observed negative and positives samples [40].

### Serocatalytic model

Once the data are dichotomized, it is possible to reconstruct historic exposure by studying the age-stratified seroprevalence in this population. If we assume constant seroconversion rate λ and seroreversion rate ρ the proportion of a population that are seropositive at age *a* can be described by the following ODE system:


$$\begin{array}{c} \left\{ \begin{array}{c} @l\frac{dP_{A}}{da}=\;\lambda P_{\text{0}}-\rho P_{A}\\ P_{\text{0}}+\;P_{A}=\text{1} \end{array}\right. \end{array}$$

where $P_{A}$ and $P_{\text{0}}$ are the probabilities to be seropositive to antigen A and seronegative respectively at a given age. These equations can be solved analytically to give $P_{A}=\;\frac{\lambda }{\lambda +\rho }(\text{1}-\;e^{-(\lambda +\rho )a})$. Under this model, seroprevalence increases rapidly with age among younger participants before reaching an equilibrium prevalence of $\frac{\lambda }{\lambda +\rho }$. To study the impact of different public health interventions, two additional parameters were included: tc the time at which a sharp drop in transmission occurred and $\frac{\text{1}}{\Delta }$ the magnitude of this drop. At a given age i, the contribution to the likelihood is modeled as $N_{i}^{P_{A}}\sim \;Binom(N_{i}^{tot},\;P_{A})$.

To simultaneously analyse data from antibody responses to multiple antigens, we extended existing serocatalytic models. A model of the seroprevalence of two antigens can be described by the following system of ODEs:


$$\begin{array}{c} \left\{ \begin{array}{c} \frac{dP_{\text{0}}}{da}=\;\rho_{A}P_{A}+\;\rho_{B}P_{B}+(-\lambda +\lambda (\text{1}-\gamma_{A})(\text{1}-\gamma_{B}))P_{\text{0}}\\ \frac{dP_{A}}{da}=\;\lambda \gamma_{A}\left(\text{1}-\;\gamma_{B}\right)P_{\text{0}}+\rho_{B}P_{AB}-(\rho_{A}+\lambda \gamma_{B})P_{A}\\ \frac{dP_{B}}{da}=\;\lambda {(\text{1}-\gamma }_{A})\gamma_{B}P_{\text{0}}+\rho_{A}P_{AB}-(\rho_{B}+\lambda \gamma_{A})P_{B}\\ \frac{dP_{AB}}{da}=\;\lambda \gamma_{A}\gamma_{B}P_{\text{0}}+\lambda \gamma_{B}P_{A}+\lambda \gamma_{A}P_{B}-(\rho_{B}+\rho_{A})P_{AB} \end{array}\right. \end{array}$$

We assume a common serological incidence λ for all antibody responses. New parameters γ are included to estimate the probability of seroconversion if exposed. The seroconversion rate for antibody A is thus $\lambda \gamma_{A}$. Seroconversion depends on a subject being exposed to a pathogen and this exposure leading to increased antibody response causing a change of serostatus. The incidence and the probability of seroconverting are combined into the seroconversion rate in univariate serocatalytic models. In multiplexed models, λ is the incidence and the probability of seroconverting to antigen A after exposure is represented by $\gamma_{A}$. Identifiability depends on the balance between the additional information provided by each antigen and their correlation.

The equations for a model with three antigens as well as schema of the compartmental models are provided in the supplementary appendix. In the same way as in the model with a single antigen, the age-specific contribution to the likelihood is modelled using a multinomial distribution over serostatus. Once models were defined, a simulation/recapture study was conducted to assess the capacity of the model and inference framework to correctly estimate each parameter (S1 Appendix *III. b*.).

In this study, data from two cross-sections are available, one in 2016 and one in 2018. They were modelled simultaneously, with the same transmission pattern shifted by 2 years. Apart from shared parameters, the likelihood of each cross-section is independent and modelled using multinomial distributions. Every sample contributes equally to the likelihood despite some participants being included in both cohorts.

#### Implementation.

Models were implemented in a Bayesian framework using Stan (version 2.32.2). Posterior distributions of the estimated parameters were obtained via Markov Chain Monte Carlo (MCMC) sampling, with four parallel chains run for 4,000 iterations each, including 1,000 warm-up iterations. Convergence was assessed for each model by graphical assessment of the mixing of the chains and posterior distributions. Visual predictive checks were used as well to ensure the adequacy of the model to the data.

ODE systems were solved using the ode_rk45 function in stan. Age was binned to one year for computational efficiency.

Most priors were as weakly informative as possible while being relevant: tc~uniform(0,50), γ~Beta(3,1.3) and λ~exponential(1). For ρ, priors are specific to each antibody response. As stated previously, samples from children under five years old from Dielmo could not be analysed. Yet in serocatalytic models, λ and ρ are very correlated and are mostly informed by the younger age data which caused convergence issues. ρ is antigen dependent only and should be concordant in two similar populations. The choice was thus made to fit a simple serocatalytic model including one sharp drop in transmission to Ndiop data and use the posterior distribution as prior for ρ.

#### Protocol.

Once the priors for ρ were described from a simple serocatalytic model, they were applied to all combination of 1, 2 or 3 antibody responses observed in Dielmo out of the 6 that showed no convergence issues in Ndiop. Models that converged were considered for external validation. Finally, a Kruskal-Wallis ranking test was conducted to assess the importance of taking into account more than one antibody response at a time (*Supplementary III.c*).

#### External validation.

Data from Trape *et al* were digitized using the Pixel Ruler v3.1 app. The digitized dataset including pixel measurement can be found in the supplementary files. The number of *P. falciparum* per person per year was used as external validation for parameters estimated from the different models. For each model, a conversion coefficient was applied to relate the seroconversion rate over time to the number of clinical cases per person year. This was calculated by minimizing the mean standard error (MSE) between the data on clinical cases and the modelled seroconversion rate times the conversion coefficient. This coefficient was then used on the whole range of the posterior distribution to recapture uncertainty. MSE computed from the median and IC95% were then compared to select the best model to recapture the past implementation of public health interventions. In this framework, only tc and Δ are validated as λ has been converted and ρ can not be recaptured by the validation data.

### Financial disclosure

This work was supported by the European Research Council (MultiSeroSurv, 852373 to MW), and the French government’s “Integrative Biology of Emerging Infectious Diseases” (Investissement d’Avenir grant ANR-10-LABX-62-IBEID to MW) and INCEPTION programs (Investissement d’Avenir grant ANR-16-CONV-0005 to MW). The funders had no role in study design, data collection and analysis, decision to publish, or preparation of the manuscript.

## Supporting information

S1 Appendix Supporting information. (DOCX)

S1 Data Antibody responses to *P. falciparum* in Dielmo and N’diop. (CSV)

S2 Data Digitized incidence to *P. falciparum* in Dielmo. (CSV)
