# Supplementary material for: Reconstruction of historical malaria transmission in Senegal using multiplex serocatalytic models
Source: PLoS Comput Biol. 2026 Jul 27;22(7):e1013630. doi: 10.1371/journal.pcbi.1013630 (PMC13423172; doi:10.1371/journal.pcbi.1013630)
Supplement: S1 Appendix — (DOCX) [file pcbi.1013630.s001.docx]

**Supporting Information –** **Reconstruction of historical malaria transmission in Senegal using multiplex serocatalytic models**.

Gaëlle Baudemont^1^, Thomas Obadia^1,2^, Laura Garcia^1^, Camille Lambert^1^, Françoise Donnadieu^1^, Fatoumata Diene Sarr^3^, Joseph Faye^3^, Cheikh Sokhna^4^, Inès Vigan-Womas^5^, Aissatou Toure-Balde^5^, Chris Drakeley^6^, Makhtar Niang^7^, Michael White^1^*

1. Infectious Disease Epidemiology and Analytics Unit, Department of Global Health, Université Paris Cité, INSERM U1347, Institut Pasteur, Paris, France
2. Hub de Bioinformatique et Biostatistique, Département Biologie Computationnelle, Institut Pasteur, USR 3756 CNRS, Paris, France.
3. Pôle Epidemiology, Clinical Research and Data Science, Institut Pasteur de Dakar, Dakar, Sénégal
4. Institut de Recherche pour le Développement, Laboratoire de Paludologie, Dakar, Sénégal
5. Pôle Immunophysiopathologie & Maladies Infectieuses, Institut Pasteur de Dakar, Dakar, Sénégal
6. Department of Infection Biology, London School of Hygiene and Tropical Medicine, London, UK
7. Immunophysiopathology and Infectious Diseases Department, Institut Pasteur de Dakar; 220 Dakar, Sénégal
8. Antigens

| **Name** | **Description** | **Location** | **Expression tag** |
| --- | --- | --- | --- |
| ***PfCSP*** | Circumsporozoite protein | Sporozoites | GST |
| ***PfGlurpR2*** | Glutamate Rich Protein C-terminal repetitive segment | Merozoites surface | GST |
| ***PfAMA1*** | Apical Membrane Antigen 1 | Blood stage merozoites / sporozoites | His |
| ***PfMSP1*** | Merozoite Surface Protein 1 | Merozoites surface | GST |
| ***PfEtramp4*** | Early Transcribed Membrane Protein 4 | Infected red blood cell | GST |
| ***PfHSP40*** | Heat Shock Protein 40 | Infected red blood cell | GST |
| ***PfMSP2-Dd2*** | Merozoite Surface Protein 2 (Dd2 allele) | Merozoites surface | GST |
| ***PfMSP2-CH150*** | Merozoite Surface Protein 2 (CH150 allele) | Merozoites surface | GST |
| ***PfSEA1*** | Schizont Egress Antigen 1 | Infected red blood cell | GST |
| ***PfSBP1*** | Skeleton Binding Protein 1 | Infected red blood cell | GST |

***Table A: Antigens description.*** *Characteristics of the 10 antigens analysed in this study.*

1. Data
   1. Observations

| **Seroprevalence (%)** | | | |
| --- | --- | --- | --- |
|  | **Dielmo** | **Ndiop** | **p-value** |
| ***PfCSP*** | **37** | **23** | **2.8e-07 ***** |
| ***5–10-year-old*** | 7 | 6 | 0.9 |
| ***10–20-year-old*** | 17 | 6 | 0.002 ** |
| ***20+ year-old*** | 59 | 48 | 0.008 ** |
| ***PfGlurpR2*** | **47** | **37** | **0.0007 ***** |
| ***5–10-year-old*** | 10 | 3 | 0.06 |
| ***10–20-year-old*** | 22 | 16 | 0.2 |
| ***20+ year-old*** | 75 | 75 | 1 |
| ***PfAMA1*** | **47** | **37** | **0.001 **** |
| ***5–10-year-old*** | 10 | 2 | 0.01 * |
| ***10–20-year-old*** | 26 | 16 | 0.02 * |
| ***20+ year-old*** | 72 | 76 | 0.3 |
| ***PfMSP1*** | **54** | **54** | **1** |
| ***5–10-year-old*** | 28 | 37 | 0.2 |
| ***10–20-year-old*** | 36 | 46 | 0.06 |
| ***20+ year-old*** | 75 | 78 | 0.4 |
| ***PfEtramp4*** | **27** | **13** | **1.1e-09 ***** |
| ***5–10-year-old*** | 14 | 4 | 0.02 * |
| ***10–20-year-old*** | 16 | 8 | 0.03 * |
| ***20+ year-old*** | 39 | 24 | 0.0002 *** |
| ***PfHSP40*** | **17** | **13** | **0.07** |
| ***5–10-year-old*** | 5 | 4 | 1 |
| ***10–20-year-old*** | 9 | 6 | 0.2 |
| ***20+ year-old*** | 26 | 26 | 1 |
| ***PfMSP2-Dd2*** | **32** | **21** | **1.8e-05 ***** |
| ***5–10-year-old*** | 15 | 3 | 0.004 ** |
| ***10–20-year-old*** | 17 | 8 | 0.01 * |
| ***20+ year-old*** | 47 | 42 | 0.3 |
| ***PfMSP2-CH150*** | **3** | **0.6** | **0.0008 ***** |
| ***5–10-year-old*** | 3 | 0 | 0.1 |
| ***10–20-year-old*** | 2 | 1 | 0.9 |
| ***20+ year-old*** | 4 | 0.7 | 0.01 * |
| ***PfSEA1*** | **12** | **10** | **0.5** |
| ***5–10-year-old*** | 10 | 5 | 0.2 |
| ***10–20-year-old*** | 11 | 9 | 0.6 |
| ***20+ year-old*** | 13 | 15 | 0.4 |
| ***PfSBP1*** | **11** | **9** | **0.3** |
| ***5–10-year-old*** | 9 | 4 | 0.2 |
| ***10–20-year-old*** | 11 | 9 | 0.5 |
| ***20+ year-old*** | 12 | 13 | 0.7 |

***Table B***. **Overall and age stratified observed seroprevalence by village**. Group differences were assessed using $\mathcal{X}^{2}$ tests using the *chisq.test* function in R and p-values are reported in the table.

| **Seroprevalence (%)** | | | |
| --- | --- | --- | --- |
|  | **2016** | **2018** | **p-value** |
| ***PfCSP*** | **30** | **27** | **0.3** |
| ***5–10-year-old*** | 7 | 5 | 0.9 |
| ***10–20-year-old*** | 15 | 7 | 0.02 * |
| ***20+ year-old*** | 57 | 49 | 0.08 |
| ***PfGlurpR2*** | **41** | **41** | **1** |
| ***5–10-year-old*** | 8 | 4 | 0.5 |
| ***10–20-year-old*** | 24 | 12 | 0.004 ** |
| ***20+ year-old*** | 76 | 74 | 0.7 |
| ***PfAMA1*** | **43** | **38** | **0.1** |
| ***5–10-year-old*** | 8 | 1 | 0.04 * |
| ***10–20-year-old*** | 29 | 10 | 1.2e-05 *** |
| ***20+ year-old*** | 77 | 71 | 0.1 |
| ***PfMSP1*** | **56** | **52** | **0.2** |
| ***5–10-year-old*** | 35 | 31 | 0.6 |
| ***10–20-year-old*** | 46 | 36 | 0.05 |
| ***20+ year-old*** | 82 | 71 | 0.003 ** |
| ***PfEtramp4*** | **21** | **17** | **0.2** |
| ***5–10-year-old*** | 8 | 9 | 1 |
| ***10–20-year-old*** | 13 | 10 | 0.4 |
| ***20+ year-old*** | 36 | 25 | 0.01 * |
| ***PfHSP40*** | **17** | **12** | **0.02 *** |
| ***5–10-year-old*** | 6 | 2 | 0.3 |
| ***10–20-year-old*** | 10 | 4 | 0.04 * |
| ***20+ year-old*** | 30 | 21 | 0.03 * |
| ***PfMSP2-Dd2*** | **28** | **23** | **0.03 *** |
| ***5–10-year-old*** | 12 | 3 | 0.04 * |
| ***10–20-year-old*** | 16 | 7 | 0.01 * |
| ***20+ year-old*** | 49 | 40 | 0.04 * |
| ***PfMSP2-CH150*** | **3** | **0.4** | **0.004 **** |
| ***5–10-year-old*** | 3 | 0 | 0.3 |
| ***10–20-year-old*** | 2 | 0.6 | 0.4 |
| ***20+ year-old*** | 4 | 0.4 | 0.01 * |
| ***PfSEA1*** | **11** | **10** | **0.6** |
| ***5–10-year-old*** | 6 | 9 | 0.6 |
| ***10–20-year-old*** | 12 | 7 | 0.2 |
| ***20+ year-old*** | 15 | 13 | 0.5 |
| ***PfSBP1*** | **11** | **9** | **0.3** |
| ***5–10-year-old*** | 5 | 7 | 0.7 |
| ***10–20-year-old*** | 12 | 7 | 0.2 |
| ***20+ year-old*** | 15 | 10 | 0.1 |

***Table C***. **Overall and age stratified observed seroprevalence by cohort**. Group differences were assessed using $\mathcal{X}^{2}$ tests using the *chisq.test* function in R and p-values are reported in the table.

- 1. Classification


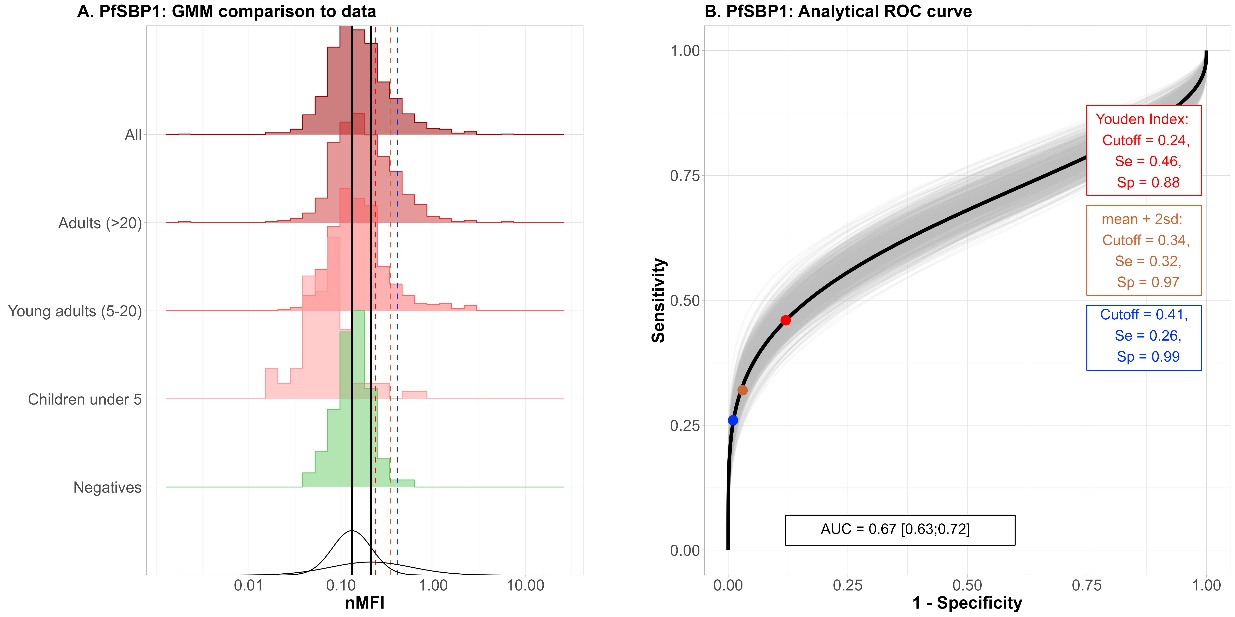


***Figure A: GMM fitted to PfSBP1.*** *Panel A shows the distribution of PfSBP1 response compared to the bimodal distribution estimated by the GMM. Black vertical lines are the estimated means of the components representing the negatives and positives samples. Dashed coloured lines are the considered cutoffs. Panel B shows the analytical ROC curve from the GMM fitted on PfSBP1 as well as the different cutoffs considered and their associate sensitivity, specificity.*

**
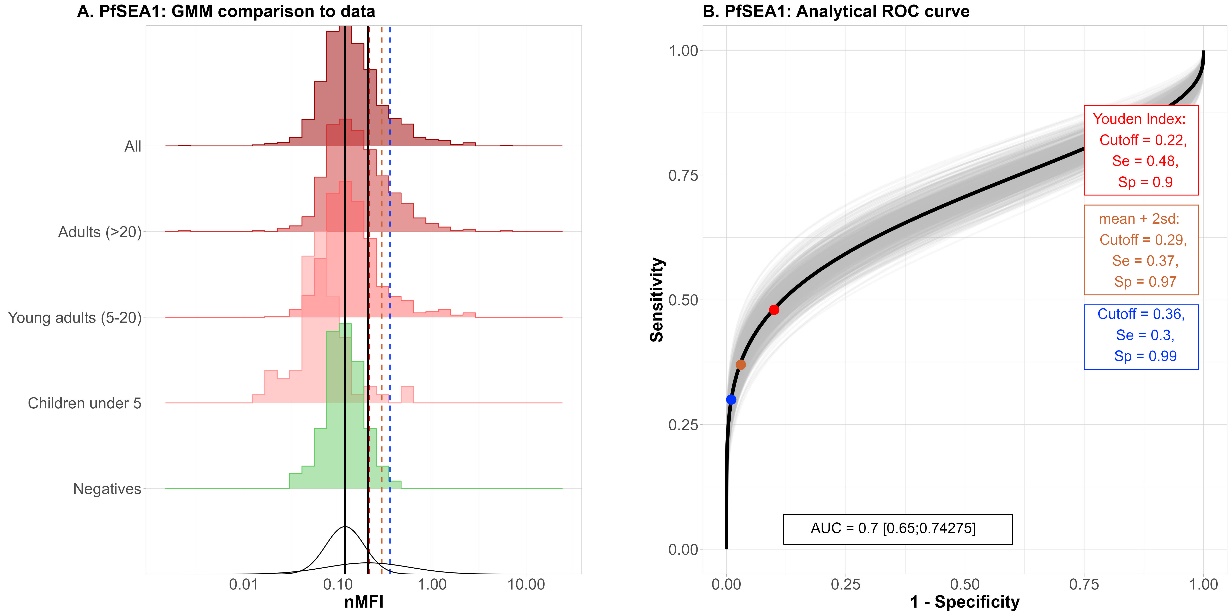
**

***Figure B: GMM fitted to PfSEA1.*** *Panel A shows the distribution of PfSEA1 response compared to the bimodal distribution estimated by the GMM. Black vertical lines are the estimated means of the components representing the negatives and positives samples. Dashed coloured lines are the considered cutoffs. Panel B shows the analytical ROC curve from the GMM fitted on PfSEA1 as well as the different cutoffs considered and their associate sensitivity, specificity.*


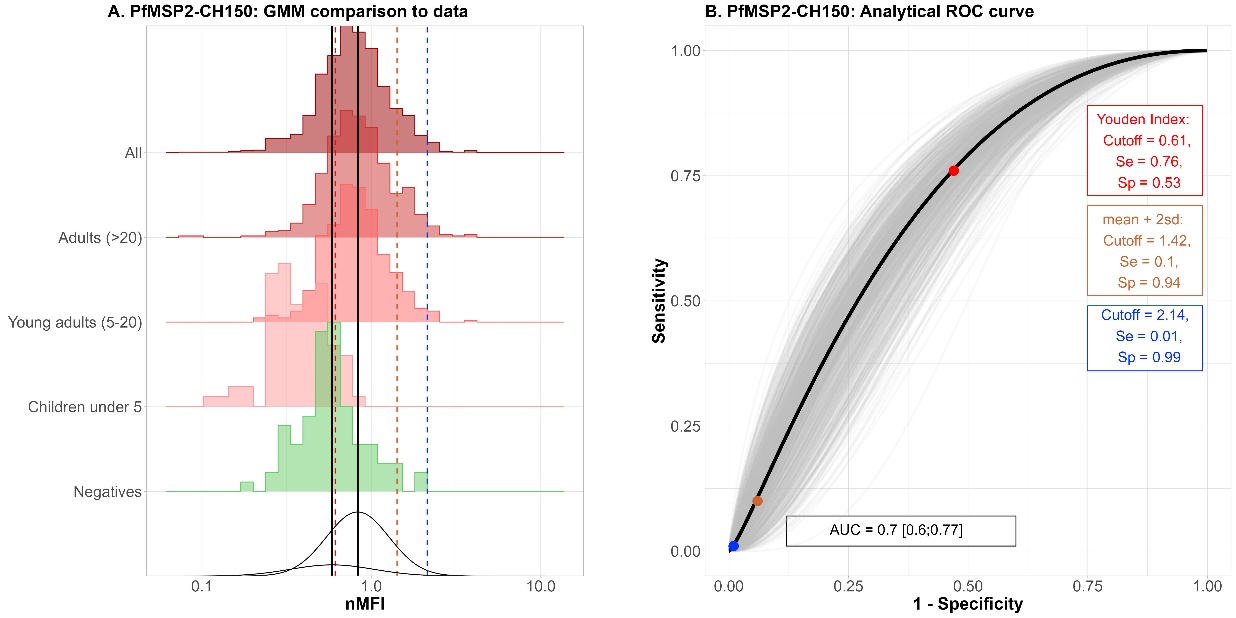


***Figure C: GMM fitted to PfMSP2-CH150.*** *Panel A shows the distribution of PfMSP2-CH150 response compared to the bimodal distribution estimated by the GMM. Black vertical lines are the estimated means of the components representing the negatives and positives samples. Dashed coloured lines are the considered cutoffs. Panel B shows the analytical ROC curve from the GMM fitted on PfMSP2-CH150 as well as the different cutoffs considered and their associate sensitivity, specificity.*


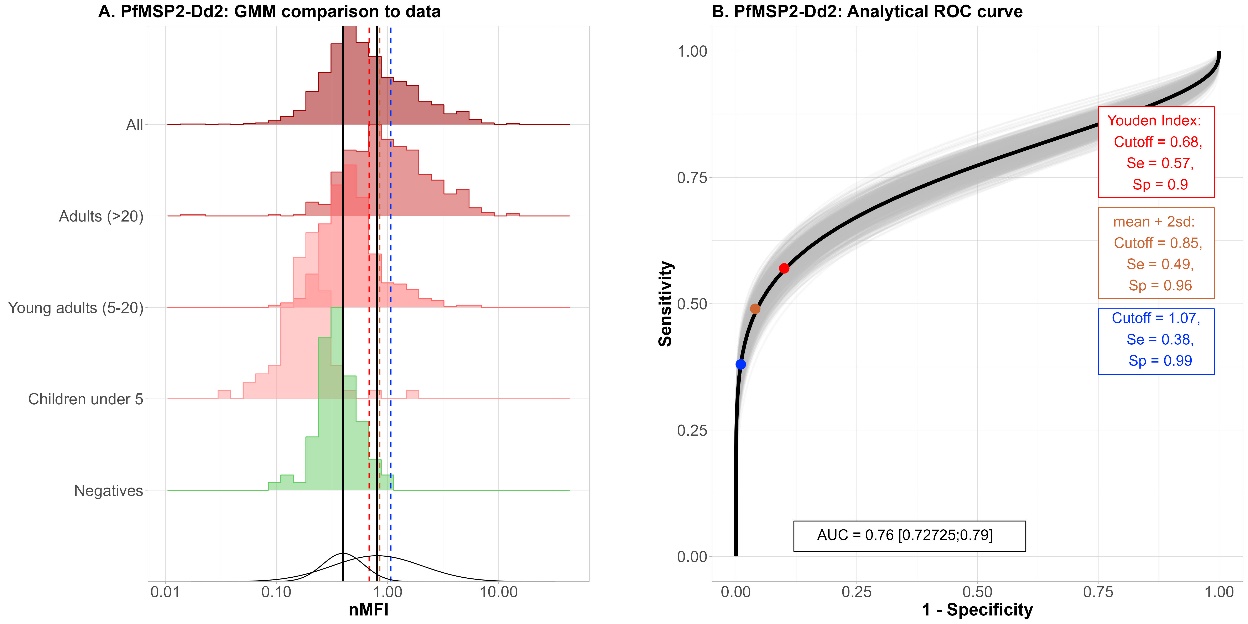


***Figure D: GMM fitted to PfMSP2-Dd2.*** *Panel A shows the distribution of PfMSP2-Dd2 response compared to the bimodal distribution estimated by the GMM. Black vertical lines are the estimated means of the components representing the negatives and positives samples. Dashed coloured lines are the considered cutoffs. Panel B shows the analytical ROC curve from the GMM fitted on PfMSP2-Dd2 as well as the different cutoffs considered and their associate sensitivity, specificity.*


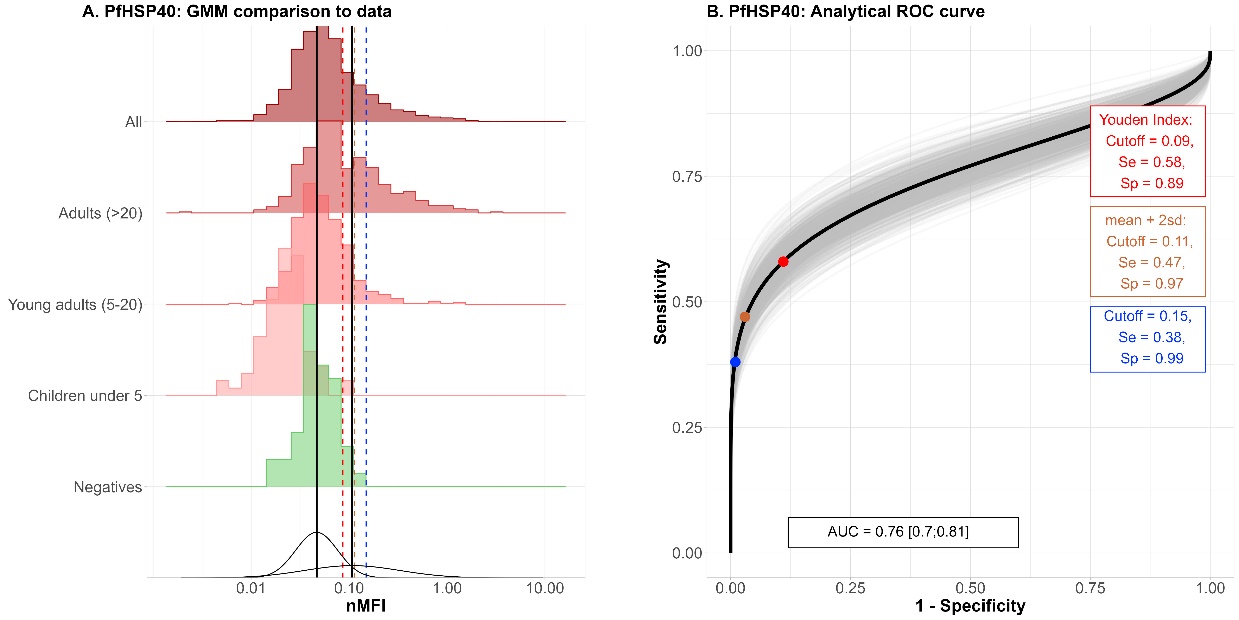


***Figure E: GMM fitted to PfHSP40.*** *Panel A shows the distribution of PfHSP40 response compared to the bimodal distribution estimated by the GMM. Black vertical lines are the estimated means of the components representing the negatives and positives samples. Dashed coloured lines are the considered cutoffs. Panel B shows the analytical ROC curve from the GMM fitted on PfHSP40 as well as the different cutoffs considered and their associate sensitivity, specificity.*


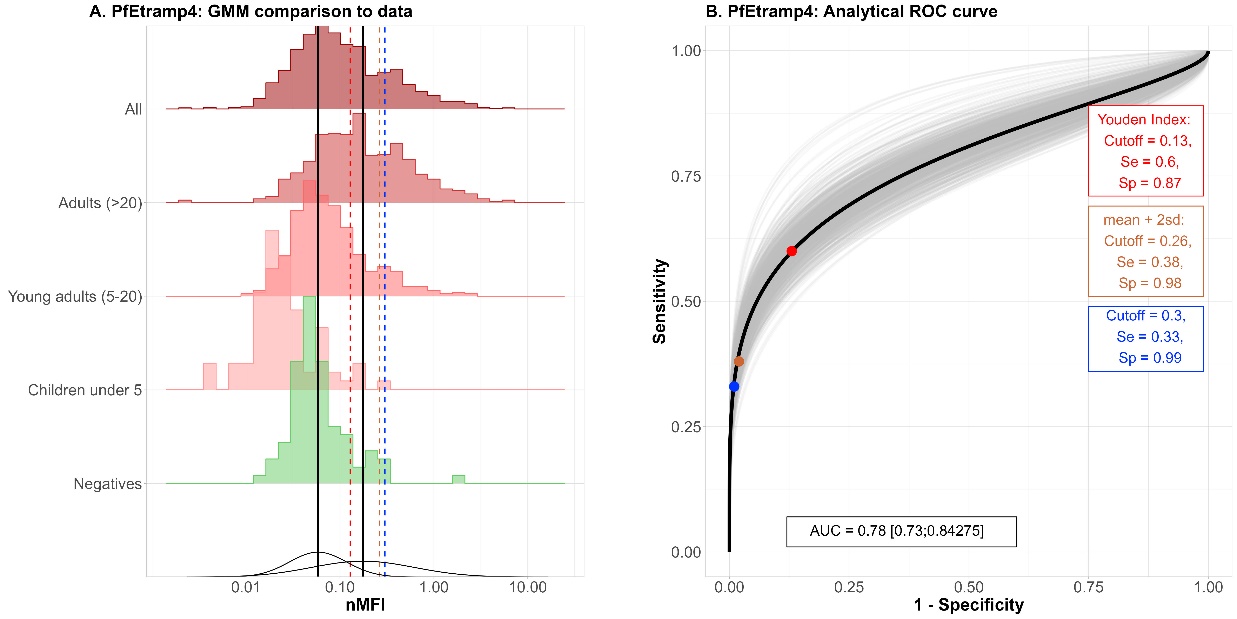


***Figure F: GMM fitted to PfEtramp4.*** *Panel A shows the distribution of PfEtramp4 response compared to the bimodal distribution estimated by the GMM. Black vertical lines are the estimated means of the components representing the negatives and positives samples. Dashed coloured lines are the considered cutoffs. Panel B shows the analytical ROC curve from the GMM fitted on PfEtramp4 as well as the different cutoffs considered and their associate sensitivity, specificity.*


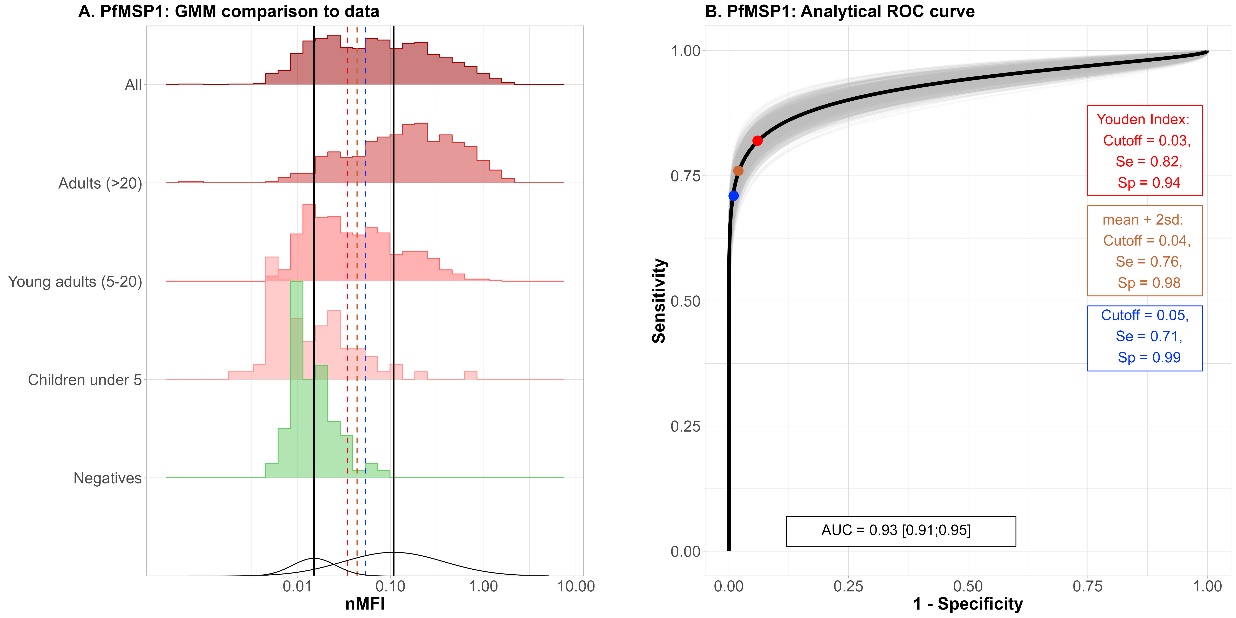


***Figure G: GMM fitted to PfMSP1.*** *Panel A shows the distribution of PfMSP1 response compared to the bimodal distribution estimated by the GMM. Black vertical lines are the estimated means of the components representing the negatives and positives samples. Dashed coloured lines are the considered cutoffs. Panel B shows the analytical ROC curve from the GMM fitted on PfMSP1 as well as the different cutoffs considered and their associate sensitivity, specificity.*


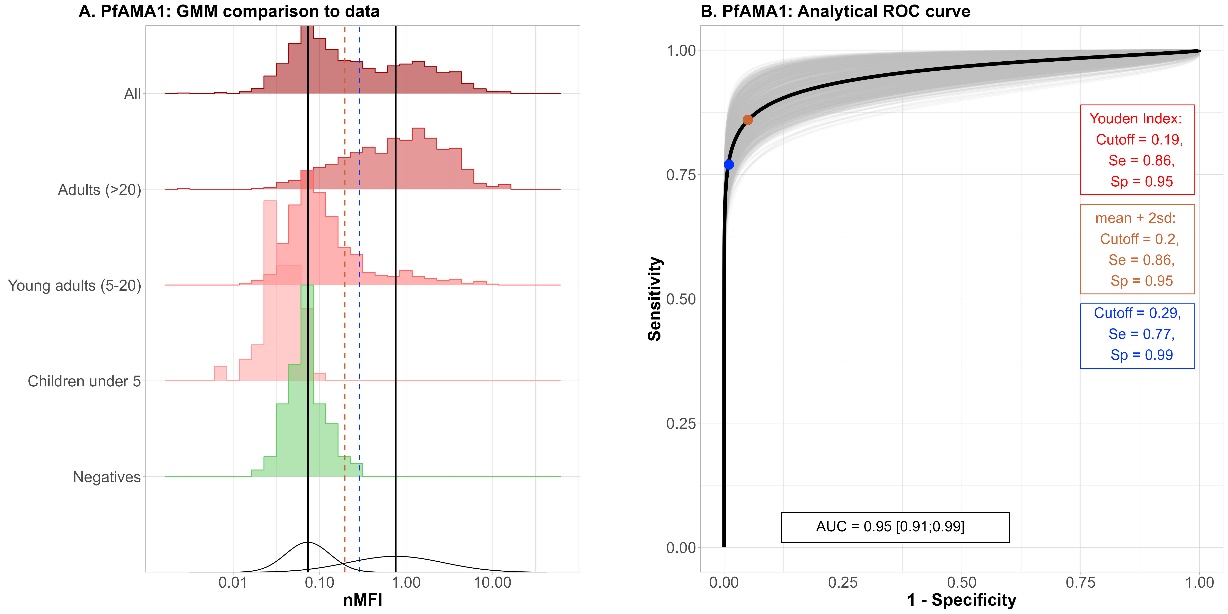


***Figure H: GMM fitted to PfAMA1.*** *Panel A shows the distribution of PfAMA1 response compared to the bimodal distribution estimated by the GMM. Black vertical lines are the estimated means of the components representing the negatives and positives samples. Dashed coloured lines are the considered cutoffs. Panel B shows the analytical ROC curve from the GMM fitted on PfAMA1 as well as the different cutoffs considered and their associate sensitivity, specificity.*


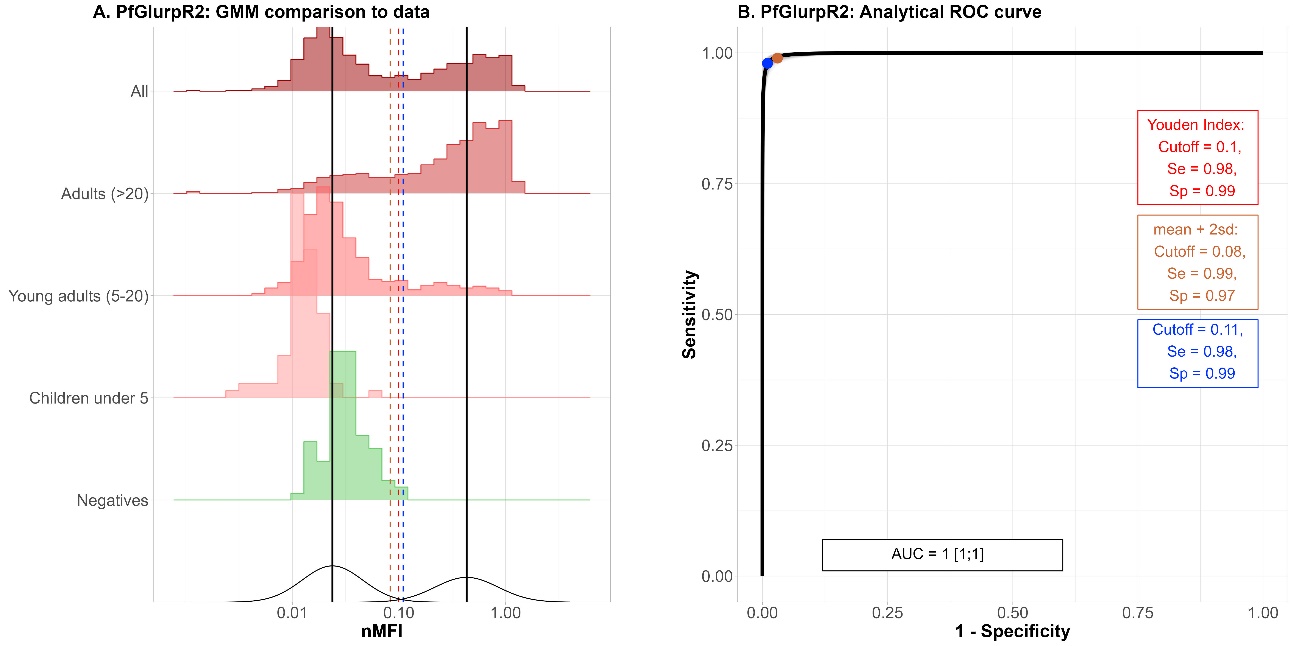


***Figure I: GMM fitted to PfGlurpR2.*** *Panel A shows the distribution of PfGlurpR2 response compared to the bimodal distribution estimated by the GMM. Black vertical lines are the estimated means of the components representing the negatives and positives samples. Dashed coloured lines are the considered cutoffs. Panel B shows the analytical ROC curve from the GMM fitted on PfGlurpR2 as well as the different cutoffs considered and their associate sensitivity, specificity.*

- 1. Validation data set

Data from Figure 3.C of <https://doi.org/10.1016/S1473-3099(14)70712-1> were digitized using the Pixel Ruler v3.1 app. For each of the 23 years, the number of pixels comprising the bar representing the number of fevers per person per year caused by *P. falciparum* was measured. The measurement resolution, determined by the pixel-to-data scaling of the y-axis, is 0.11 *P. falciparum* fevers per person per year per pixel. Digitization of a data point was repeated ten times and showed a variability of +/- 1 pixel or 0.11 *P. falciparum* fevers per person per year. The digitized dataset including pixel measurement can be found in the supplementary files.

1. Modelling
   1. Model definition


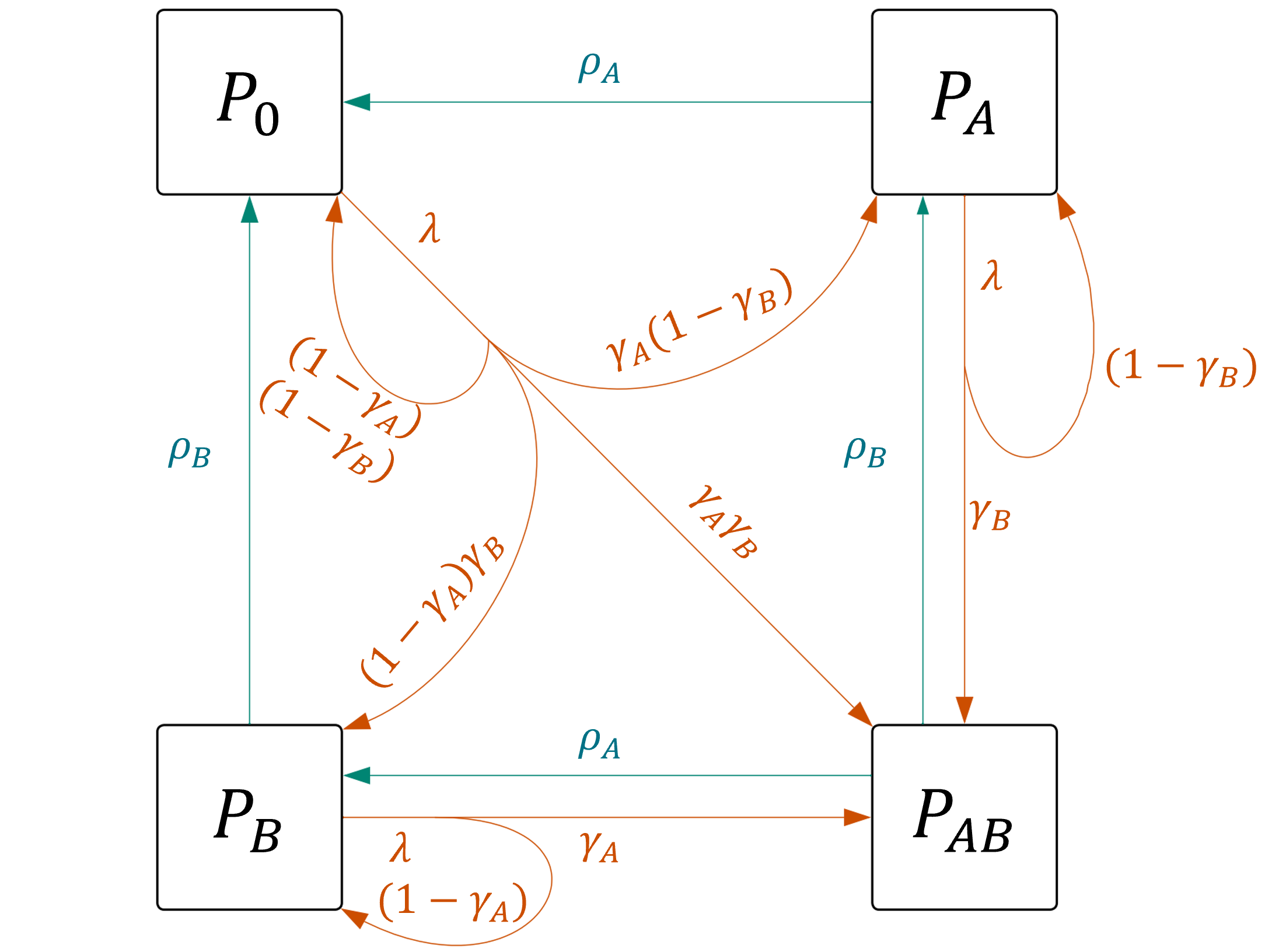


***Figure J: Schematic representation of a serocatalytic model including two antibody responses.*** *Let A and B be two antibodies:* $P_{0}$ *is seronegative,* $P_{A}$ *is positive to A only,* $P_{B}$ *is positive to B only and* $P_{AB}$ *is positive to both. This model considers four possible serostatus, the serological incidence, the probability to seroconvert once exposed (red arrows) for each antibody and waning of each antibody (blue arrows).*

Practical example:

Most adults older than 20 years old are seropositive to both PfAMA1 and *Pf*GlurpR2. After being exposed to *P. falciparum* at rate $\lambda$, 481/560 individuals are seropositive to at least one antigen and most of them (354/481) seroconverted to both *Pf*AMA1 and *Pf*GlurpR2, with 62/481 participants seropositive to PfAMA1 only and 65/481 seropositive to *Pf*GlurpR2 only. The probabilities to seroconvert to a given antigen after exposure are given by the parameters $\gamma_{antigen}$ and the probability to seroconvert to a given serostatus is given by the combination of $\lambda$ and $\gamma_{antigens}$. For example, the probability to seroconvert to PfAMA1 only is the probability of being exposed $\lambda$ multiplied by the probability that exposure caused seroconversion to PfAMA1, $\gamma_{PfAMA1}$, but not to *Pf*GlurpR2, $(1- \gamma_{PfGlurpR2})$.

|  | PfAMA1 | |
| --- | --- | --- |
| *Pf*GlurpR2 | $P_{0}=79$ | $P_{PfAMA1}=62$ |
|  | $P_{PfGlurpR2}=65$ | $P_{PfAMA1, PfGlurpR2}=354$ |

**Table D: Contingency table of the different observed serostatus for the combination of PfAMA1 and *Pf*GlurpR2 and for adults of more than 20 years**. The distribution is significantly different (Pearson’s $\chi^{2}$-test, p-value <2.2e-16) of a distribution reflecting independence of the two antibody responses.


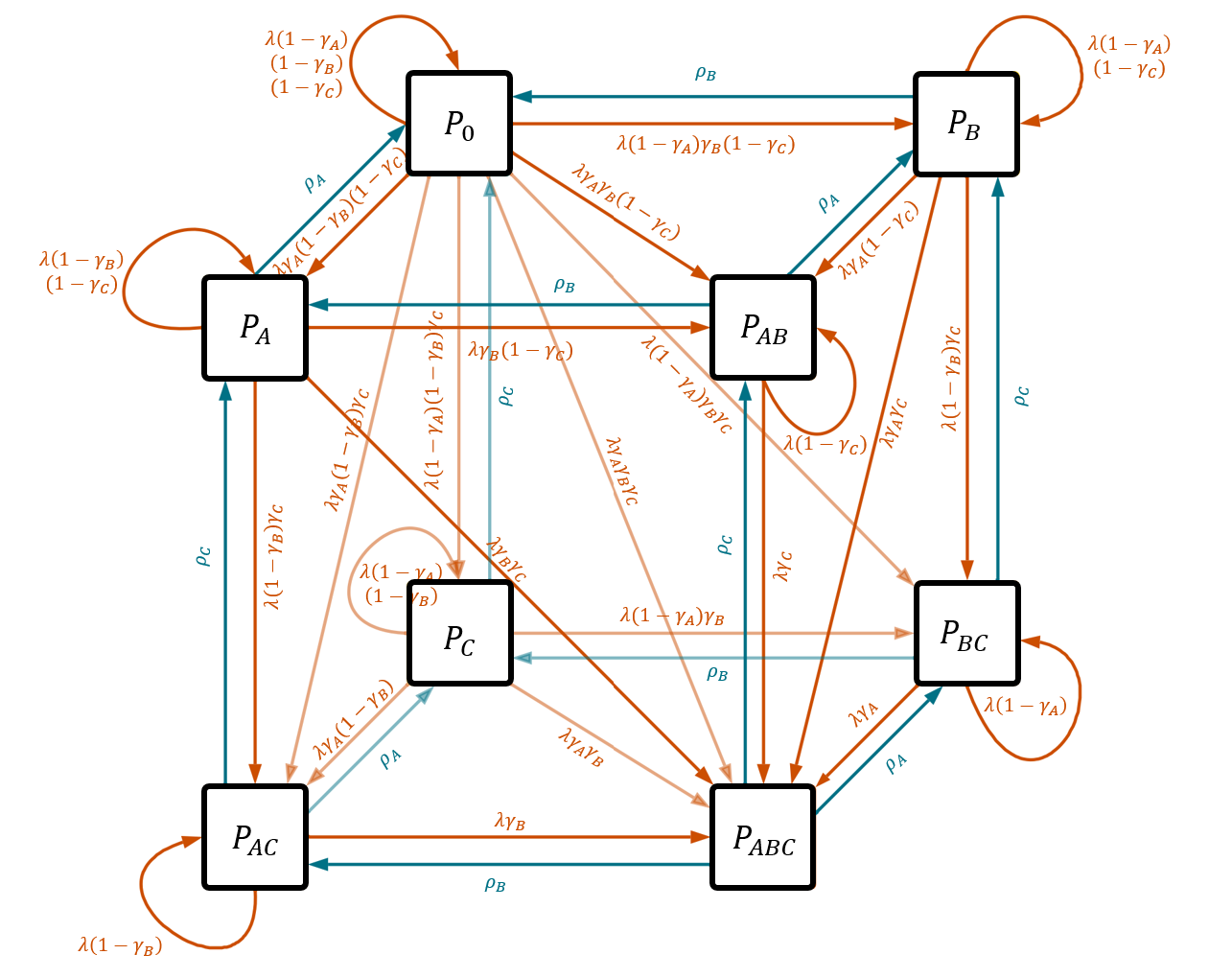


***Figure K: Schematic representation of a serocatalytic model including three antibody responses.*** *Let A, B and C be three antibodies:* $P_{0}$ *is seronegative,* $P_{A}$*,* $P_{B}$ *and* $P_{C}$ *is positive only to A, B or C respectively,* $P_{AB}$*,* $P_{AC}$*,* $P_{BC}$ *are positive to each combination of two out of the three antibodies and* $P_{ABC}$ *is seropositive to A, B and C. This model considers eight possible serostatuses, the serological incidence, the probability to seroconvert once exposed (red arrows) for each antibody and waning of each antibody (blue arrows).*

$$\left\{ \begin{aligned} \frac{dP_{0}}{da}= \rho_{A}P_{A}+ \rho_{B}P_{B}+ \rho_{C}P_{C}+(-\lambda+\lambda(1-\gamma_{A})(1-\gamma_{B})(1-\gamma_{C}))P_{0} \\ \frac{dP_{A}}{da}= \lambda\gamma_{A}\left( 1- \gamma_{B} \right)\left( 1- \gamma_{C} \right)P_{0}+\rho_{B}P_{AB}+\rho_{C}P_{AC}+(-\lambda+\lambda\left( 1-\gamma_{B} \right)\left( 1-\gamma_{C} \right)-\rho_{A})P_{A} \\ \frac{dP_{B}}{da}= \lambda\left( 1-\gamma_{A} \right)\gamma_{B}\left( 1- \gamma_{C} \right)P_{0}+\rho_{A}P_{AB}+\rho_{C}P_{BC}+\left( -\lambda+\lambda\left( 1-\gamma_{A} \right)\left( 1-\gamma_{C} \right)-\rho_{B} \right)P_{B} \\ \frac{dP_{C}}{da}= \lambda(1-\gamma_{A})(1-\gamma_{B})\gamma_{C}P_{0}+\rho_{A}P_{AC}+\rho_{B}P_{BC}+(-\lambda+\lambda\left( 1-\gamma_{A} \right)\left( 1-\gamma_{B} \right)-\rho_{C})P_{C} \\ \frac{dP_{AB}}{da}= \lambda\gamma_{A}{(1-\gamma}_{C})P_{B}+\lambda\gamma_{B}{(1-\gamma}_{C})P_{A}+\rho_{C}P_{ABC}+(-\lambda+\lambda\left( 1-\gamma_{C} \right)-\rho_{B}-\rho_{A})P_{AB} \\ \frac{dP_{BC}}{da}= \lambda{(1-\gamma}_{A})\gamma_{C}P_{B}+\lambda{(1-\gamma}_{A})\gamma_{B}P_{C}+\rho_{A}P_{ABC}+(-\lambda+\lambda\left( 1-\gamma_{A} \right)-\rho_{B}-\rho_{C})P_{BC} \\ \frac{dP_{AC}}{da}= \lambda\gamma_{A}{(1-\gamma}_{B})P_{C}+\lambda{(1-\gamma}_{B})\gamma_{C}P_{A}+\rho_{B}P_{ABC}+(-\lambda+\lambda\left( 1-\gamma_{B} \right)-\rho_{A}-\rho_{C})P_{AC} \\ \frac{dP_{ABC}}{da}= \lambda\gamma_{A}P_{BC}+\lambda\gamma_{B}P_{AC}+\lambda\gamma_{C}P_{AB}+\lambda\gamma_{A}\gamma_{B}P_{C}+\lambda\gamma_{A}\gamma_{C}P_{B}+\lambda\gamma_{B}\gamma_{C}P_{A}+\lambda\gamma_{A}\gamma_{B}\gamma_{C}P_{0}-(\rho_{A}+\rho_{B}+\rho_{C})P_{ABC} \end{aligned} \right.$$

***Equation A: ODE system of a model considering three antibody responses.*** $\lambda$ *is a common serological incidence,* $\gamma_{A}$*,* $\gamma_{B}$*,* $\gamma_{C}$ *are the probabilities to seroconvert if exposed for each antibody and* $\rho_{A}$*,* $\rho_{B}$*,* $\rho_{C}$ *the antibody dependent seroreversion rates.*

- 1. Simulation / Recapture study

Once models were defined, a simulation/recapture study was conducted to assess the capacity of the model and inference framework to correctly estimate each parameter. Populations were simulated assuming a Senegalese age distribution under different scenarios of malaria exposure. The serostatus attributed to each participant was sampled in a multinomial distribution with the probabilities computed by the model to be tested. For each set of parameters (*Table S2*), 20 populations were simulated and the model was fitted on each of them in a Bayesian framework using one Markov Chain of 800 iterations in R stan. The merged posterior distributions of the estimated parameters were then compared to their actual value used for the simulations. The validation of the model was made by visual assessment of those comparisons, plus the Visual Predictive Check (VPC) comparison to the simulated data.

| **Parameter** | **Values tested** |
| --- | --- |
| Serological incidence $\lambda$ (${years}^{-1}$) | 0.01, 0.1, 1 |
| Seroreversion rates $\rho$ (${years}^{-1}$) | 0.01, 0.1 |
| Probability to seroconvert if exposed $\gamma$ | 0.3, 0.8 |
| Time of drop in transmission $tc$ ($years$) | 5, 15 |
| Magnitude of drop in transmission $\frac{1}{\Delta}$ | 0.1, 0.5 |

***Table E: Parameter values tested in the simulation recapture study.*** *The serocatalytic model including two antibody response and one sharp drop in transmission was fitted on 20 populations simulated for each combination of those parameter values.*

More than 80% (51 out of 62) of the models were capable of recapturing the parameters used for simulation (*Figure S12*).


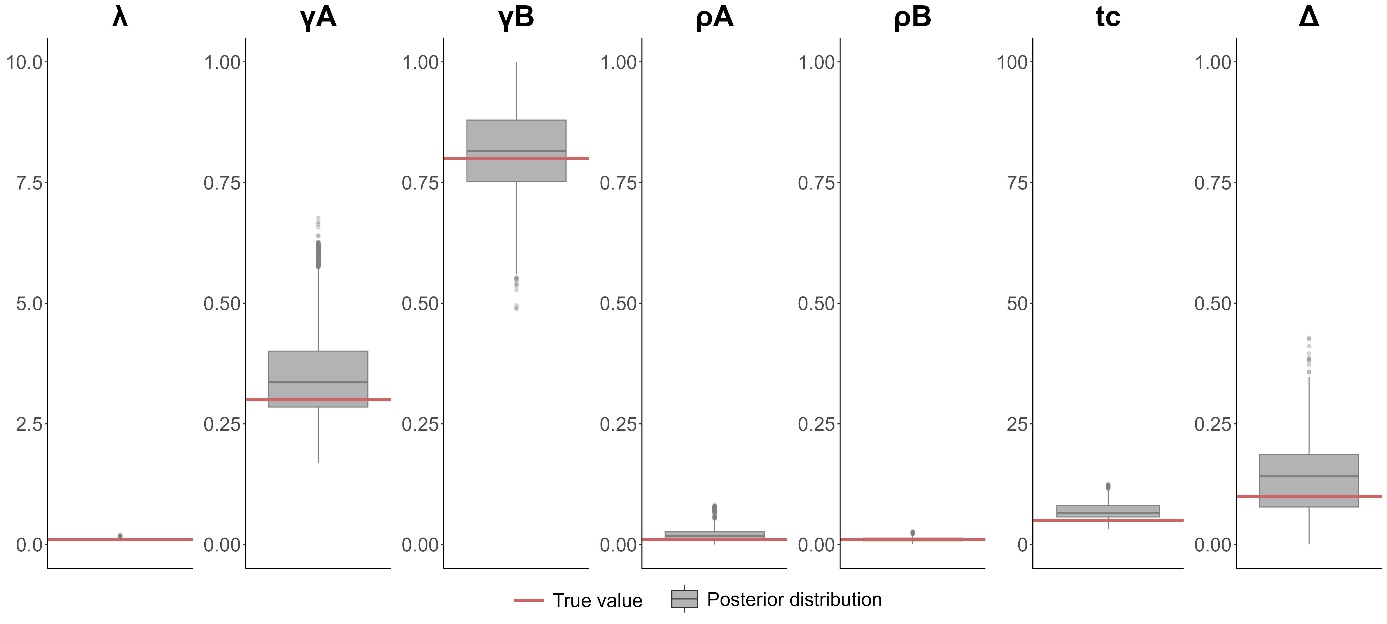


***Figure L: Posterior distributions.*** *The model was fitted to 20 populations simulated with the seroincidence* $\lambda=0.1$*, the probabilities to seroconvert to antigen A and B, respectively* $\gamma_{A}=0.3$ *and* $\gamma_{B}=0.8$*, the seroreversion rates* $\rho_{A}=0.01$ *and* $\rho_{B}=0.01$*, the time of the sharp drop in transmission* $tc=5$ *and a new seroincidence* $\lambda\Delta=0.1*0.1$*. The grey boxplots are the merged posterior distributions of those 20 fits compared to the actual parameter values.*

- 1. Digitization of validation data

Data from Figure 3.C of <https://doi.org/10.1016/S1473-3099(14)70712-1> were digitized using the Pixel Ruler v3.1 app. For each of the 23 years, the number of pixels comprising the bar representing the number of fevers per person per year caused by *P. falciparum* was measured. The measurement resolution, determined by the pixel-to-data scaling of the y-axis, is 0.11 *P. falciparum* fevers per person per year per pixel. Digitization of a data point was repeated ten times and showed a variability of +/- 1 pixel or 0.11 *P. falciparum* fevers per person per year. The digitized dataset including pixel measurement can be found in the supplementary files.

- 1. Framework


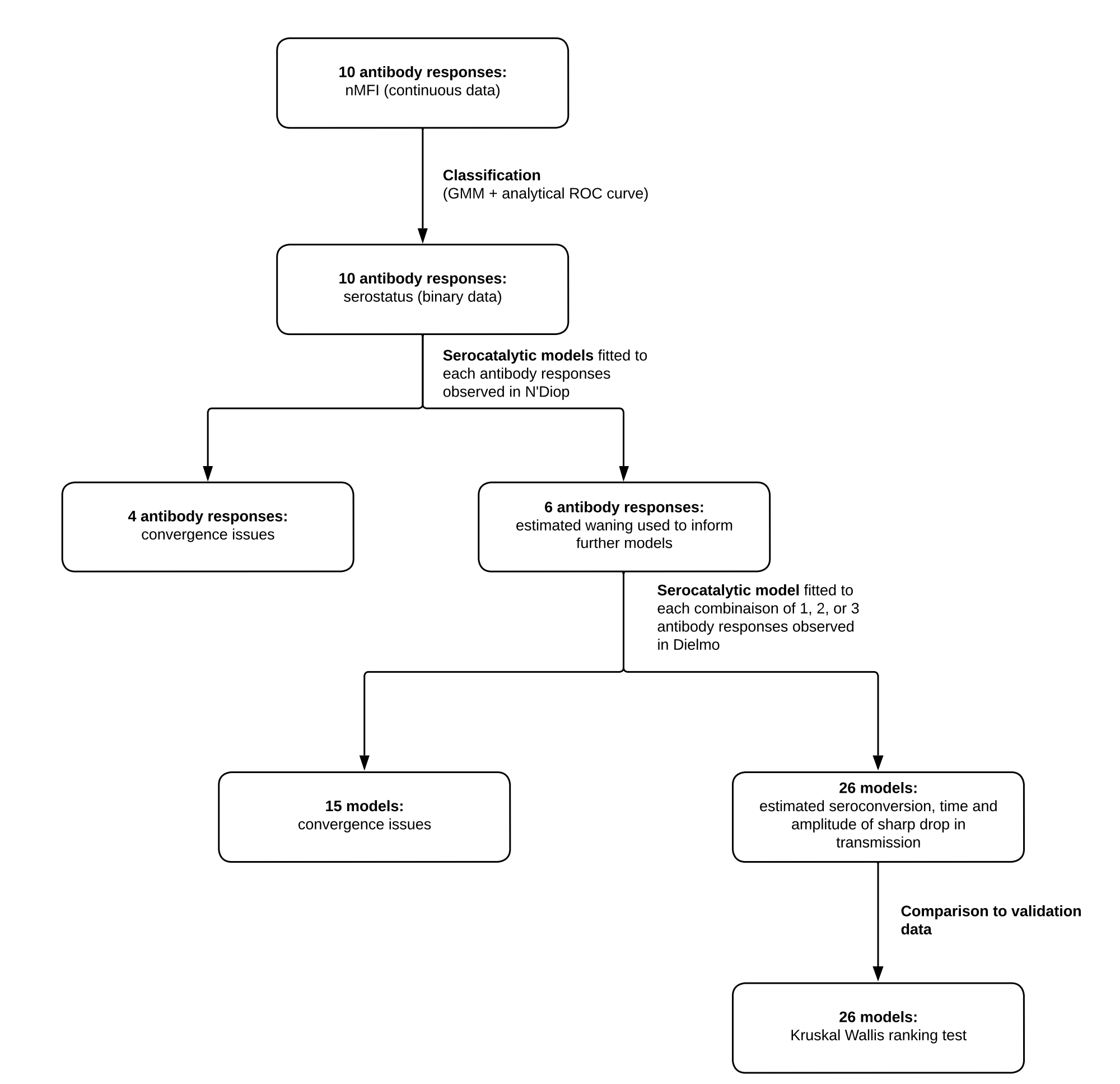


***Figure M: Protocol flowchart.***

- 1. Posterior distributions


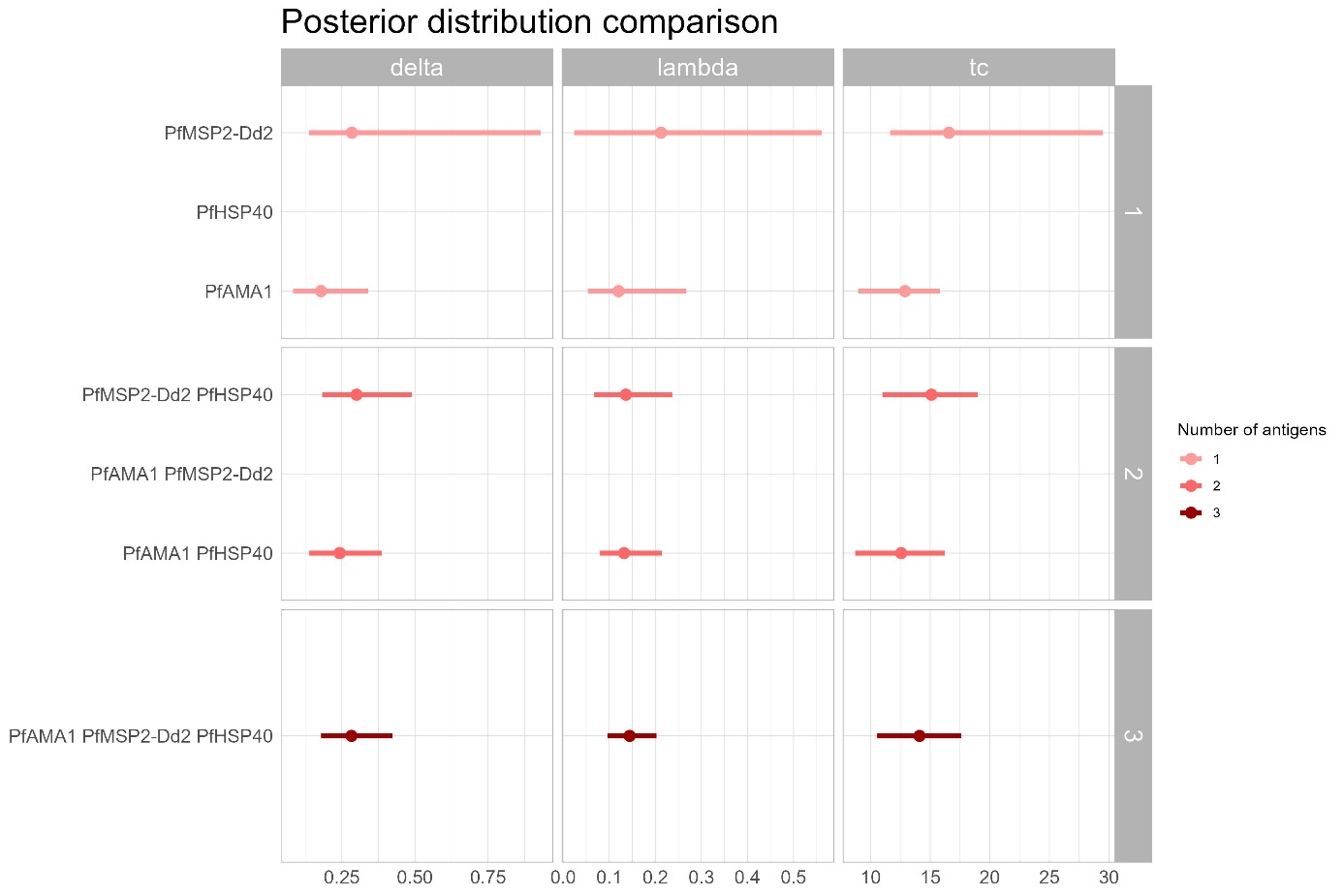


***Figure N: Posterior distribution comparison.*** *Posterior distribution of the three parameters that have been compared to validation data: seroincidence, time and magnitude of sharp drop in transmission. Model presented in this plot are all possible combinations of PfAMA1, PfMSP2-Dd2 and PfHSP40. Only models that converged are presented.*


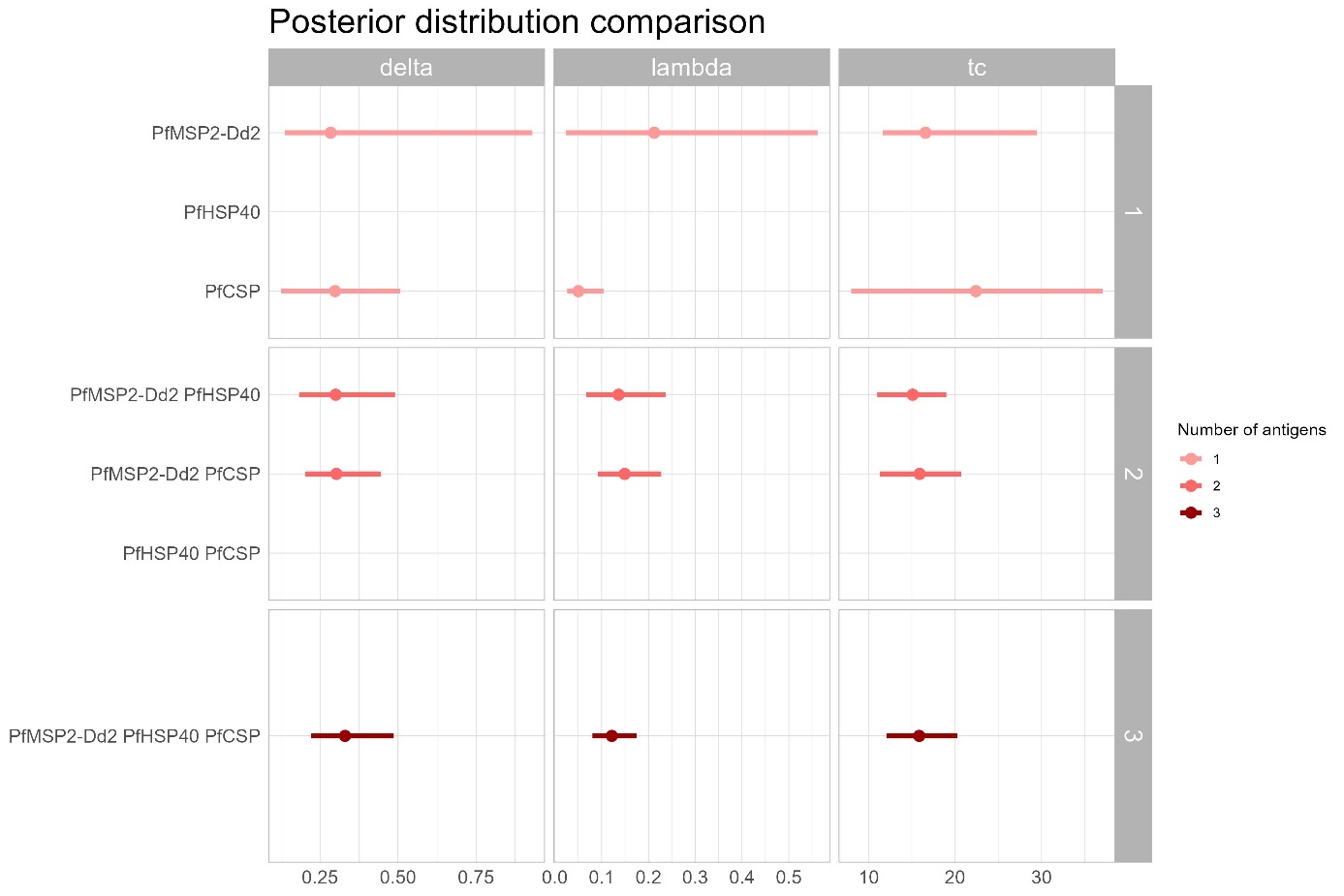


***Figure O: Posterior distribution comparison.*** *Posterior distribution of the three parameters that have been compared to validation data: seroincidence, time and magnitude of sharp drop in transmission. Model presented in this plot are all possible combinations of PfMSP2-Dd2, PfHSP40 and PfCSP. Only models that converged are presented.*


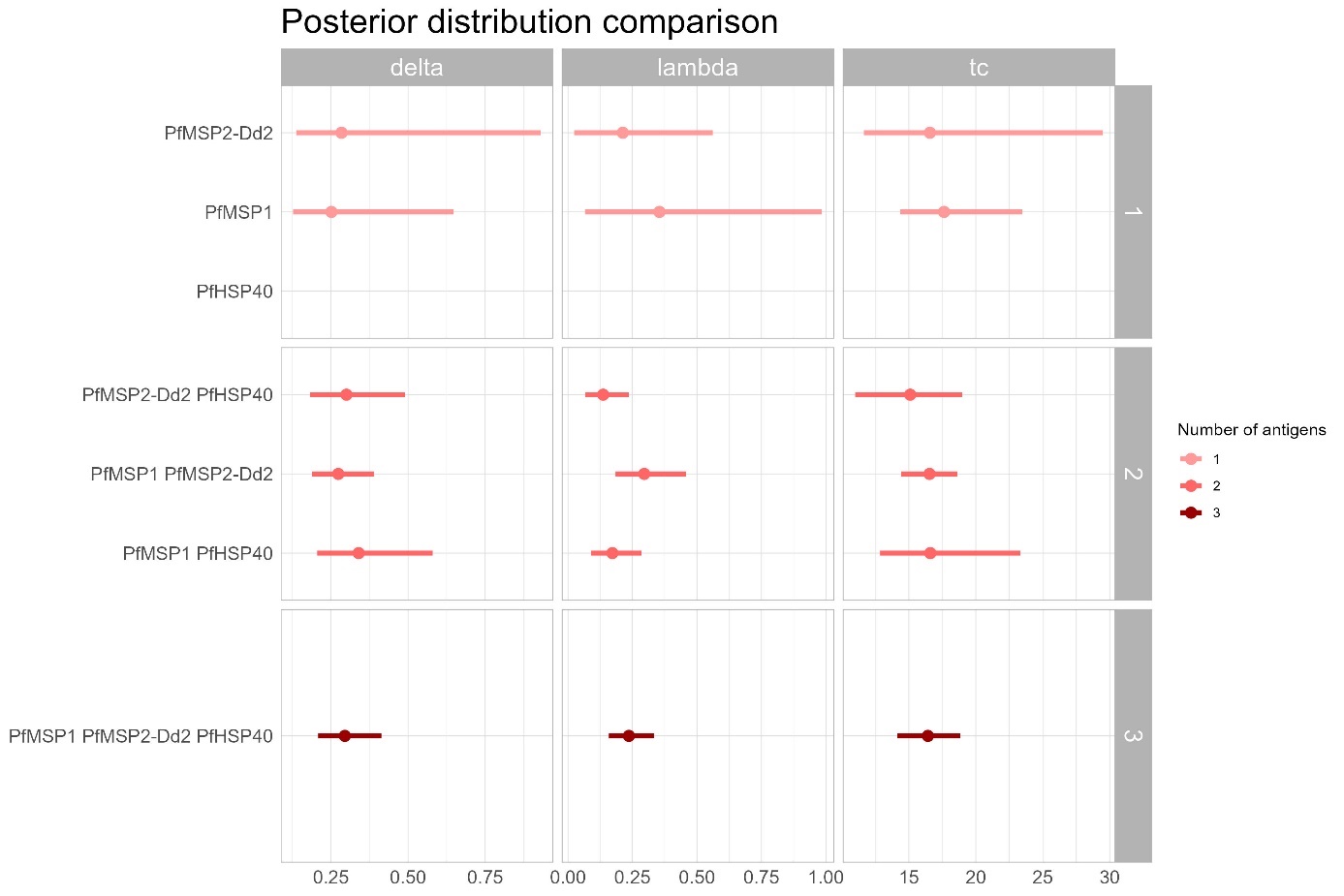


***Figure P: Posterior distribution comparison.*** *Posterior distribution of the three parameters that have been compared to validation data: seroincidence, time and magnitude of sharp drop in transmission. Model presented in this plot are all possible combinations of PfMSP1, PfMSP2-Dd2 and PfHSP40. Only models that converged are presented.*


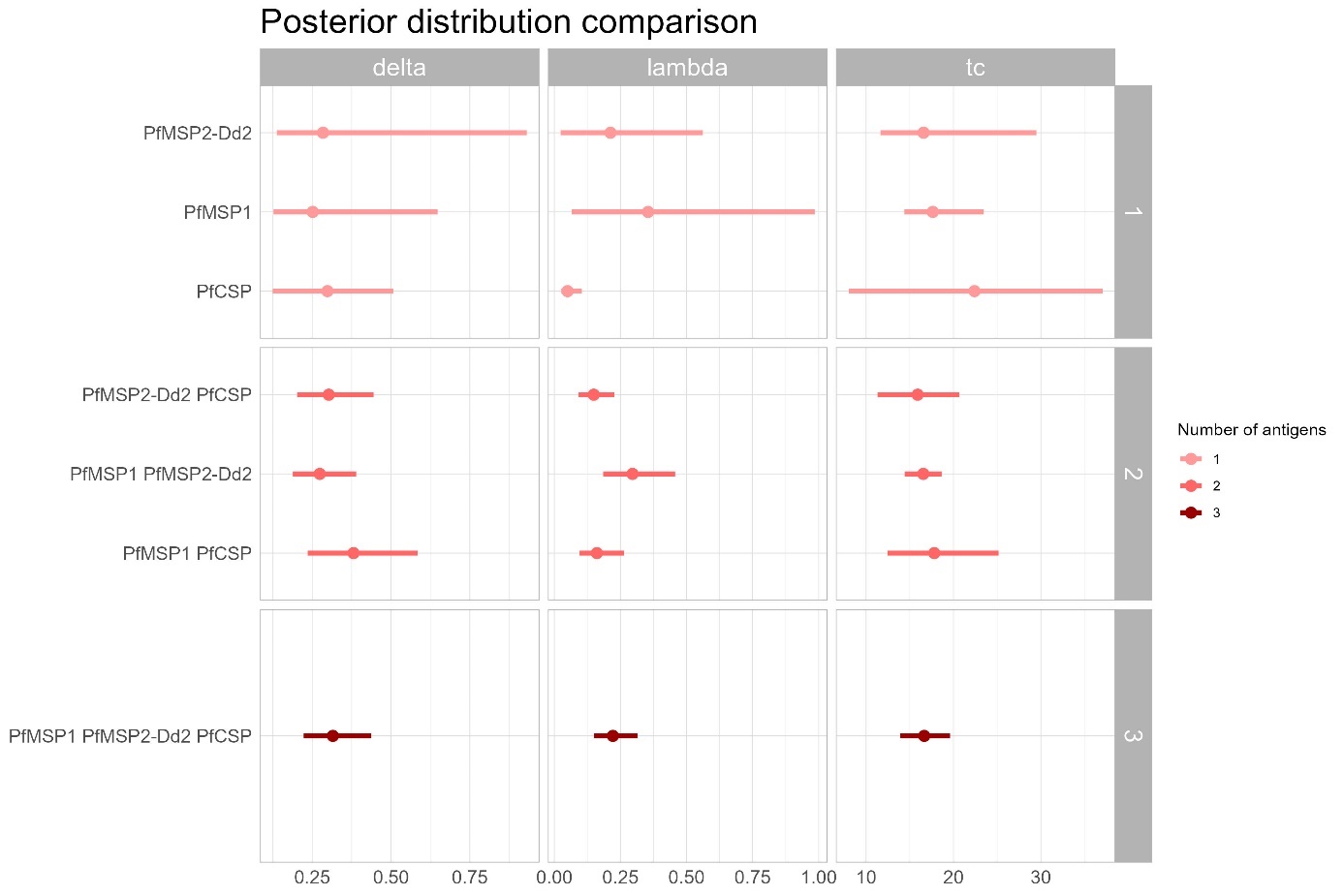


***Figure Q: Posterior distribution comparison.*** *Posterior distribution of the three parameters that have been compared to validation data: seroincidence, time and magnitude of sharp drop in transmission. Model presented in this plot are all possible combinations of PfMSP1, PfMSP2-Dd2 and PfCSP. Only models that converged are presented.*


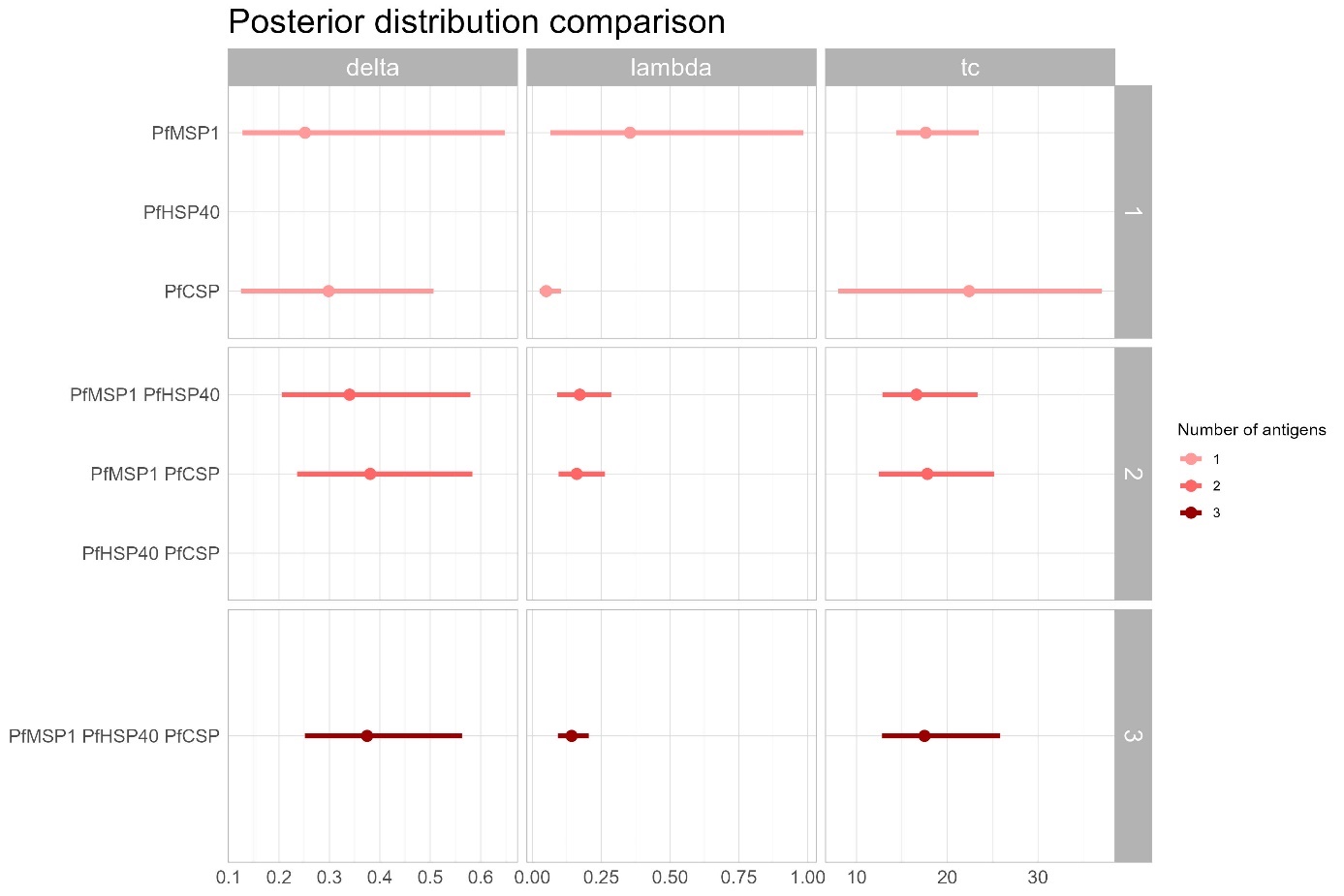


***Figure R: Posterior distribution comparison.*** *Posterior distribution of the three parameters that have been compared to validation data: seroincidence, time and magnitude of sharp drop in transmission. Model presented in this plot are all possible combinations of PfMSP1, PfHSP40 and PfCSP. Only models that converged are presented.*


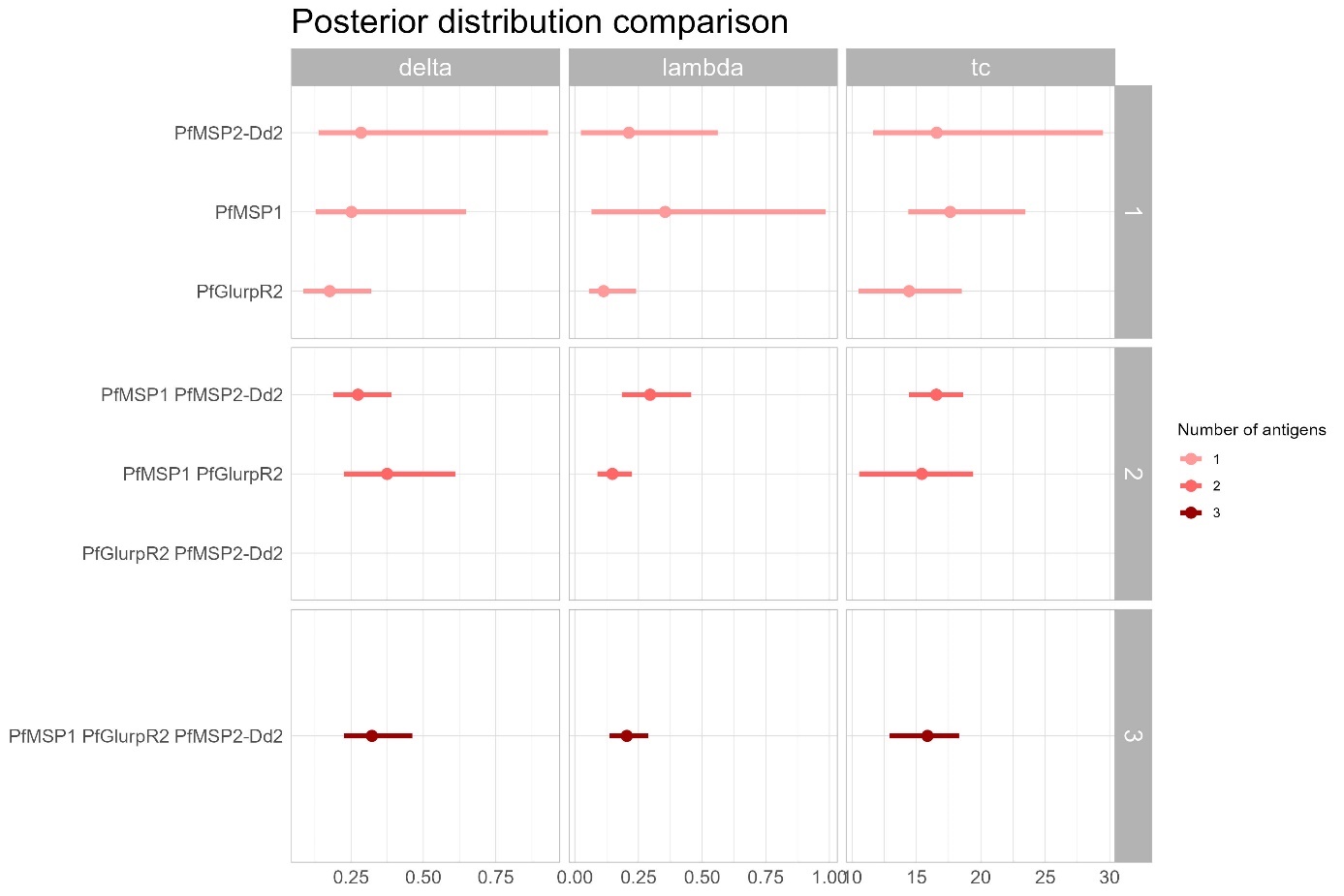


***Figure S: Posterior distribution comparison.*** *Posterior distribution of the three parameters that have been compared to validation data: seroincidence, time and magnitude of sharp drop in transmission. Model presented in this plot are all possible combinations of PfMSP1, PfGlurpR2 and PfMSP2-Dd2. Only models that converged are presented.*


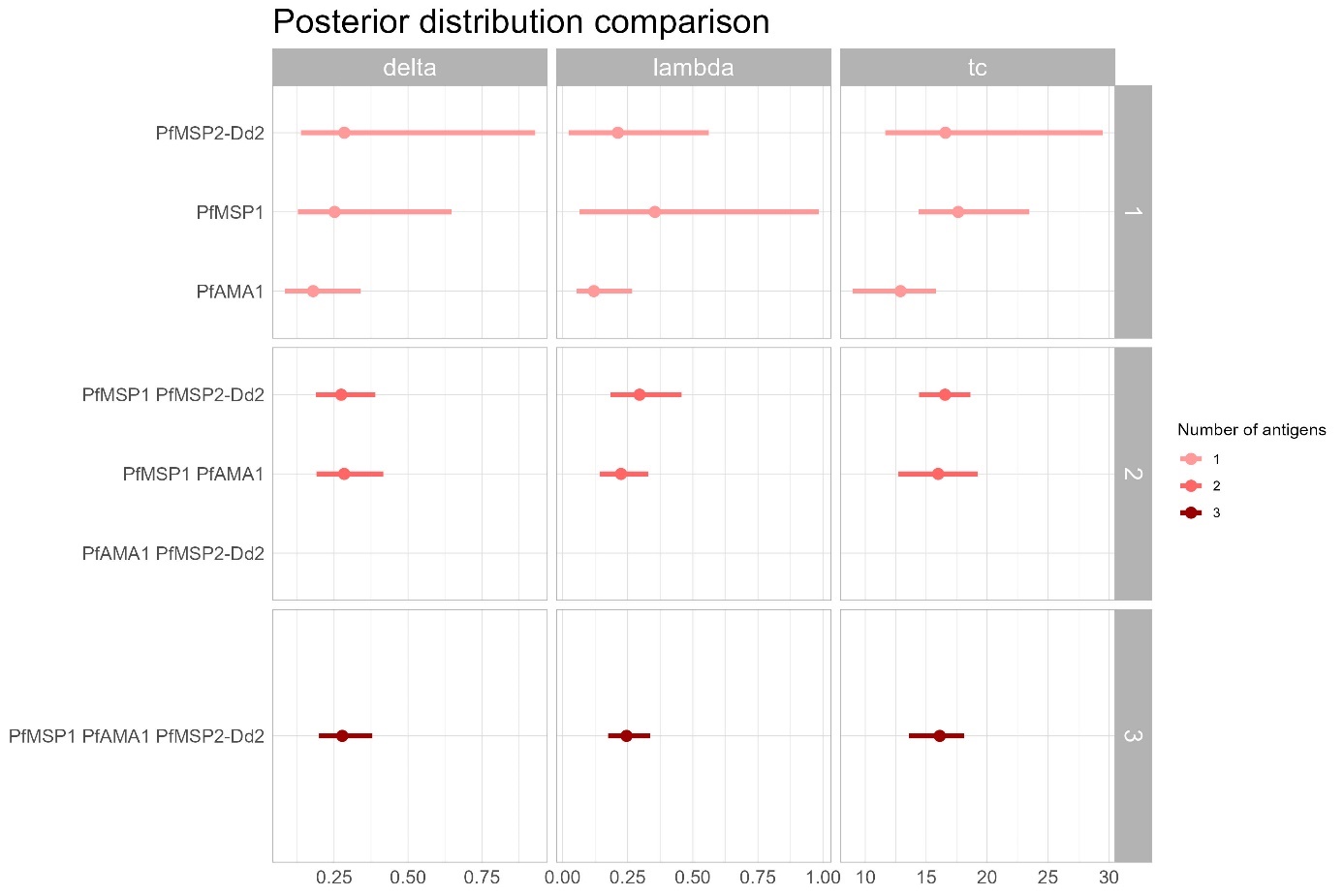


***Figure T: Posterior distribution comparison.*** *Posterior distribution of the three parameters that have been compared to validation data: seroincidence, time and magnitude of sharp drop in transmission. Model presented in this plot are all possible combinations of PfMSP1, PfAMA1 and PfMSP2-Dd2. Only models that converged are presented.*


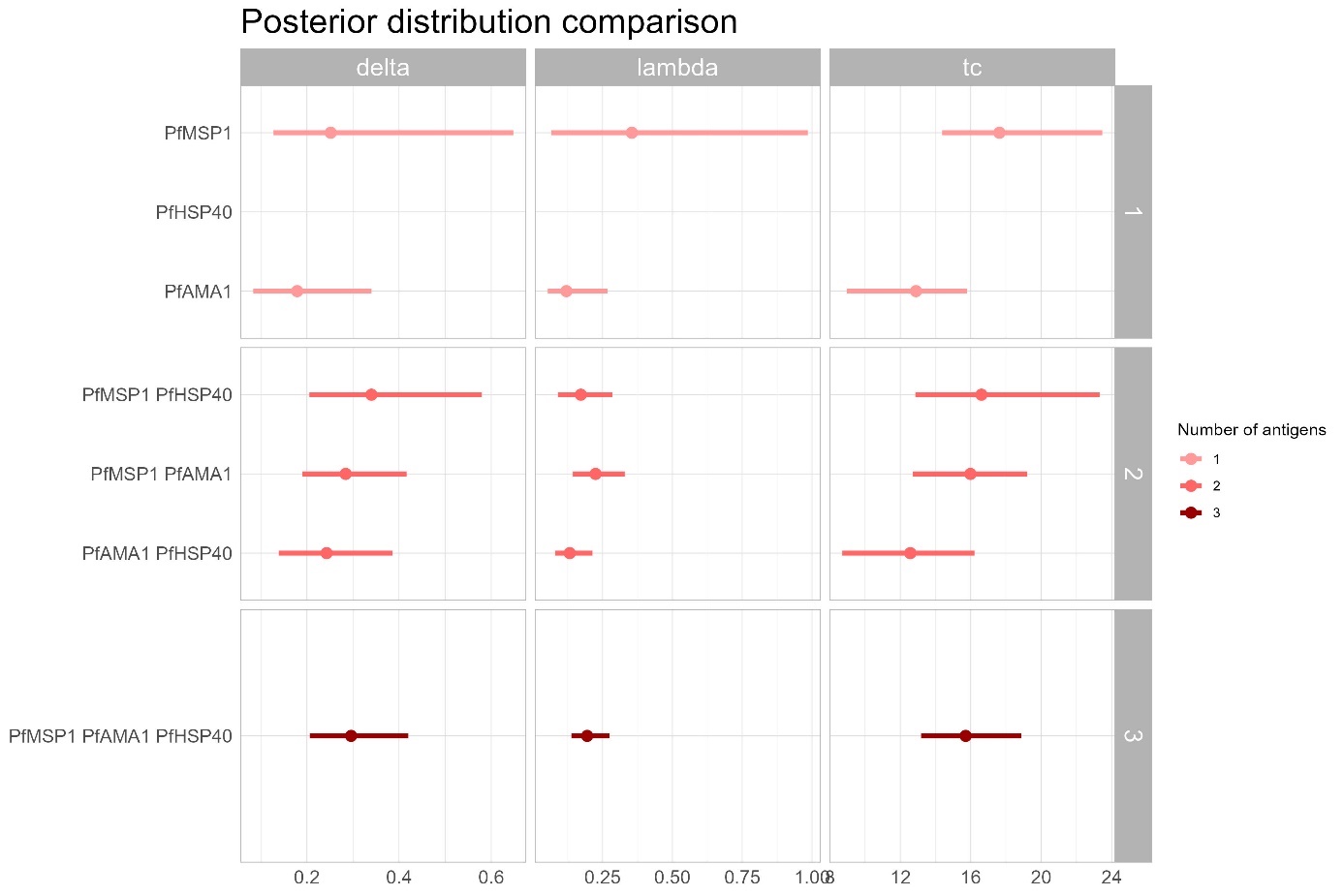


***Figure U: Posterior distribution comparison.*** *Posterior distribution of the three parameters that have been compared to validation data: seroincidence, time and magnitude of sharp drop in transmission. Model presented in this plot are all possible combinations of PfMSP1, PfAMA1 and PfHSP40. Only models that converged are presented.*


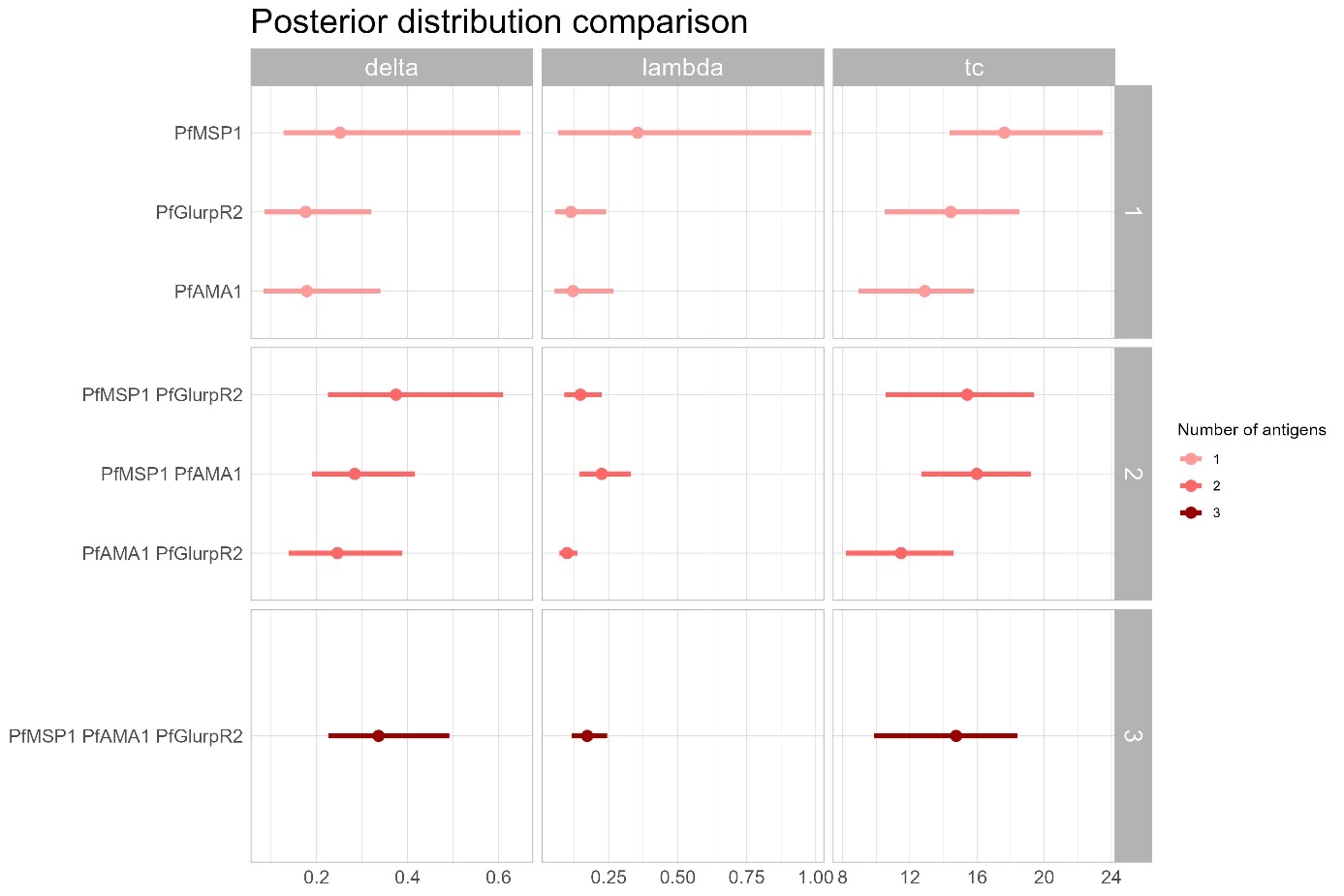


***Figure V: Posterior distribution comparison.*** *Posterior distribution of the three parameters that have been compared to validation data: seroincidence, time and magnitude of sharp drop in transmission. Model presented in this plot are all possible combinations of PfMSP1, PfAMA1 and PfGlurpR2. Only models that converged are presented.*


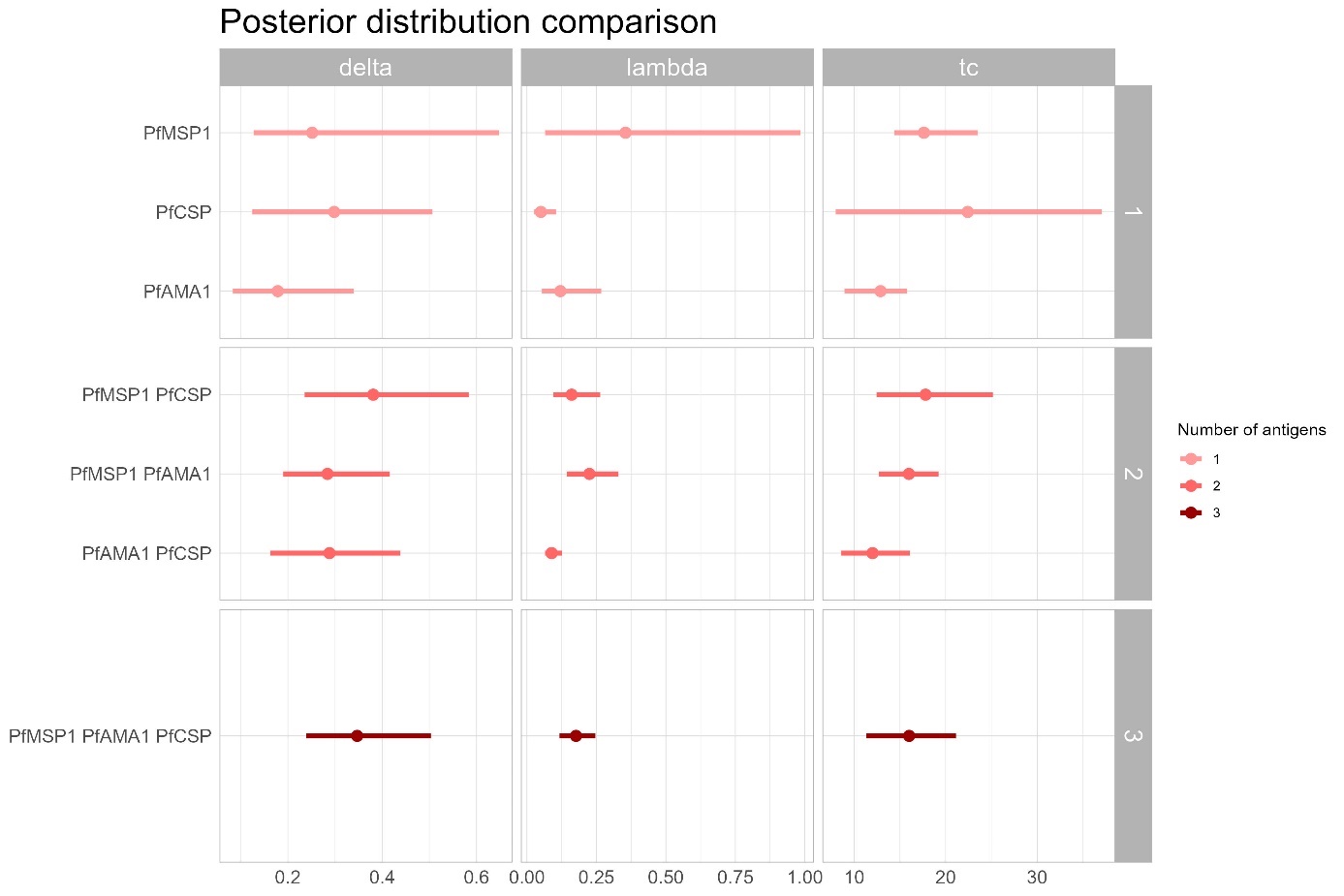


***Figure W: Posterior distribution comparison.*** *Posterior distribution of the three parameters that have been compared to validation data: seroincidence, time and magnitude of sharp drop in transmission. Model presented in this plot are all possible combinations of PfMSP1, PfAMA1 and PfCSP. Only models that converged are presented.*

- 1. Application of the model


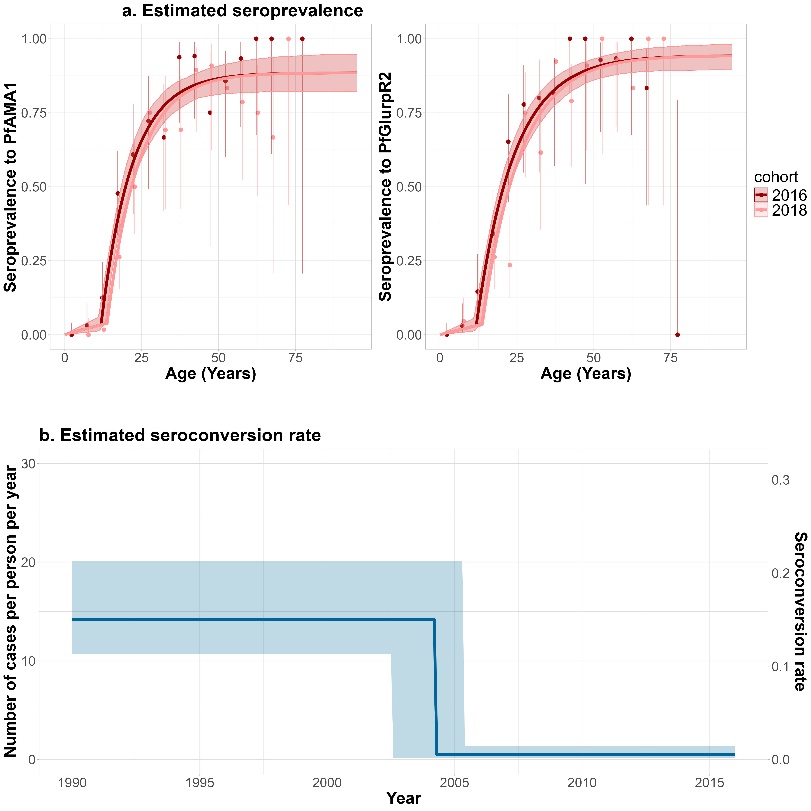


***Figure X: Model comparison to data and seroconversion rate estimation****.* A serocatalytic model including two antigens, PfAMA1 and *Pf*GlurpR2, was fitted to cross-sectional serological data from Ndiop, resulting in an estimated seroconversion rate of 0.15 year^-1^, which dropped to 0.005 year^-1^ 11.8 years ago. *Panel A compares Ndiop’s observed and model-predicted age-stratified seroprevalence, for a multiplex serocatalytic model including both PfAMA1 and PfGlurpR2 and considering one drop in transmission. In Panel B the seroincidence estimated by the same model over 25 years prior to the 2016 cross section is multiplied by the conversion coefficient computed for the same model fitted on Dielmo.*

- 1. Sensitivity analysis on cutoffs

A sensitivity analysis was conducted to assess the impact of choosing different cutoffs. We fitted a multiplex serocatalytic model including *Pf*AMA1 and *Pf*GlurpR2 to serological data from Dielmo dichotomized with cutoffs corresponding to the Youden Index, a specificity (Sp) of 99.9%, and the observed $\mu_{neg}+2{sd}_{neg}$. In the manuscript, the cutoff used corresponded to a Sp = 99%. We compared the posterior distributions of the seroconversion rate $\lambda$, the time at which a drop in transmission occurred $tc$, and its magnitude $\delta$ across the four models to the estimates presented in the manuscript (Figure S25). The estimated seroconversion rates range from 0.1 year^-1^ for the cutoff used in the manuscript to 0.2 year^-1^ for the observed $\mu_{neg}+2{sd}_{neg}$. All models estimated a drop in transmission in 2004 or 2005, 11 or 12 years prior to 2016. The estimated magnitude of this decline is higher for the model presented in the manuscript compared to models fitted to data dichotomized by all other cutoffs. All models were capable of recapturing the data they have been fitted on and matched with the validation data (Figures S26 to S28).

**
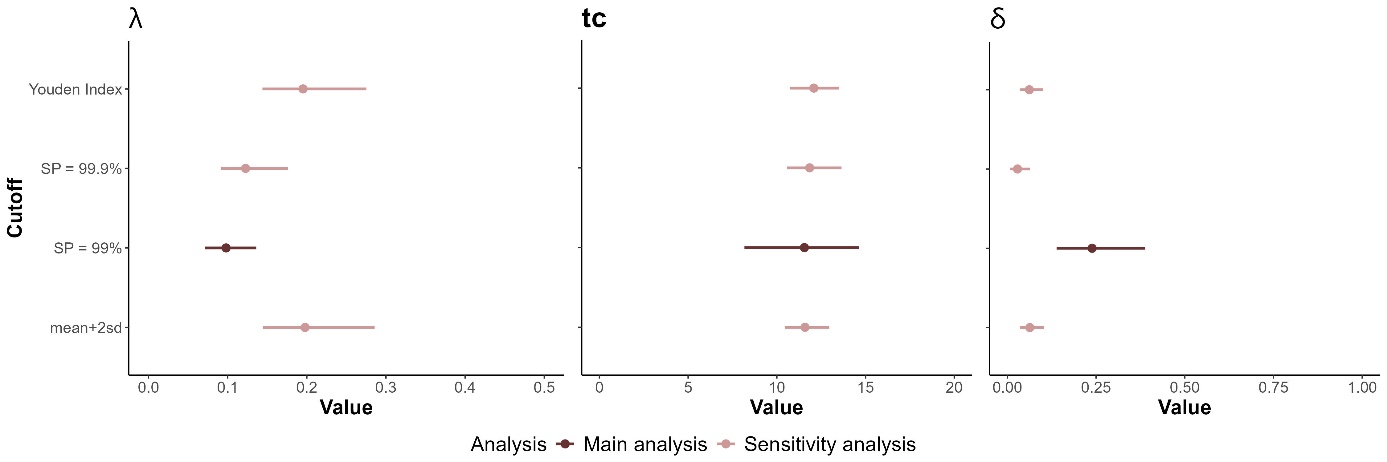
**

***Figure Y: Sensitivity analysis of cutoff on estimated model parameters.*** *Posterior distributions of the seroconversion rate, the time of drop in transmission and its magnitude. Parameters were estimated by a serocatalytic model including PfAMA1 and PfGlurpR2 fitted to the 2016 and 2018 cohorts in Dielmo. For each of the three models, data were binarized using one of the following cutoffs: a cutoff corresponding to the Youden Index, one corresponding to a specificity (Sp) of 99.9%, the observed* $\mu_{neg}+2{sd}_{neg}$*. The cutoff corresponding to a Sp = 99% is the one used in the main analysis presented in the manuscript.*


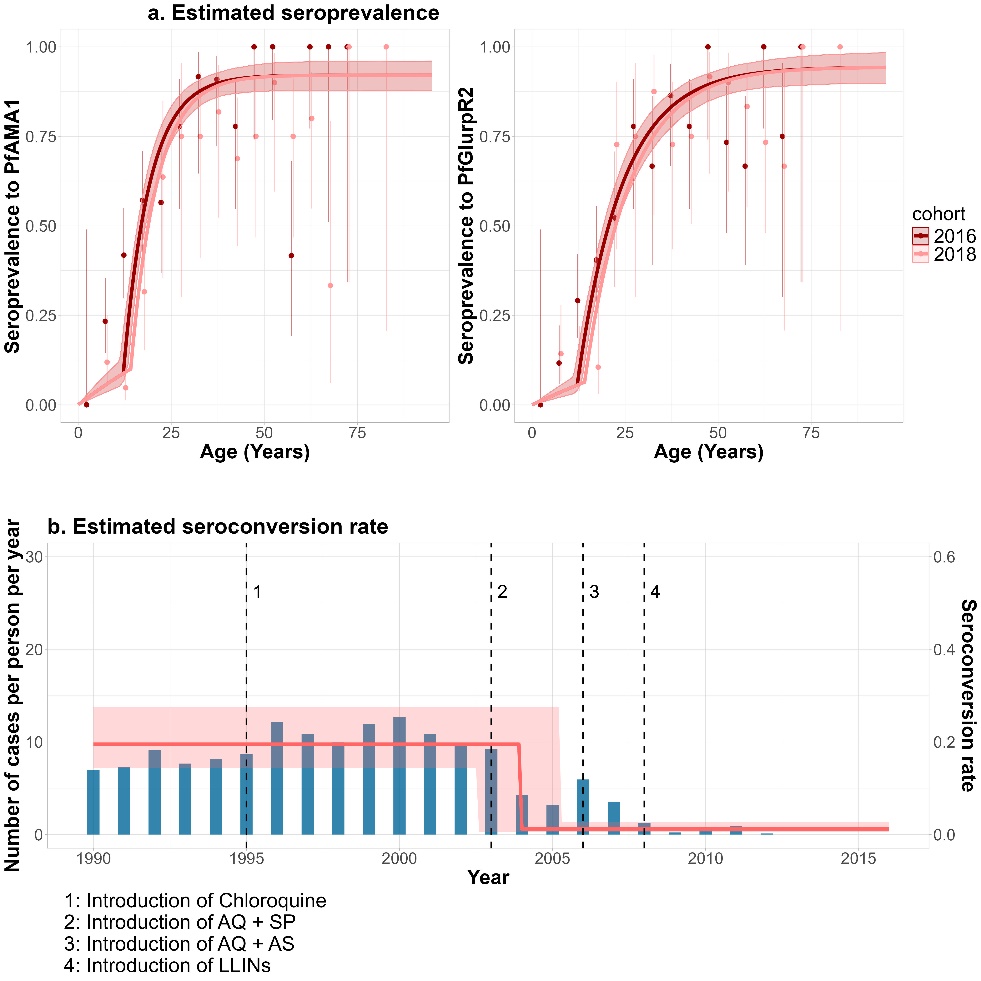


***Figure Z: Model comparison to data and validation using a Youden Index to define the cutoff for seropositivity.*** *Panel A compares Dielmo’s observed and model-predicted age-stratified seroprevalence, for a multiplex serocatalytic model including both PfAMA1 and PfGlurpR2 and considering one drop in transmission. Data were dichotomized using a cutoff corresponding to the Youden Index. In Panel B the seroincidence estimated by the same model over 25 years prior to the 2016 cross section is compared with external validation data on the observed number of cases per person per year.*


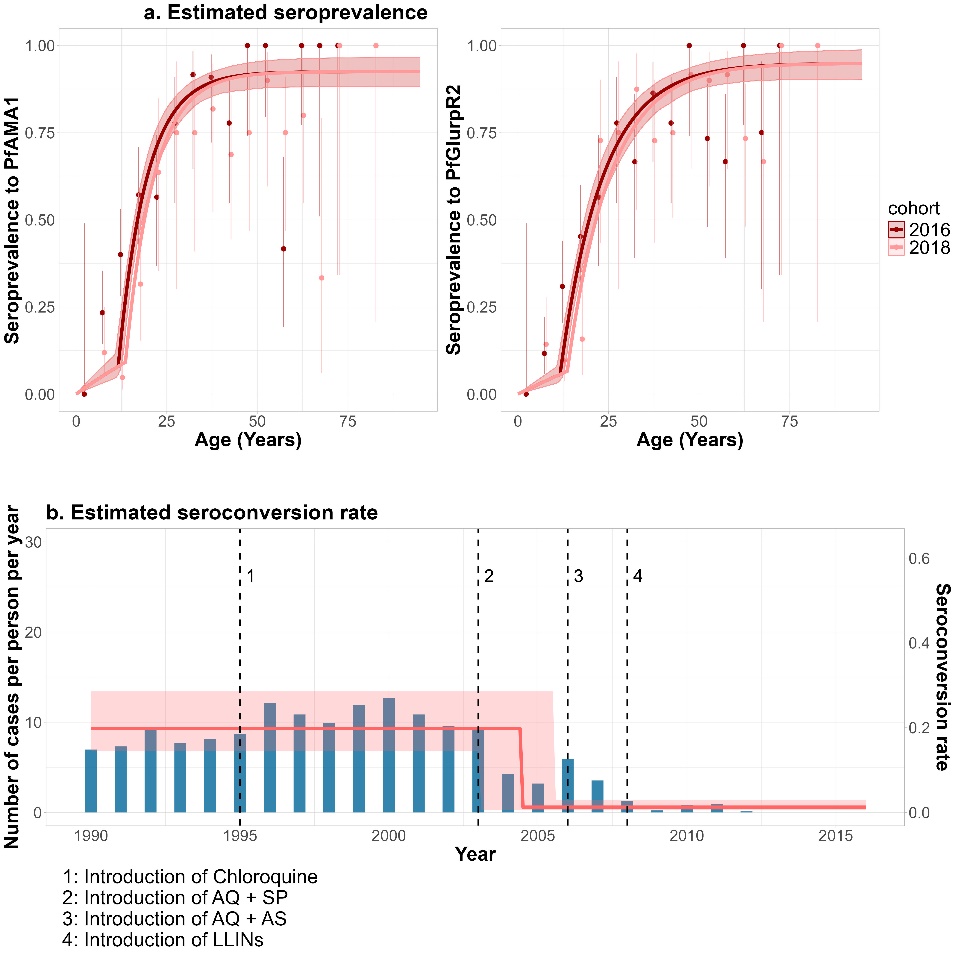


***Figure ZA: Model comparison to data and validation using the*** $\mu_{neg}+2{sd}_{neg}$ ***to define the cutoff for seropositivity.*** *Panel A compares Dielmo’s observed and model-predicted age-stratified seroprevalence, for a multiplex serocatalytic model including both PfAMA1 and PfGlurpR2 and considering one drop in transmission. Data were dichotomized using* $\mu_{neg}+2{sd}_{neg}$ *as the cutoff. In Panel B the seroincidence estimated by the same model over 25 years prior to the 2016 cross section is compared with external validation data on the observed number of cases per person per year.*


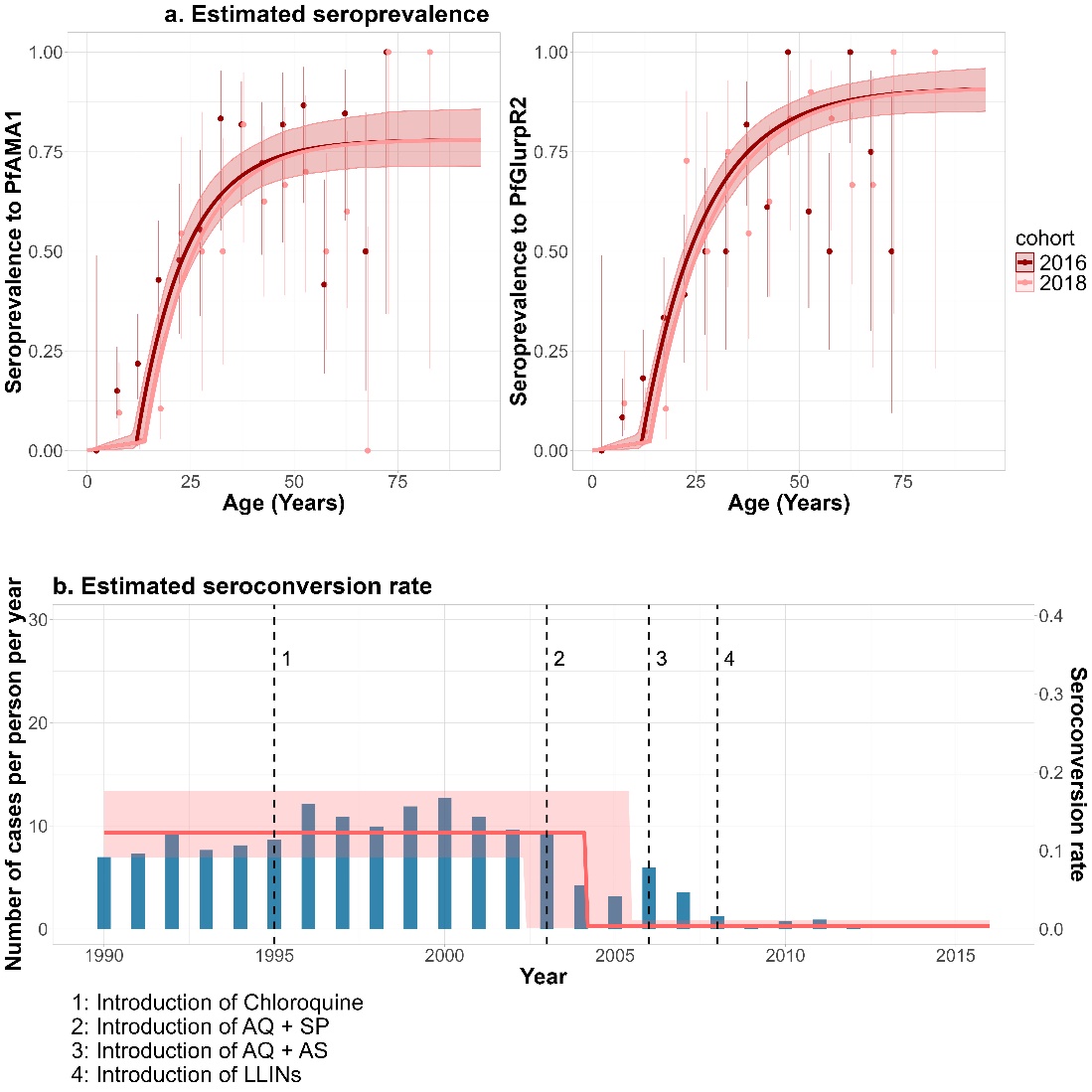


***Figure ZB: Model comparison to data and validation using a cutoff corresponding to a 99.9% specificity to define seropositivity.*** *Panel A compares Dielmo’s observed and model-predicted age-stratified seroprevalence, for a multiplex serocatalytic model including both PfAMA1 and PfGlurpR2 and considering one drop in transmission. Data were dichotomized using a cutoff corresponding to a specificity of 99.9%. In Panel B the seroincidence estimated by the same model over 25 years prior to the 2016 cross section is compared with external validation data on the observed number of cases per person per year.*

- 1. Sensitivity analysis on priors

In the models presented in the manuscript, the prior on the seroreversion rate $\rho$ for a given antigen was defined as followes: $\rho_{antigen}\mathcal{\sim N(}\mu_{antigen}^{NDiop}, \sigma_{antigen}^{NDiop})$, with $\mu_{antigen}^{NDiop}$ and $\sigma_{antigen}^{NDiop}$ being the mean and standard deviation of the posterior distribution of the seroreversion rate estimated on data from Ndiop. A sensitivity analysis was conducted to assess the impact of different priors. We fitted three multiplexed serocatalytic models including *Pf*AMA1 and *Pf*GlurpR2 to Dielmo serological data. For the first one, wider priors were defined as $\rho_{antigen}\mathcal{\sim N(}\mu_{antigen}^{NDiop}, 3\sigma_{antigen}^{NDiop})$, for the second one $\sigma_{PfGlurpR2}\mathcal{\sim N(}\mu_{PfGlurpR2}^{NDiop},\sigma_{PfGlurpR2}^{NDiop})$ and $\sigma_{PfAMA1} \sim exp(10)$ and for the last $\sigma_{PfAMA1}\mathcal{\sim N(}\mu_{PfAMA1}^{NDiop},\sigma_{PfAMA1}^{NDiop})$ and $\sigma_{PfGlurpR2} \sim exp(10)$.

We compared the posterior distributions of the seroconversion rate $\lambda$, the time at which a sharp drop in transmission occurred $tc$, and its magnitude $\delta$ with the estimations presented in the manuscript (Figure S29). Estimates from the four models were highly similar, with broadly overlapping posterior distributions. All models were capable of recapturing the data they have been fitted on and matched with the validation data (Figures S30 to S32).


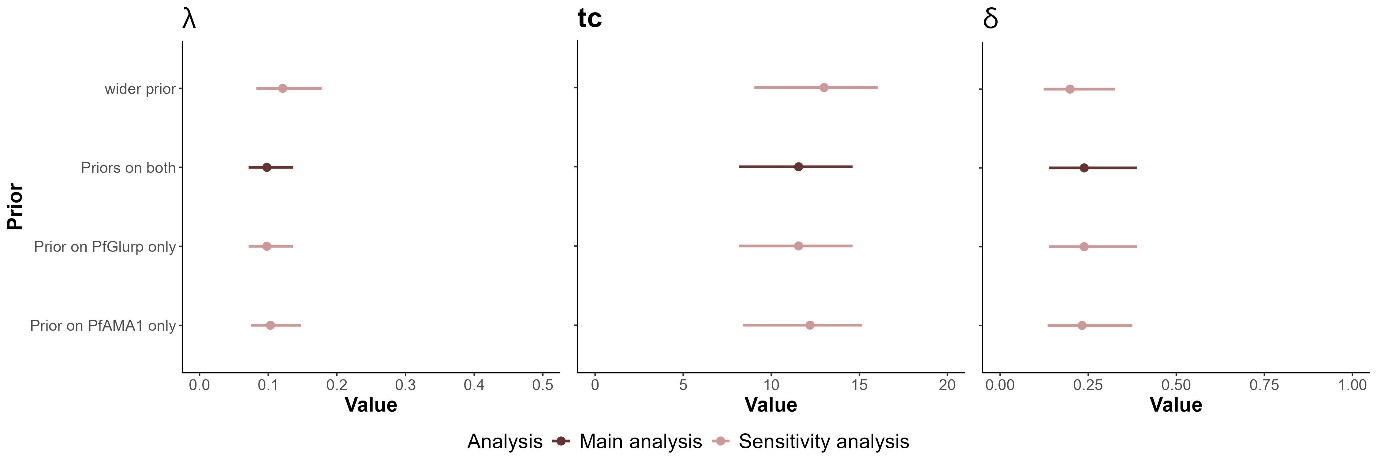


**Figure ZC: Sensitivity analysis of choice of prior distributions.** Posterior distributions of the seroconversion rate, the time of a sharp drop in transmission and its magnitude. Parameters were estimated by a serocatalytic model including *Pf*AMA1 and *Pf*GlurpR2 fitted to the 2016 and 2018 cohorts in Dielmo. Priors differed for each model. For the first one, wider priors were defined as $\rho_{antigen}\mathcal{\sim N(}\mu_{antigen}^{NDiop}, 3\sigma_{antigen}^{NDiop})$, for the second one $\sigma_{PfGlurpR2}\mathcal{\sim N(}\mu_{PfGlurpR2}^{NDiop},\sigma_{PfGlurpR2}^{NDiop})$ and $\sigma_{PfAMA1}$ was uninformative ($\sigma_{PfAMA1} \sim exp(10)$) and for the last $\sigma_{PfAMA1}\mathcal{\sim N(}\mu_{PfAMA1}^{NDiop},\sigma_{PfAMA1}^{NDiop})$ and $\sigma_{PfGlurpR2}$ was uninformative ($\sigma_{PfGlurpR2} \sim exp(10)$). In the model presented in the manuscript priors on both antigens were defined as $\rho_{antigen}\mathcal{\sim N(}\mu_{antigen}^{NDiop}, \sigma_{antigen}^{NDiop})$.


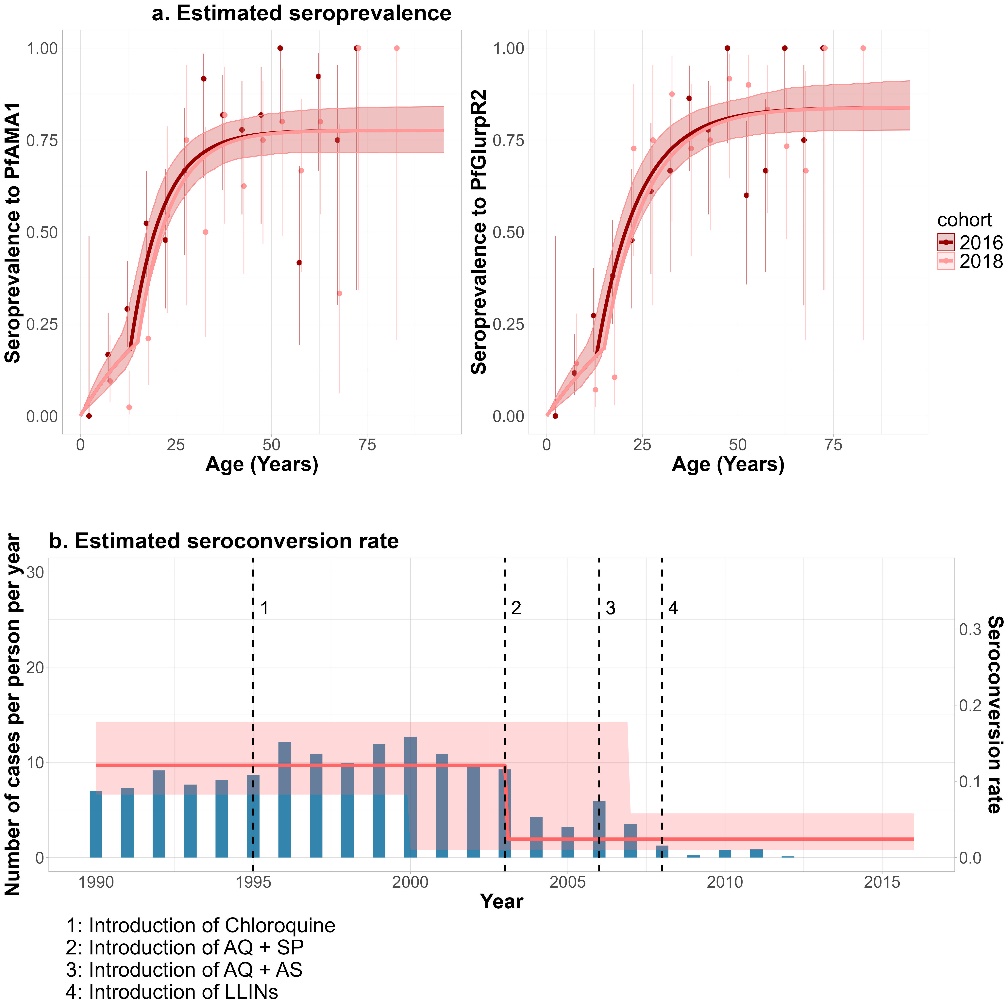


***Figure ZD: Model comparison to data and validation.*** *Priors on the seroconversion rate were defined as* $\rho_{antigen}\mathcal{\sim N(}\mu_{antigen}^{NDiop}, 3\sigma_{antigen}^{NDiop})$*, with* $\mu_{antigen}^{NDiop}$ and $\sigma_{antigen}^{NDiop}$ being the mean and standard deviation of the posterior distribution of the seroreversion rate estimated on data from Ndiop. *Panel A compares Dielmo’s observed and model-predicted age-stratified seroprevalence, for a multiplex serocatalytic model including both PfAMA1 and PfGlurpR2 and considering one drop in transmission. In Panel B the seroincidence estimated by the same model over 25 years prior to the 2016 cross section is compared with external validation data on the observed number of cases per person per year.*


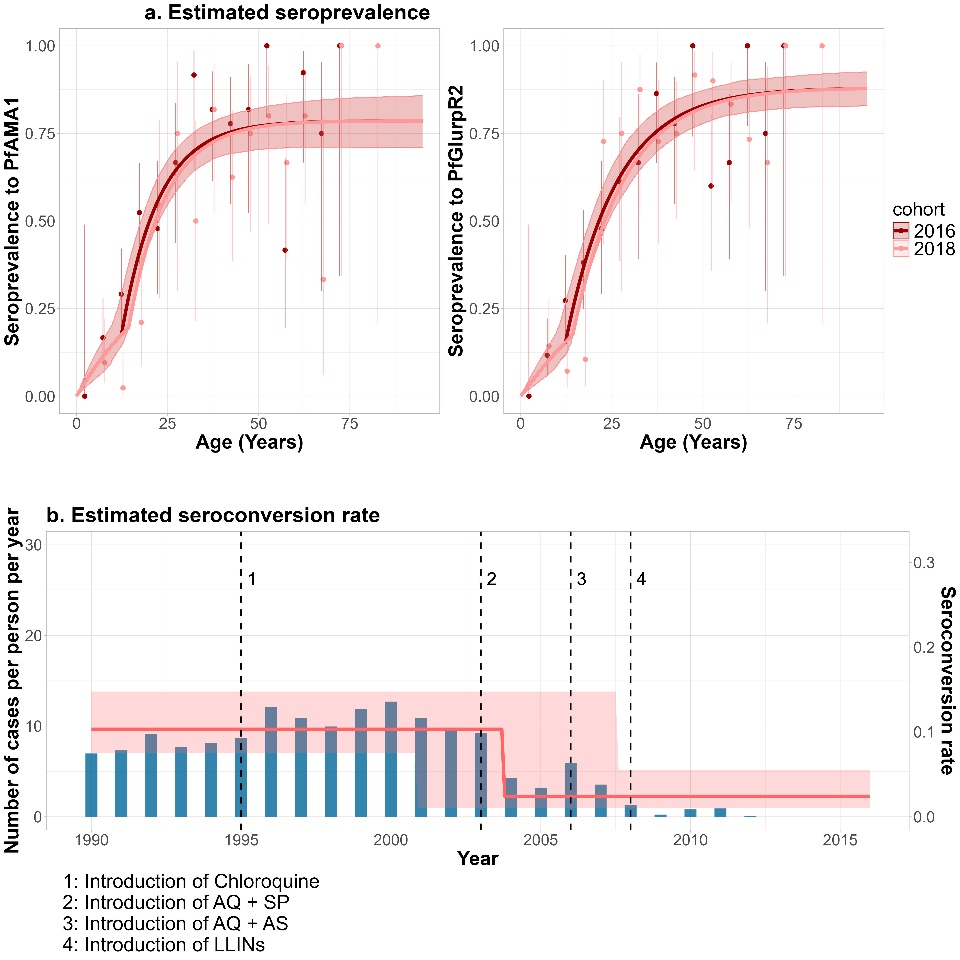


***Figure ZE: Model comparison to data and validation.*** *Priors on the seroconversion rate were defined as* $\sigma_{PfGlurpR2}\mathcal{\sim N(}\mu_{PfGlurpR2}^{NDiop},\sigma_{PfGlurpR2}^{NDiop})$ and $\sigma_{PfAMA1} \sim\exp\left( 10 \right),$*with* $\mu_{PfGlurpR2}^{NDiop}$ and $\sigma_{PfGlurpR2}^{NDiop}$ being the mean and standard deviation of the posterior distribution of the seroreversion rate estimated on data from Ndiop. *Panel A compares Dielmo’s observed and model-predicted age-stratified seroprevalence, for a multiplex serocatalytic model including both PfAMA1 and PfGlurpR2 and considering one drop in transmission. In Panel B the seroincidence estimated by the same model over 25 years prior to the 2016 cross section is compared with external validation data on the observed number of cases per person per year.*


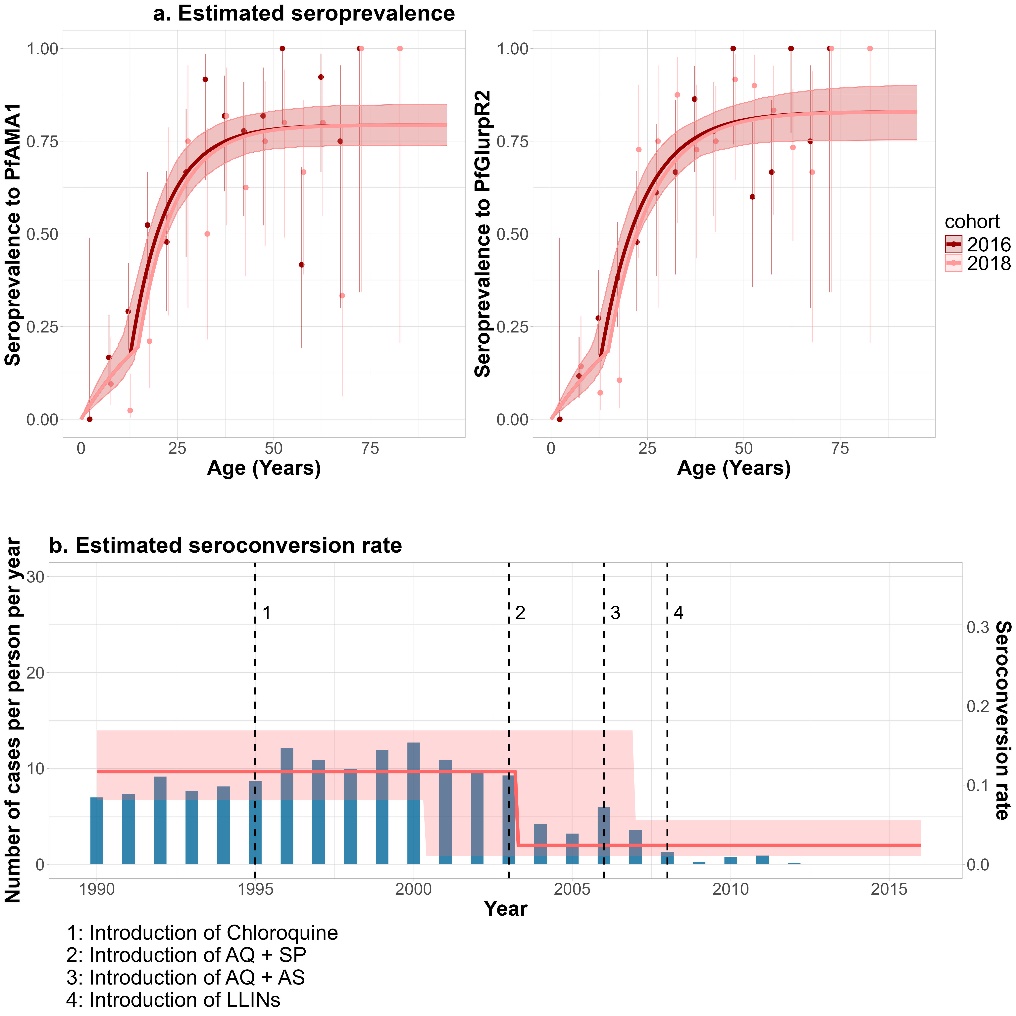


***Figure ZF: Model comparison to data and validation.*** *Priors on the seroconversion rate were defined as* $\sigma_{PfGlurpR2}\mathcal{\sim N(}\mu_{PfAMA1}^{NDiop},\sigma_{PfAMA1}^{NDiop})$ and $\sigma_{PfGlurpR2} \sim\exp\left( 10 \right),$*with* $\mu_{PfAMA1}^{NDiop}$ and $\sigma_{PfAMA1}^{NDiop}$ being the mean and standard deviation of the posterior distribution of the seroreversion rate estimated on data from Ndiop. *Panel A compares Dielmo’s observed and model-predicted age-stratified seroprevalence, for a multiplex serocatalytic model including both PfAMA1 and PfGlurpR2 and considering one drop in transmission. In Panel B the seroincidence estimated by the same model over 25 years prior to the 2016 cross section is compared with external validation data on the observed number of cases per person per year.*
