# Supplementary material for: Reconstruction of historical malaria transmission in Senegal using multiplex serocatalytic models
Source: PLoS Comput Biol. 2026 Jul 27;22(7):e1013630. doi: 10.1371/journal.pcbi.1013630 (PMC13423172; doi:10.1371/journal.pcbi.1013630)
Supplement: Attachment — Submitted filename: Response to Reviewers.docx [file pcbi.1013630.s005.docx]

**Infectious Disease Epidemiology
& Analytics G5 Unit**

Institut Pasteur

25-28 Rue du Dr Roux

Paris 75015

France

**Dr Michael White**

michael.white@pasteur.fr

7^th^ May 2026

Dear Editor,

We would like to resubmit a revised version of our manuscript, “Reconstruction of historical malaria transmission in Senegal using multiplex serocatalytic models”, for consideration for publication in *PLOS Computational Biology*.

We would like to thank the editors and reviewers for their attention to this manuscript. All of the reviewers’ comments have been addressed on a point-by-point basis below. Please note that where line numbers have been noted, this refers to the manuscript with Track Changes with the ‘Simple Mark-up’ option.

We believe that the changes made in response to these suggestions have led to a substantially improved manuscript.

We look forward to your response.

Yours sincerely,

Dr Michael White, on behalf of the authors

**Reviewer #1**

Baudemont et al. developed a serocatalytic model that allows historical malaria endemicity to be estimated from antibody titers measured in a cross-sectional survey. It is an interesting attempt, and the model generation process is reasonable. However, it is questionable for the utility of this model in future studies, and two of the main assumptions used in this study may or may not be appropriate. It would be ideal if the authors could conduct additional analysis to answer the questions, but if not, the points should be discussed as additional limitations of the study.

Thank you to this reviewer for their thorough assessment of our work. In the detailed response below, we highlight the validity and limitations of our assumptions and conduct additional analysis in response to the reviewer’s points.

**Major comments**

1) One change (reduction) in transmission

While the authors had the clinical record in the study sites for a long time, the application of the model is when we don't have such historical data (if we have the data, there is no need to speculate prior endemicity from serological data). A study site could experience multiple reductions, increases (such as seen during the COVID-19 pandemic era), and a combination of both. Therefore, the utility of the model, which assumes there was one sharp drop in transmission in the past, is questionable. How applicable is the model described in this paper when there is no historical data?

The availability of historical data on malaria transmission from the village of Dielmo is a strength of this study, as it allows predictions from models fitted to cross-sectional data to be evaluated. Having demonstrated the utility of the approach we can then apply it to settings where we do not have historical data. This was done by fitting the model to serological data from Ndiop, where we did not have access to historical clinical records. The results of this are summarized in Supplementary Figure S24 (page 23 – 24). Also reproduced below.

***Figure S24:*** ***Model comparison to data and seroconversion rate estimation****.* A serocatalytic model including two antigens, PfAMA1 and PfGlurpR2, was fitted to cross-sectional serological data from Ndiop, resulting in an estimated seroconversion rate of 0.15 year^-1^, which dropped to 0.005 year^-1^ 11.8 years ago. *Panel A compares* Ndiop*’s observed and model-predicted age-stratified seroprevalence, for a multiplex serocatalytic model including both PfAMA1 and PfGlurpR2 and considering one drop in transmission. In Panel B the seroincidence estimated by the same model over 25 years prior to the 2016 cross section is multiplied by the conversion coefficient computed for the same model fitted on Dielmo.*

Adding multiple reductions, increases in transmission, or a combination of both to the model is feasible but identifiability will depend on the information contained in the data. This has been added as a limitation in the discussion following the suggestion of reviewer 1 (lines 297 – 299).

“Models estimating multiple changes in transmission are theoretically possible, but a practical limitation is that these models do not tend to be identifiable for more than two changes in transmission.”

2) Seasonality
   While malaria transmission in Dielmo is perennial, Ndiop is not. Therefore, the sample collection time (e.g., middle of the dry season, end of the transmission season) should be specified. Although it depends on the endemicity, in general, antibody titers are higher at the peak of malaria season compared to those in the middle of the dry season. What is the rationale for ignoring the seasonal effect?

As suggested by Reviewer 1, we added the collection time to Table 1 and the main text (lines 372 – 373). The limitation caused by seasonality is noted in the introduction (lines 118 – 119).

“In both villages, sampling was performed at the end of the dry season from May to June 2016, and then at the beginning of the dry season in December 2018.”

“Serocatalytic models account for long term trends of cumulative exposure and tend to smooth out seasonal effects.”

3) Constant seroconversion rate (ramda)

Based on Figure 3a (and also seen in many sero-epidemiology studies), the sero-prevalence generally increases rapidly <~20-30 years old, but it reaches a plateau after ~30 years old. What is the rationale for the constant seroconversion rate?

In response to this suggestion, we provide a more detailed description of the properties of serocatalytic models (lines 426 – 428):

“Under this model, seroprevalence increases rapidly with age among younger participants before reaching an equilibrium prevalence of $\frac{\lambda}{\lambda+\rho}$.”

In this study, we also allow for non-constant seroconversion by including a potential drop in the seroconversion rate in an otherwise constant transmission setting.

**Minor comments**

- Line 112: up to 500 antibody responses

Dr. Tsuboi and his group have published several papers where they used >1800 antigens (e.g., PMID28378857).

Thank you for bringing this article to our attention. It has now been cited and the text updated (lines 104 – 106).

“Technologies such as multiplex bead-based assays allow for simultaneous quantification of hundreds or thousands of antibody responses from a few microliters of plasma ^12,13^”

- parasite strain, and amino acid positions for the recombinant proteins

Except for PfMSP2-Dd2 and PfMSP2-CH150, the strain of parasite (3D7?) that was selected was not specified. In addition, readers cannot tell whether full-length or truncated (e.g., amino acid XX to XX) proteins were made. That information should be written to replicate the study.

The information on parasite strain and amino acid positions for these recombinant proteins has been previously reported by our team. This has been noted in the methods section (lines 342 – 344).

“Additional information on these antigens’ characteristics including production system and sequence information is reported in van den Hoogen et al^29^ and Wu et al^12^.”

- Line 202: "two long-lived blood stage antigens".

What does it mean by "two long-lived blood stage antigens"? Since the study was conducted in highly endemic areas, we cannot tell whether a single infection induced long-lived antibody responses, continuous (asymptomatic) infections maintained high titers, or a mixture of both. In addition, based on Figure 1, the positive rate of PfMSP1 was also high as PfAMA1 or PfGlurpR2.

Reviewer 1 is correct, in this setting we cannot infer whether antibody responses are long- or short-lived. This sentence is referring to prior knowledge on *Pf*AMA1 and *Pf*GlurpR2 described in <https://doi.org/10.1186/s12916-020-01724-5>. The citation has been added (line 199).

- Figure 1 legend

Since figures and tables should stand by themselves, please explain the abbreviations of antigen names in the legend for Figure 1.

As suggested, the abbreviations of antigens have been explained in the legend of Figure 1 (line 154 – 162).

- Is there any difference in sero-positivity depending on the year or location of sample collection? While it is nice to present the individual data in Figure 1, there are too many points to judge whether there is any difference between 2016 and 2018, and/or between Ndiop and Dielmo. Please add the information in the main text, and if there a significant difference (s), please discuss whether the difference affects any model building or interpretation.

A paragraph on differences in seropositivity was added to the discussion (lines 148 – 150) and a table describing the location-, cohort- and age-stratified seropositivity was added to the supplementary files (Table S2 and S3, pages 2 – 4):

“For antibodies to all the studied antigens, seroprevalence was higher in Dielmo than in Ndiop, and higher in 2016 than in 2018, with the majority of differences being statistically significant (Table S2 and S3)”.

| **Seroprevalence (%)** | | | |
| --- | --- | --- | --- |
|  | **Dielmo** | **Ndiop** | **p-value** |
| ***PfCSP*** | **37** | **23** | **2.8e-07 ***** |
| ***5–10-year-old*** | 7 | 6 | 0.9 |
| ***10–20-year-old*** | 17 | 6 | 0.002 ** |
| ***20+ year-old*** | 59 | 48 | 0.008 ** |
| ***PfGlurpR2*** | **47** | **37** | **0.0007 ***** |
| ***5–10-year-old*** | 10 | 3 | 0.06 |
| ***10–20-year-old*** | 22 | 16 | 0.2 |
| ***20+ year-old*** | 75 | 75 | 1 |
| ***PfAMA1*** | **47** | **37** | **0.001 **** |
| ***5–10-year-old*** | 10 | 2 | 0.01 * |
| ***10–20-year-old*** | 26 | 16 | 0.02 * |
| ***20+ year-old*** | 72 | 76 | 0.3 |
| ***PfMSP1*** | **54** | **54** | **1** |
| ***5–10-year-old*** | 28 | 37 | 0.2 |
| ***10–20-year-old*** | 36 | 46 | 0.06 |
| ***20+ year-old*** | 75 | 78 | 0.4 |
| ***PfEtramp4*** | **27** | **13** | **1.1e-09 ***** |
| ***5–10-year-old*** | 14 | 4 | 0.02 * |
| ***10–20-year-old*** | 16 | 8 | 0.03 * |
| ***20+ year-old*** | 39 | 24 | 0.0002 *** |
| ***PfHSP40*** | **17** | **13** | **0.07** |
| ***5–10-year-old*** | 5 | 4 | 1 |
| ***10–20-year-old*** | 9 | 6 | 0.2 |
| ***20+ year-old*** | 26 | 26 | 1 |
| ***PfMSP2-Dd2*** | **32** | **21** | **1.8e-05 ***** |
| ***5–10-year-old*** | 15 | 3 | 0.004 ** |
| ***10–20-year-old*** | 17 | 8 | 0.01 * |
| ***20+ year-old*** | 47 | 42 | 0.3 |
| ***PfMSP2-CH150*** | **3** | **0.6** | **0.0008 ***** |
| ***5–10-year-old*** | 3 | 0 | 0.1 |
| ***10–20-year-old*** | 2 | 1 | 0.9 |
| ***20+ year-old*** | 4 | 0.7 | 0.01 * |
| ***PfSEA1*** | **12** | **10** | **0.5** |
| ***5–10-year-old*** | 10 | 5 | 0.2 |
| ***10–20-year-old*** | 11 | 9 | 0.6 |
| ***20+ year-old*** | 13 | 15 | 0.4 |
| ***PfSBP1*** | **11** | **9** | **0.3** |
| ***5–10-year-old*** | 9 | 4 | 0.2 |
| ***10–20-year-old*** | 11 | 9 | 0.5 |
| ***20+ year-old*** | 12 | 13 | 0.7 |

***Table S2***. **Overall and age stratified observed seroprevalence by village**. Group differences were assessed using $\mathcal{X}^{2}$ tests using the *chisq.test* function in R and p-values are reported in the table.

| **Seroprevalence (%)** | | | |
| --- | --- | --- | --- |
|  | **2016** | **2018** | **p-value** |
| ***PfCSP*** | **30** | **27** | **0.3** |
| ***5–10-year-old*** | 7 | 5 | 0.9 |
| ***10–20-year-old*** | 15 | 7 | 0.02 * |
| ***20+ year-old*** | 57 | 49 | 0.08 |
| ***PfGlurpR2*** | **41** | **41** | **1** |
| ***5–10-year-old*** | 8 | 4 | 0.5 |
| ***10–20-year-old*** | 24 | 12 | 0.004 ** |
| ***20+ year-old*** | 76 | 74 | 0.7 |
| ***PfAMA1*** | **43** | **38** | **0.1** |
| ***5–10-year-old*** | 8 | 1 | 0.04 * |
| ***10–20-year-old*** | 29 | 10 | 1.2e-05 *** |
| ***20+ year-old*** | 77 | 71 | 0.1 |
| ***PfMSP1*** | **56** | **52** | **0.2** |
| ***5–10-year-old*** | 35 | 31 | 0.6 |
| ***10–20-year-old*** | 46 | 36 | 0.05 |
| ***20+ year-old*** | 82 | 71 | 0.003 ** |
| ***PfEtramp4*** | **21** | **17** | **0.2** |
| ***5–10-year-old*** | 8 | 9 | 1 |
| ***10–20-year-old*** | 13 | 10 | 0.4 |
| ***20+ year-old*** | 36 | 25 | 0.01 * |
| ***PfHSP40*** | **17** | **12** | **0.02 *** |
| ***5–10-year-old*** | 6 | 2 | 0.3 |
| ***10–20-year-old*** | 10 | 4 | 0.04 * |
| ***20+ year-old*** | 30 | 21 | 0.03 * |
| ***PfMSP2-Dd2*** | **28** | **23** | **0.03 *** |
| ***5–10-year-old*** | 12 | 3 | 0.04 * |
| ***10–20-year-old*** | 16 | 7 | 0.01 * |
| ***20+ year-old*** | 49 | 40 | 0.04 * |
| ***PfMSP2-CH150*** | **3** | **0.4** | **0.004 **** |
| ***5–10-year-old*** | 3 | 0 | 0.3 |
| ***10–20-year-old*** | 2 | 0.6 | 0.4 |
| ***20+ year-old*** | 4 | 0.4 | 0.01 * |
| ***PfSEA1*** | **11** | **10** | **0.6** |
| ***5–10-year-old*** | 6 | 9 | 0.6 |
| ***10–20-year-old*** | 12 | 7 | 0.2 |
| ***20+ year-old*** | 15 | 13 | 0.5 |
| ***PfSBP1*** | **11** | **9** | **0.3** |
| ***5–10-year-old*** | 5 | 7 | 0.7 |
| ***10–20-year-old*** | 12 | 7 | 0.2 |
| ***20+ year-old*** | 15 | 10 | 0.1 |

***Table S3***. **Overall and age stratified observed seroprevalence by cohort**. Group differences were assessed using $\mathcal{X}^{2}$ tests using the *chisq.test* function in R and p-values are reported in the table.

- Lines 125, 305, and 377
  In many journals, citation of "in review" or "in preparation" publication is not allowed. Please double-check the regulation.

Citations have been updated to refer to the published version of the papers (lines 118, 346 and 419).

- Line 402-407
  The paragraph should be written in the discussion section, not in the method section.

Following the suggestion of Reviewer 1, the paragraph has been moved from the method to the discussion section (lines 291 – 296).

**Reviewer #2**

This paper extend sero-catalytic models to jointly model up to three antibody responses to P. falciparum, and validate reconstructions against long-term clinical incidence data from Dielmo. The central message, that multiplex (especially AMA1+GLURP) provides more accurate historical reconstruction than singleplex is well-motivated and likely to be of interest for malaria surveillance programs and the growing multiplex serology community. That said, several methodological and reporting issues need to be addressed to ensure the conclusions are fully supported and reproducible.

Thank you to the reviewer for their positive assessment of our work. We believe that all of the highlighted concerns are addressed below.

**Major comments:**

- the Authors validate models against clinical incidence time series and report that you digitized incidence data using a pixel measurer, then applied a "conversion coefficient" that minimizes MSE between model λ and observed incidence.

Concerns / requests: Please clarify whether the conversion coefficient is estimated separately for each model. If so, each model is effectively given an additional fitted degree of freedom, which complicates comparability and can partially “bake in” the validation signal.

The reviewer is correct that a conversion coefficient is estimated separately for each model. This has now been noted in the methods section (lines 480 – 483).

“For each model, a conversion coefficient was applied to relate the seroconversion rate over time to the number of clinical cases per person year. This was calculated by minimizing the mean standard error (MSE) between the data on clinical cases and the modelled seroconversion rate times the conversion coefficient.”

This does give a degree of flexibility to capture the average transmission over time, but does not provide the model with the flexibility to reconstruct how transmission changes over time.

1) the Authors can consider alternative validation summaries that do not require global MSE-minimizing scaling (e.g. correlation of temporal trends; matching a single anchor year; or using hold-out periods such as calibrating scale on early years and evaluating on later years, or vice versa).

Thank you for this suggestion. Although we did consider alternative summaries, we believe that the MSE approach provided an optimal way to validate against the data from all years.

Please provide the digitized incidence dataset and the digitization procedure (software, resolution, any smoothing), and quantify digitization uncertainty (e.g., repeated digitizations / sensitivity bounds). This is important because the digitized series is a key "ground truth" for ranking models.

We agree that the digitization method has not been developed enough in the manuscript and that the digitized incidence dataset should have been included. We added this paragraph to the methods section and shared the dataset (line 477).

“Data from Trape *et al* were digitized using the Pixel Ruler v3.1 app.”

And the following extended description is provided in the Supplementary Information (page 9).

“Data from Figure 3.C of <https://doi.org/10.1016/S1473-3099(14)70712-1> were digitized using the Pixel Ruler v3.1 app. For each of the 23 years, the number of pixels comprising the bar representing the number of fevers per person per year caused by *P. falciparum* was measured. The measurement resolution, determined by the pixel-to-data scaling of the y-axis, is 0.11 *P. falciparum* fevers per person per year per pixel. Digitization of a data point was repeated ten times and showed a variability of +/- 1 pixel or 0.11 *P. falciparum* fevers per person per year. The digitized dataset including pixel measurement can be found in the supplementary files.”

2) The Authors use a GMM to classify serostatus and choose a cutoff corresponding to 99% specificity because other cutoffs had variable sensitivity/specificity across antigens.

Requests:

- Provide a sensitivity analysis showing how main results (estimated change point drop the magnitude, and model ranking) change under the other candidate cutoffs (Youden; mean+2sd).

Following the suggestion of Reviewer 2 we added a sensitivity analysis to the supplementary files (pages 24 – 27):

“A sensitivity analysis was conducted to assess the impact of choosing different cutoffs. We fitted a multiplex serocatalytic model including *Pf*AMA1 and PfGlurpR2 to serological data from Dielmo dichotomized with cutoffs corresponding to the Youden Index, a specificity (Sp) of 99.9%, and the observed $\mu_{neg}+2{sd}_{neg}$. In the manuscript, the cutoff used corresponded to a Sp = 99%. We compared the posterior distributions of the seroconversion rate $\lambda$, the time at which a drop in transmission occurred $tc$, and its magnitude $\delta$ across the four models to the estimates presented in the manuscript (Figure S25). The estimated seroconversion rates range from 0.1 year^-1^ for the cutoff used in the manuscript to 0.2 year^-1^ for the observed $\mu_{neg}+2{sd}_{neg}$. All models estimated a drop in transmission in 2004 or 2005, 11 or 12 years prior to 2016. The estimated magnitude of this decline is higher for the model presented in the manuscript compared to models fitted to data dichotomized by all other cutoffs. All models were capable of recapturing the data they have been fitted on and matched with the validation data (Figures S26 to S28).”

**Figure S25: Sensitivity analysis of cutoff on estimated model parameters.** Posterior distributions of the seroconversion rate, the time of drop in transmission and its magnitude. Parameters were estimated by a serocatalytic model including *Pf*AMA1 and PfGlurpR2 fitted to the 2016 and 2018 cohorts in Dielmo. For each of the three models, data were binarized using one of the following cutoffs: a cutoff corresponding to the Youden Index, one corresponding to a specificity (Sp) of 99.9%, the observed $\mu_{neg}+2{sd}_{neg}$. The cutoff corresponding to a Sp = 99% is the one used in the main analysis presented in the manuscript.

**Figure S26: Model comparison to data and validation using a Youden Index to define the cutoff for seropositivity.** Panel A compares Dielmo’s observed and model-predicted age-stratified seroprevalence, for a multiplex serocatalytic model including both PfAMA1 and PfGlurpR2 and considering one drop in transmission. Data were dichotomized using a cutoff corresponding to the Youden Index. In Panel B the seroincidence estimated by the same model over 25 years prior to the 2016 cross section is compared with external validation data on the observed number of cases per person per year.

***Figure S27: Model comparison to data and validation using the*** $\mu_{neg}+2{sd}_{neg}$ ***to define the cutoff for seropositivity.*** *Panel A compares Dielmo’s observed and model-predicted age-stratified seroprevalence, for a multiplex serocatalytic model including both PfAMA1 and PfGlurpR2 and considering one drop in transmission. Data were dichotomized using* $\mu_{neg}+2{sd}_{neg}$ *as the cutoff. In Panel B the seroincidence estimated by the same model over 25 years prior to the 2016 cross section is compared with external validation data on the observed number of cases per person per year.*

***Figure S28: Model comparison to data and validation using a cutoff corresponding to a 99.9% specificity to define seropositivity.*** *Panel A compares Dielmo’s observed and model-predicted age-stratified seroprevalence, for a multiplex serocatalytic model including both PfAMA1 and PfGlurpR2 and considering one drop in transmission. Data were dichotomized using a cutoff corresponding to a specificity of 99.9%. In Panel B the seroincidence estimated by the same model over 25 years prior to the 2016 cross section is compared with external validation data on the observed number of cases per person per year.*

- The use of French adult controls is helpful, but please discuss potential cross-reactivity/background and age differences versus Senegalese cohorts, and how that might affect the negative component and thresholds.

Reviewer 2 is correct to highlight that differences in antibody responses between French adults and the Senegalese population may bias the estimation of the cutoff. We added a paragraph to address this limitation in the discussion (lines 264 – 268).

“Firstly, the GMM assumes that the French population is seronegative and that the Senegalese population is composed of both seronegative and seropositive individuals. As there have been reported differences in total IgG antibody levels between European and African populations^27^, this may introduce some bias to estimated cutoffs.”.

3) The Authors assume a common serological incidence λ across antibody responses and estimate antigen-specific probabilities of seroconversion given exposure (γ). This is a reasonable starting point, but please strengthen justification and limitations:

- Explain when the "shared λ + antigen-specific γ" structure is identifiable and biologically interpretable (e.g. λ as exposure FOI vs "sero-incidence" of acquiring any response).

Following the suggestion of Reviewer 2, we provided additional explanation regarding $\lambda$, $\gamma$ and the identifiability constraint caused by the structure of the model in the method section (lines 439 – 444).

“Seroconversion depends on a subject being exposed to a pathogen and this exposure leading to increased antibody response causing a change of serostatus. The incidence and the probability of seroconverting are combined into the seroconversion rate in univariate serocatalytic models. In multiplexed models, $\lambda$ is the incidence and the probability of seroconverting to antigen *A* after exposure is represented by $\gamma$_A_. Identifiability depends on the balance between the additional information provided by each antigen and their correlation.”

- Consider discussing whether differential boosting (re-exposure affecting antibody positivity) could violate a constant seroreversion interpretation, particularly as transmission declines.
- Please discuss that “effective seroreversion” can depend on boosting/reinfection (and therefore on transmission intensity), so the assumption that ρ is transferable across villages may not strictly hold.

We have grouped these two comments and added a paragraph to discuss this limitation (lines 271 – 276).

“Thirdly, serocatalytic models do not account for boosting of antibody responses in seropositive individuals. In higher transmission settings, individuals are expected to have higher antibody levels which have been demonstrated to be associated with lower seroconversion rates^28^, violating the assumption of constant seroreversion. Therefore, seroreversion rates may not be constant across differing transmission settings.”

4) A key limitation is that Dielmo surveys did not include children under 5, and you address convergence by using informative priors on ρ from Ndiop.

Requests:

- Provide sensitivity analyses showing how Dielmo inferences change under alternative priors for ρ (e.g., wider priors; priors centered on different Ndiop antigens; or leave-one-antigen-out priors).

In response to this suggestion from Reviewer 2 and a very similar suggestion from Reviewer 3, we added a sensitivity analysis to the supplementary files (pages 27 – 31):

“In the models presented in the manuscript, the prior on the seroreversion rate $\rho$ for a given antigen was defined as followes: $\rho_{antigen}\mathcal{\sim N(}\mu_{antigen}^{NDiop}, \sigma_{antigen}^{NDiop})$, with $\mu_{antigen}^{NDiop}$ and $\sigma_{antigen}^{NDiop}$ being the mean and standard deviation of the posterior distribution of the seroreversion rate estimated on data from Ndiop. A sensitivity analysis was conducted to assess the impact of different priors. We fitted three multiplexed serocatalytic models including *Pf*AMA1 and *Pf*GlurpR2 to Dielmo serological data. For the first one, wider priors were defined as $\rho_{antigen}\mathcal{\sim N(}\mu_{antigen}^{NDiop}, {3\sigma}_{antigen}^{NDiop})$, for the second one $\sigma_{PfGlurpR2}\mathcal{\sim N(}\mu_{PfGlurpR2}^{NDiop},\sigma_{PfGlurpR2}^{NDiop})$ and $\sigma_{PfAMA1} \sim exp(10)$ and for the last $\sigma_{PfAMA1}\mathcal{\sim N(}\mu_{PfAMA1}^{NDiop},\sigma_{PfAMA1}^{NDiop})$ and $\sigma_{PfGlurpR2} \sim exp(10)$.

We compared the posterior distributions of the seroconversion rate $\lambda$, the time at which a sharp drop in transmission occurred $tc$, and its magnitude $\delta$ with the estimations presented in the manuscript (Figure S29). Estimates from the four models were highly similar, with broadly overlapping posterior distributions. All models were capable of recapturing the data they have been fitted on and matched with the validation data (Figures S30 to S32).”

***Figure S29: Sensitivity analysis of choice of prior distributions.*** *Posterior distributions of the seroconversion rate, the time of a sharp drop in transmission and its magnitude. Parameters were estimated by a serocatalytic model including PfAMA1 and PfGlurpR2 fitted to the 2016 and 2018 cohorts in Dielmo. Priors differed for each model. For the first one, wider priors were defined as* $\rho_{antigen}\mathcal{\sim N(}\mu_{antigen}^{NDiop}, 3\sigma_{antigen}^{NDiop})$*, for the second one* $\sigma_{PfGlurpR2}\mathcal{\sim N(}\mu_{PfGlurpR2}^{NDiop},\sigma_{PfGlurpR2}^{NDiop})$ *and* $\sigma_{PfAMA1}$ *was uninformative (*$\sigma_{PfAMA1} \sim exp(10)$*) and for the last* $\sigma_{PfAMA1}\mathcal{\sim N(}\mu_{PfAMA1}^{NDiop},\sigma_{PfAMA1}^{NDiop})$ *and* $\sigma_{PfGlurpR2}$ *was uninformative (*$\sigma_{PfGlurpR2} \sim exp(10)$*). In the model presented in the manuscript priors on both antigens were defined as* $\rho_{antigen}\mathcal{\sim N(}\mu_{antigen}^{NDiop}, \sigma_{antigen}^{NDiop})$*.*

***Figure S30: Model comparison to data and validation.*** *Priors on the seroconversion rate were defined as* $\rho_{antigen}\mathcal{\sim N(}\mu_{antigen}^{NDiop}, 3\sigma_{antigen}^{NDiop})$*, with* $\mu_{antigen}^{NDiop}$ and $\sigma_{antigen}^{NDiop}$ being the mean and standard deviation of the posterior distribution of the seroreversion rate estimated on data from Ndiop. *Panel A compares Dielmo’s observed and model-predicted age-stratified seroprevalence, for a multiplex serocatalytic model including both PfAMA1 and PfGlurpR2 and considering one drop in transmission. In Panel B the seroincidence estimated by the same model over 25 years prior to the 2016 cross section is compared with external validation data on the observed number of cases per person per year.*

***Figure S31: Model comparison to data and validation.*** *Priors on the seroconversion rate were defined as* $\sigma_{PfGlurpR2}\mathcal{\sim N(}\mu_{PfGlurpR2}^{NDiop},\sigma_{PfGlurpR2}^{NDiop})$ and $\sigma_{PfAMA1} \sim\exp\left( 10 \right),$*with* $\mu_{PfGlurpR2}^{NDiop}$ and $\sigma_{PfGlurpR2}^{NDiop}$ being the mean and standard deviation of the posterior distribution of the seroreversion rate estimated on data from Ndiop. *Panel A compares Dielmo’s observed and model-predicted age-stratified seroprevalence, for a multiplex serocatalytic model including both PfAMA1 and PfGlurpR2 and considering one drop in transmission. In Panel B the seroincidence estimated by the same model over 25 years prior to the 2016 cross section is compared with external validation data on the observed number of cases per person per year.*

***Figure S32: Model comparison to data and validation.*** *Priors on the seroconversion rate were defined as* $\sigma_{PfGlurpR2}\mathcal{\sim N(}\mu_{PfAMA1}^{NDiop},\sigma_{PfAMA1}^{NDiop})$ and $\sigma_{PfGlurpR2} \sim\exp\left( 10 \right),$*with* $\mu_{PfAMA1}^{NDiop}$ and $\sigma_{PfAMA1}^{NDiop}$ being the mean and standard deviation of the posterior distribution of the seroreversion rate estimated on data from Ndiop. *Panel A compares Dielmo’s observed and model-predicted age-stratified seroprevalence, for a multiplex serocatalytic model including both PfAMA1 and PfGlurpR2 and considering one drop in transmission. In Panel B the seroincidence estimated by the same model over 25 years prior to the 2016 cross section is compared with external validation data on the observed number of cases per person per year.*

5) The Authors report that only ~60% of multi-antigen models converged.
   Requests:

- Provide sensitivity analyses showing how Dielmo inferences change under alternative priors for ρ

This sensitivity analysis is described in the response to point 4 above.

- Discuss whether selecting only converged models could bias conclusions about "two antigens vs three antigens" if three-antigen models fail preferentially for informative but correlated sets.

The reviewer is correct that the process of including only converged models may bias our interpretation of the results. This is included as a limitation in the discussion (lines 300 – 305).

“The model failed to converge for antigens that were of poor quality (e.g. PfMSP2-CH150) or for antigens that do not generate a long-lasting antibody response, resulting in a lack of age-dependent increase (e.g. PfSEA1, PfSBP1, PfHSP40). For 2 antigen models, 11/15 (73%) evaluated models converged, and for 3 antigen models 10/20 (50%) evaluated models converged. The lower proportion of models converging for larger number of antigens may be attributable to correlations between antibody responses to different antigens.”

6) The Authors state that 2016 and 2018 are modeled simultaneously with the same transmission pattern shifted by 2 years.

Please clarify:

- The exact likelihood contribution of each cross-section (are they treated as independent cross-sections with shared parameters except for calendar shift?).

In the methods section (lines 452 – 455) we expanded the paragraph describing how the two cohorts were implemented in the model and added the likelihood contribution of each cross-section as suggested by Reviewer 2.

“Apart from shared parameters, the likelihood of each cross-section is independent and modelled using multinomial distributions. Every sample contributes equally to the likelihood despite some participants being included in both cohorts.”

- Whether repeated individuals appear in both surveys (you mention substantial overlap), and if so, whether ignoring within-person correlation could affect uncertainty and model fit.

We added a paragraph to the discussion to highlight limitations caused by ignoring repeated measurements on a subject (lines 277 – 280).

“Although many residents of Dielmo were included in both the 2016 and 2018 cross-sections, repeat samples from the same individuals were treated independently in the likelihood. Development of a modelling framework accounting for longitudinal sampling may lead to improved estimates of seroconversion and seroreversion rates.”

**Minor comments**

- Provide a brief intuitive explanation of what γ represents for non-modelers (probability of seroconversion given exposure), and how it differs from antigen-specific λ.

In response to this minor comment, and one of this reviewer’s major comment #3, we have provided a more intuitive explanation of $\gamma$ in the methods section (lines 439 – 444).

“Seroconversion depends on a subject being exposed to a pathogen and this exposure leading to increased antibody response causing a change of serostatus. The incidence and the probability of seroconverting are combined into the seroconversion rate in univariate serocatalytic models. In multiplexed models, $\lambda$ is the incidence and the probability of seroconverting to antigen A after exposure is represented by $\gamma_{A}$. Identifiability depends on the balance between the additional information provided by each antigen and their correlation.”

- Consider adding a schematic figure (main text, not only supplement) that explains the multiplex compartments and how correlation is captured.

We left the schematic figure in the supplementary file, but we linked it to the results to better explain how the model captures correlation (page 10).

“Most adults older than 20 years old are seropositive to both PfAMA1 and PfGlurpR2. After being exposed to *P. falciparum* at rate $\lambda$, 481/560 individuals are seropositive to at least one antigen and most of them (354/481) seroconverted to both PfAMA1 and PfGlurpR2, with 62/481 participants seropositive to PfAMA1 only and 65/481 seropositive to PfGlurpR2 only. The probabilities to seroconvert to a given antigen after exposure are given by the parameters $\gamma_{antigen}$ and the probability to seroconvert to a given serostatus is given by the combination of $\lambda$ and $\gamma_{antigens}$. For example, the probability to seroconvert to PfAMA1 only is the probability of being exposed $\lambda$ multiplied by the probability that exposure caused seroconversion to PfAMA1, $\gamma_{PfAMA1}$, but not to PfGlurpR2, $(1- \gamma_{PfGlurpR2})$.”

|  | PfAMA1 | |
| --- | --- | --- |
| PfGlurpR2 | $P_{0}=79$ | $P_{PfAMA1}=62$ |
|  | $P_{PfGlurpR2}=65$ | $P_{PfAMA1, PfGlurpR2}=354$ |

**Table S4: Contingency table of the different observed serostatus for the combination of PfAMA1 and PfGlurpR2 and for adults of more than 20 years**. The distribution is significantly different (Pearson’s $\chi^{2}$-test, p-value <2.2e-16) of a distribution reflecting independence of the two antibody responses.

**Typos**
- "serocatalytic" vs "sero-catalytic" should be standardized;
- replace "sensibility" by "sensitivity" in Fig 2 description.

We corrected typos is the manuscript and figure description.

**Reviewer #3**

This is a clear, well-written, and methodologically strong paper that makes a meaningful contribution to sero-epidemiological modeling by extending serocatalytic approaches to multiplex data. The empirical application is compelling, and the manuscript is generally well structured and easy to follow. My comments focus on clarifying some key assumptions, potentially strengthening aspects of the validation, and improving precision in interpretation and wording.

Thank you to this reviewer for their positive assessment of our work.

**Major comments**

- External validation framework

The validation strategy relies on transforming the model-derived serological incidence using a fitted conversion coefficient to match observed case data. This is practical but also reduces the independence of the validation exercise and may inflate apparent model performance. Clarify this limitation more explicitly and discuss how conclusions might change under a fully independent validation framework.

The reviewer is correct that the use of a fitted conversion coefficient to match observed data reduces the independence of the validation exercise – a similar point was made by reviewer #2. This framework could be made more robust by evaluating the model on multiple settings where both serological data and historical clinical incidence data are available. This has been noted in the discussion (lines 288 – 290).

“The framework would benefit from additional validation of the association between seroconversion rate and historical clinical incidence in different epidemiological settings.”

- Use of priors and parameter identifiability

The model addresses identifiability issues (particularly between λ and ρ) by incorporating priors derived from Ndiop into the Dielmo analysis. This is reasonable but introduces an implicit assumption of transferability between settings that is not formally modeled. I suggest to expand the discussion of how sensitive the results may be to these priors and the implications of borrowing information across sites.

This is an excellent point, which we address in several ways. Firstly, there are indeed issues of model identifiability which is discussed as a limitation (lines 300 – 305). Secondly, we do make the assumption of transferability of the seroconversion rate ρ between settings (discussed in lines 285 – 390). Finally, we have also included additional sensitivity analyses investigating the effects of alternative prior distributions (see response to Reviewer #2’s point 4 above).

- Model convergence and selection bias

A substantial proportion of multi-antigen models did not converge, and inference is restricted to those that did. This raises the possibility that conclusions are influenced by the subset of antigen combinations with favorable statistical properties. I suggest to discuss whether convergence itself may be informative and whether excluded models differ systematically.

The reviewer is correct that the process of including only converged models may bias our interpretation of the results. This is included as a limitation in the discussion (lines 300 – 305).

“The model failed to converge for antigens that were of poor quality (e.g. PfMSP2-CH150) or for antigens that do not generate a long-lasting antibody response, resulting in a lack of age-dependent increase (e.g. PfSEA1, PfSBP1, PfHSP40). For 2 antigen models, 11/15 (73%) evaluated models converged, and for 3 antigen models 10/20 (50%) evaluated models converged. The lower proportion of models converging for larger number of antigens may be attributable to correlations between antibody responses to different antigens.”

- Generalisability of the framework

In the discussion the authors suggest that the framework could be applied broadly to other pathogens: "...however this methodology could also be used on a wide variety of pathogens (arboviruses but also respiratory viruses, enteroviruses...) and, like serocatalytic models they can be applied to population in a broad variety of transmission settings."

This is a reasonable expectation and I generally agree, particularly given the demonstrated benefits of combining multiple antibody responses and the ability of the framework to identify diminishing returns from additional markers. However, the results also indicate that performance depends on the informativeness and discriminative capacity of individual antigens, as well as model convergence. It would be helpful to more clearly articulate these conditions under which multiplex approaches are likely to yield gains. Perhaps another sentence or two of caution and specificity are warranted. Under which conditions might this work well with other pathogens or other transmission settings, etc.?

As suggested by Reviewer 3, we emphasized this framework’s requirements (lines 311 – 316):

“In this study, new models for serological surveillance have been applied to inform malaria transmission in an endemic region in Senegal. Required conditions to implement these models are that each individual antigen has the capacity to discriminate exposed from nonexposed individuals, and that different antigens provide independent information. This methodology could also be used on a wide variety of pathogens (arboviruses, respiratory viruses, enteric viruses) and, like serocatalytic models they can be applied to populations in a broad variety of transmission settings.”

**Minor comments**

- Dichotomization of antibody responses

Converting continuous antibody measurements into binary serostatus may discard useful information. This is of course common practice. A brief discussion of the trade-offs (or potential extensions using continuous measures) would strengthen the manuscript.

As suggested by Reviewer 3 we added a paragraph on the loss of information caused by data reduction (lines 268 – 271):

“Secondly, dichotomization of continuous antibody titres to determine serostatus involves loss of information. Alternative models have been developed to avoid this assumption allowing serocatalytic models to be fitted directly to continuous antibody titres^28^. Further work could extend such methods to multiplexed serocatalytic models.”

- Age structure limitations
  The absence of children under five in Dielmo is an important limitation for serocatalytic inference, as younger ages are particularly informative for recent transmission. This could be emphasized more clearly.

As suggested by Reviewer 3 we emphasized more clearly the limitation caused by the absence of young children (lines 283 – 290):

“There were limitations to the data used in this study, most notably the absence of samples from children under 5 years from Dielmo. Serological data from young participants are particularly informative in serocatalytic models. The absence of data from these samples prevents reconstruction of changes in malaria transmission in the past five years. This was mitigated by informing priors with the posterior distribution from singleplex models fitted to data from Ndiop which contained samples from people of all ages. The framework would benefit from additional validation of the association between seroconversion rate and historical clinical incidence in different epidemiological settings.”

- Terminology and wording
  Use “data are” rather than “data is” (throughout).
  “Cross sectional” might better be “cross-sectional.”
  “Sensibility” (e.g., Fig. 2 caption) should be “sensitivity.”

We corrected this terminology and wording in the manuscript.

**Editors’ comments**

1) Please provide an Author Summary. This should appear in your manuscript between the Abstract (if applicable) and the Introduction, and should be 150-200 words long. The aim should be to make your findings accessible to a wide audience that includes both scientists and non-scientists. Sample summaries can be found on our website under Submission Guidelines:
   [https://journals.plos.org/ploscompbiol/s/submission-guidelines#loc-parts-of-a-submission](https://urldefense.com/v3/__https:/journals.plos.org/ploscompbiol/s/submission-guidelines*loc-parts-of-a-submission__;Iw!!JFdNOqOXpB6UZW0!pFyaX_T5yNlFs2w5-G1rDeWk0fh9_C9L5nA8_t80y79Vzo8mpi_YOnxLIx8w4k48T5ABfW2EnJh5BQYIKrqy8331$)

As requested by the editors, we added an Author Summary (lines 56 – 64).

2) We have noticed that you have uploaded Supporting Information files, but you have not included a complete list of legends. Please add a full list of legends for your Supporting Information files after the references list.

The complete list of legends for all figures, tables and equations has been added to the main manuscript after the reference list (lines 664 – 867).

3) Please provide a detailed Financial Disclosure statement. This is published with the article. It must therefore be completed in full sentences and contain the exact wording you wish to be published.
   a. Please clarify all sources of financial support for your study. List the grants, grant numbers, and organizations that funded your study, including funding received from your institution. Please note that suppliers of material support, including research materials, should be recognized in the Acknowledgements section rather than in the Financial Disclosure
   b. State the initials, alongside each funding source, of each author to receive each grant. For example: "This work was supported by the National Institutes of Health (####### to AM; ###### to CJ) and the National Science Foundation (###### to AM)."
   c. State what role the funders took in the study. If the funders had no role in your study, please state: "The funders had no role in study design, data collection and analysis, decision to publish, or preparation of the manuscript."
   d. If any authors received a salary from any of your funders, please state which authors and which funders..
      If you did not receive any funding for this study, please simply state: u201cThe authors received no specific funding for this work.u201d

We added and updated the financial disclosure section of the submission system to a full sentences description of all financial aid received for this project (lines 489 – 494).

“This work was supported by the European Research Council (MultiSeroSurv, 852373 to MW), and the French government’s “Integrative Biology of Emerging Infectious Diseases” (Investissement d’Avenir grant ANR-10-LABX-62-IBEID to MW) and INCEPTION programs (Investissement d’Avenir grant ANR-16-CONV-0005 to MW). The funders had no role in study design, data collection and analysis, decision to publish, or preparation of the manuscript.”

4) Your current Financial Disclosure states, "The author(s) received no specific funding for this work.".
   However, your funding information on the submission form indicates receiving funds. 
   Please indicate by return email the full and correct funding information for your study and confirm the order in which funding contributions should appear. Please be sure to indicate whether the funders played any role in the study design, data collection and analysis, decision to publish, or preparation of the manuscript.

This box has been ticked in error. We have updated the Financial Disclosure to be consistent with the description above.
