# Supplementary material for: Reconstruction of historical malaria transmission in Senegal using multiplex serocatalytic models
Source: PLoS Comput Biol. 2026 Jul 27;22(7):e1013630. doi: 10.1371/journal.pcbi.1013630 (PMC13423172; doi:10.1371/journal.pcbi.1013630)
Supplement: Attachment — Submitted filename: Response_to_Reviewers_auresp_2.docx [file pcbi.1013630.s006.docx]

**Infectious Disease Epidemiology
& Analytics G5 Unit**

Institut Pasteur

25-28 Rue du Dr Roux

Paris 75015

France

**Dr Michael White**

michael.white@pasteur.fr

24^th^ June 2026

Dear Editor,

We would like to resubmit a revised version of our manuscript, “Reconstruction of historical malaria transmission in Senegal using multiplex serocatalytic models”, for consideration for publication in *PLOS Computational Biology*.

We would like to thank again the reviewers for their attention to this manuscript. The corrections suggested by reviewer #2 have been accounted for.

We look forward to your response.

Yours sincerely,

Dr Michael White, on behalf of the authors

**Reviewer #1**

I don't have any other suggestions from my end to improve the manuscript.

Thank you again to this reviewer for the positive assessment of our work.

**Reviewer #2**

I thank the authors for their careful and comprehensive revision of the manuscript. I appreciate the detailed point-by-point responses and the additional analyses that were added, particularly the sensitivity analyses for seropositivity cutoffs and priors on the seroreversion rate, the expanded description of the digitized incidence data, and the clearer discussion of model identifiability, convergence, seasonality, repeated samples, and generalisability.

The revised manuscript is substantially improved, and I am satisfied that my major concerns have been adequately addressed. I therefore support acceptance of the manuscript. I have only a few minor comments that the authors may wish to address during final revision or copy-editing:

Thank you to this reviewer for their thorough assessment of our work.

- Please standardize terminology throughout the manuscript and supplementary material. In particular, I noticed remaining variation between “serocatalytic” and “sero-catalytic”, “seropositive/seronegative” and “sero-positive/sero-negative”, and “GlurpR2”, “GLURP”, “PfGLURP.R2”, and “PfGlurp.R2”. A single nomenclature should be used consistently across the title, abstract, results, figures, legends, and supplementary information.

We standardised terminology throughout the manuscript.

- “PfGlurpR2” was selected and corrected in the manuscript L50 and 53
- “Seroconversion” was selected and corrected in axis title of Figure 3 and Figure S24, Figure S26-28 and Figure S30-32
- “serocatalytic” was selected and corrected in the cover letter but no occurrence of sero-catalytic was found in the manuscript, supplementary, or figures
- “seropositive/ seronegative” were selected but no occurrence of sero-positive/ sero-negative was found in the manuscript, supplementary, or figures
- Some typographical issues appear to remain in the manuscript or supplementary legends. Examples include “determinantion” in the Figure 2 legend, “Kruskall Wallis” instead of “Kruskal-Wallis”, and remaining occurrences of “sensibility” in some supplementary figure legends, where “sensitivity” is intended. A careful final copy-edit is recommended.

Typographical issues were corrected L179, L475 and pages 4-8 and 24-28 in the supplementary information.

Overall, the revised manuscript makes a valuable methodological contribution to multiplex serological surveillance and provides a useful application to malaria transmission reconstruction in Senegal. The additional analyses and expanded limitations have strengthened the manuscript considerably.

**Reviewer #3**

The authors have done a great job of addressing all of my critiques and comments. This is a nice paper.

Thank you again to this reviewer for their positive assessment of our work.
