# Supplementary material for: Reconstruction of historical malaria transmission in Senegal using multiplex serocatalytic models
Source: PLoS Comput Biol. 2026 Jul 27;22(7):e1013630. doi: 10.1371/journal.pcbi.1013630 (PMC13423172; doi:10.1371/journal.pcbi.1013630)
Supplement: Peer review history

# Peer review: Decision Letter 0

**DOI:** 10.1371/journal.pcbi.1013630.r001

**Authors:** Necibe Tuncer, Benjamin Althouse

29 Mar 2026

PCOMPBIOL-D-25-02114

Reconstruction of historical malaria transmission in Senegal using multiplex sero-catalytic models.

PLOS Computational Biology

Dear Dr. BAUDEMONT,

Thank you for submitting your manuscript to PLOS Computational Biology. After careful consideration, we feel that it has merit but does not fully meet PLOS Computational Biology's publication criteria as it currently stands. Therefore, we invite you to submit a revised version of the manuscript that addresses the points raised during the review process.

Please submit your revised manuscript by May 29 2026 11:59PM. If you will need more time than this to complete your revisions, please reply to this message or contact the journal office at ploscompbiol@plos.org. When you're ready to submit your revision, log on to https://www.editorialmanager.com/pcompbiol/ and select the 'Submissions Needing Revision' folder to locate your manuscript file.

Please include the following items when submitting your revised manuscript:

* A letter that responds to each point raised by the editor and reviewer(s). You should upload this letter as a separate file labeled 'Response to Reviewers'. This file does not need to include responses to formatting updates and technical items listed in the 'Journal Requirements' section below.

* A marked-up copy of your manuscript that highlights changes made to the original version. You should upload this as a separate file labeled 'Revised Manuscript with Track Changes'.

* An unmarked version of your revised paper without tracked changes. You should upload this as a separate file labeled 'Manuscript'.

If you would like to make changes to your financial disclosure, competing interests statement, or data availability statement, please make these updates within the submission form at the time of resubmission. Guidelines for resubmitting your figure files are available below the reviewer comments at the end of this letter

We look forward to receiving your revised manuscript.

Kind regards,

Necibe Tuncer, Ph.D.

Academic Editor

PLOS Computational Biology

Benjamin Althouse

Section Editor

PLOS Computational Biology

**Additional Editor Comments:**

Dear authors, please address the reviewers’ comments and submit a revised manuscript for further consideration for publication.

**Journal Requirements:**

If the reviewer comments include a recommendation to cite specific previously published works, please review and evaluate these publications to determine whether they are relevant and should be cited. There is no requirement to cite these works unless the editor has indicated otherwise.

1) Please provide an Author Summary. This should appear in your manuscript between the Abstract (if applicable) and the Introduction, and should be 150-200 words long. The aim should be to make your findings accessible to a wide audience that includes both scientists and non-scientists. Sample summaries can be found on our website under Submission Guidelines:

https://journals.plos.org/ploscompbiol/s/submission-guidelines#loc-parts-of-a-submission

2) We have noticed that you have uploaded Supporting Information files, but you have not included a complete list of legends. Please add a full list of legends for your Supporting Information files after the references list.

3) Please provide a detailed Financial Disclosure statement. This is published with the article. It must therefore be completed in full sentences and contain the exact wording you wish to be published.

1) Please clarify all sources of financial support for your study. List the grants, grant numbers, and organizations that funded your study, including funding received from your institution. Please note that suppliers of material support, including research materials, should be recognized in the Acknowledgements section rather than in the Financial Disclosure

2) State the initials, alongside each funding source, of each author to receive each grant. For example: "This work was supported by the National Institutes of Health (####### to AM; ###### to CJ) and the National Science Foundation (###### to AM)."

3) State what role the funders took in the study. If the funders had no role in your study, please state: "The funders had no role in study design, data collection and analysis, decision to publish, or preparation of the manuscript."

4) If any authors received a salary from any of your funders, please state which authors and which funders..

If you did not receive any funding for this study, please simply state: u201cThe authors received no specific funding for this work.u201d

4) Your current Financial Disclosure states, "The author(s) received no specific funding for this work.".

However, your funding information on the submission form indicates receiving funds.

Please indicate by return email the full and correct funding information for your study and confirm the order in which funding contributions should appear. Please be sure to indicate whether the funders played any role in the study design, data collection and analysis, decision to publish, or preparation of the manuscript.

**Reviewers' comments:**

Reviewer's Responses to Questions

**Comments to the Authors:**

**Please note here if the review is uploaded as an attachment.**

Reviewer #1: Baudemont et al. developed a serocatalytic model that allows historical malaria endemicity to be estimated from antibody titers measured in a cross-sectional survey. It is an interesting attempt, and the model generation process is reasonable. However, it is questionable for the utility of this model in future studies, and two of the main assumptions used in this study may or may not be appropriate. It would be ideal if the authors could conduct additional analysis to answer the questions, but if not, the points should be discussed as additional limitations of the study.

Major comments

1) One change (reduction) in transmission

While the authors had the clinical record in the study sites for a long time, the application of the model is when we don't have such historical data (if we have the data, there is no need to speculate prior endemicity from serological data). A study site could experience multiple reductions, increases (such as seen during the COVID-19 pandemic era), and a combination of both. Therefore, the utility of the model, which assumes there was one sharp drop in transmission in the past, is questionable. How applicable is the model described in this paper when there is no historical data?

2) Seasonality

While malaria transmission in Dielmo is perennial, Ndiop is not. Therefore, the sample collection time (e.g., middle of the dry season, end of the transmission season) should be specified. Although it depends on the endemicity, in general, antibody titers are higher at the peak of malaria season compared to those in the middle of the dry season. What is the rationale for ignoring the seasonal effect?

3) Constant seroconversion rate (ramda)

Based on Figure 3a (and also seen in many sero-epidemiology studies), the sero-prevalence generally increases rapidly <~20-30 years old, but it reaches a plateau after ~30 years old. What is the rationale for the constant seroconversion rate?

Minor comments

1) Line 112: up to 500 antibody responses

Dr. Tsuboi and his group have published several papers where they used >1800 antigens (e.g., PMID28378857).

2) parasite strain, and amino acid positions for the recombinant proteins

Except for PfMSP2-Dd2 and PfMSP2-CH150, the strain of parasite (3D7?) that was selected was not specified. In addition, readers cannot tell whether full-length or truncated (e.g., amino acid XX to XX) proteins were made. That information should be written to replicate the study.

3) Line 202: "two long-lived blood stage antigens".

What does it mean by "two long-lived blood stage antigens"? Since the study was conducted in highly endemic areas, we cannot tell whether a single infection induced long-lived antibody responses, continuous (asymptomatic) infections maintained high titers, or a mixture of both. In addition, based on Figure 1, the positive rate of PfMSP1 was also high as PfAMA1 or PfGlurpR2.

4) Figure 1 legend

Since figures and tables should stand by themselves, please explain the abbreviations of antigen names in the legend for Figure 1.

5) Is there any difference in sero-positivity depending on the year or location of sample collection?

While it is nice to present the individual data in Figure 1, there are too many points to judge whether there is any difference between 2016 and 2018, and/or between Ndiop and Dielmo. Please add the information in the main text, and if there a significant difference (s), please discuss whether the difference affects any model building or interpretation.

6) Lines 125, 305, and 377

In many journals, citation of "in review" or "in preparation" publication is not allowed. Please double-check the regulation.

7) Line 402-407

The paragraph should be written in the discussion section, not in the method section.

Reviewer #2: This paper extend sero-catalytic models to jointly model up to three antibody responses to P. falciparum, and validate reconstructions against long-term clinical incidence data from Dielmo. The central message, that multiplex (especially AMA1+GLURP) provides more accurate historical reconstruction than singleplex is well-motivated and likely to be of interest for malaria surveillance programs and the growing multiplex serology community.

That said, several methodological and reporting issues need to be addressed to ensure the conclusions are fully supported and reproducible.

Major comments:

the Authors validate models against clinical incidence time series and report that you digitized incidence data using a pixel measurer, then applied a "conversion coefficient" that minimizes MSE between model λ and observed incidence.

Concerns / requests:

Please clarify whether the conversion coefficient is estimated separately for each model. If so, each model is effectively given an additional fitted degree of freedom, which complicates comparability and can partially “bake in” the validation signal.

1. the Authors can consider alternative validation summaries that do not require global MSE-minimizing scaling (e.g. correlation of temporal trends; matching a single anchor year; or using hold-out periods such as calibrating scale on early years and evaluating on later years, or vice versa).

Please provide the digitized incidence dataset and the digitization procedure (software, resolution, any smoothing), and quantify digitization uncertainty (e.g., repeated digitizations / sensitivity bounds). This is important because the digitized series is a key "ground truth" for ranking models.

2. The Authors use a GMM to classify serostatus and choose a cutoff corresponding to 99% specificity because other cutoffs had variable sensitivity/specificity across antigens.

Requests:

- Provide a sensitivity analysis showing how main results (estimated change point drop the magnitude, and model ranking) change under the other candidate cutoffs (Youden; mean+2sd).

- The use of French adult controls is helpful, but please discuss potential cross-reactivity/background and age differences versus Senegalese cohorts, and how that might affect the negative component and thresholds.

3. The Authors assume a common serological incidence λ across antibody responses and estimate antigen-specific probabilities of seroconversion given exposure (γ).

This is a reasonable starting point, but please strengthen justification and limitations:

- Explain when the "shared λ + antigen-specific γ" structure is identifiable and biologically interpretable (e.g. λ as exposure FOI vs "sero-incidence" of acquiring any response).

- Consider discussing whether differential boosting (re-exposure affecting antibody positivity) could violate a constant seroreversion interpretation, particularly as transmission declines.

4. A key limitation is that Dielmo surveys did not include children under 5, and you address convergence by using informative priors on ρ from Ndiop.

Requests:

- Provide sensitivity analyses showing how Dielmo inferences change under alternative priors for ρ (e.g., wider priors; priors centered on different Ndiop antigens; or leave-one-antigen-out priors).

- Please discuss that “effective seroreversion” can depend on boosting/reinfection (and therefore on transmission intensity), so the assumption that ρ is transferable across villages may not strictly hold.

5. The Authors report that only ~60% of multi-antigen models converged.

Requests:

-Provide sensitivity analyses showing how Dielmo inferences change under alternative priors for ρ

- Discuss whether selecting only converged models could bias conclusions about "two antigens vs three antigens" if three-antigen models fail preferentially for informative but correlated sets.

6. The Authors state that 2016 and 2018 are modeled simultaneously with the same transmission pattern shifted by 2 years.

Please clarify:

- The exact likelihood contribution of each cross-section (are they treated as independent cross-sections with shared parameters except for calendar shift?).

- Whether repeated individuals appear in both surveys (you mention substantial overlap), and if so, whether ignoring within-person correlation could affect uncertainty and model fit.

Minor comments

- Provide a brief intuitive explanation of what γ represents for non-modelers (probability of seroconversion given exposure), and how it differs from antigen-specific λ.

- Consider adding a schematic figure (main text, not only supplement) that explains the multiplex compartments and how correlation is captured.

Typos:

- "serocatalytic" vs "sero-catalytic" should be standardized;

- replace "sensibility" by "sensitivity" in Fig 2 description.

Reviewer #3: Overall assessment

This is a clear, well-written, and methodologically strong paper that makes a meaningful contribution to sero-epidemiological modeling by extending serocatalytic approaches to multiplex data. The empirical application is compelling, and the manuscript is generally well structured and easy to follow. My comments focus on clarifying some key assumptions, potentially strengthening aspects of the validation, and improving precision in interpretation and wording.

Major comments

External validation framework

The validation strategy relies on transforming the model-derived serological incidence using a fitted conversion coefficient to match observed case data. This is practical but also reduces the independence of the validation exercise and may inflate apparent model performance.

Clarify this limitation more explicitly and discuss how conclusions might change under a fully independent validation framework.

Use of priors and parameter identifiability

The model addresses identifiability issues (particularly between λ and ρ) by incorporating priors derived from Ndiop into the Dielmo analysis. This is reasonable but introduces an implicit assumption of transferability between settings that is not formally modeled.

I suggest to expand the discussion of how sensitive the results may be to these priors and the implications of borrowing information across sites.

Model convergence and selection bias

A substantial proportion of multi-antigen models did not converge, and inference is restricted to those that did. This raises the possibility that conclusions are influenced by the subset of antigen combinations with favorable statistical properties.

I suggest to discuss whether convergence itself may be informative and whether excluded models differ systematically.

Generalisability of the framework

In the discussion the authors suggest that the framework could be applied broadly to other pathogens: "...however this methodology could also be used on a wide variety of pathogens (arboviruses but also respiratory viruses, enteroviruses...) and, like serocatalytic models they can be applied to population in a broad variety of transmission settings."

This is a reasonable expectation and I generally agree, particularly given the demonstrated benefits of combining multiple antibody responses and the ability of the framework to identify diminishing returns from additional markers. However, the results also indicate that performance depends on the informativeness and discriminative capacity of individual antigens, as well as model convergence. It would be helpful to more clearly articulate these conditions under which multiplex approaches are likely to yield gains. Perhaps another sentence or two of caution and specificity are warranted. Under which conditions might this work well with other pathogens or other transmission settings, etc.?

Minor comments

Dichotomization of antibody responses

Converting continuous antibody measurements into binary serostatus may discard useful information. This is of course common practice. A brief discussion of the trade-offs (or potential extensions using continuous measures) would strengthen the manuscript.

Age structure limitations

The absence of children under five in Dielmo is an important limitation for serocatalytic inference, as younger ages are particularly informative for recent transmission. This could be emphasized more clearly.

Terminology and wording

Use “data are” rather than “data is” (throughout).

“Cross sectional” might better be “cross-sectional.”

“Sensibility” (e.g., Fig. 2 caption) should be “sensitivity.”

**********

**Have the authors made all data and (if applicable) computational code underlying the findings in their manuscript fully available?**

The PLOS Data policy requires authors to make all data and code underlying the findings described in their manuscript fully available without restriction, with rare exception (please refer to the Data Availability Statement in the manuscript PDF file). The data and code should be provided as part of the manuscript or its supporting information, or deposited to a public repository. For example, in addition to summary statistics, the data points behind means, medians and variance measures should be available. If there are restrictions on publicly sharing data or code —e.g. participant privacy or use of data from a third party—those must be specified.

Reviewer #1: Yes

Reviewer #2: Yes

Reviewer #3: Yes

**********

PLOS authors have the option to publish the peer review history of their article (what does this mean?). If published, this will include your full peer review and any attached files.

If you choose “no”, your identity will remain anonymous but your review may still be made public.

**Do you want your identity to be public for this peer review?**  For information about this choice, including consent withdrawal, please see our Privacy Policy.

Reviewer #1: No

Reviewer #2: No

Reviewer #3: **Yes:** DM Parker

**Figure resubmission:**

While revising your submission, we strongly recommend that you use PLOS’s NAAS tool (https://ngplosjournals.pagemajik.ai/artanalysis) to test your figure files. NAAS can convert your figure files to the TIFF file type and meet basic requirements (such as print size, resolution), or provide you with a report on issues that do not meet our requirements and that NAAS cannot fix.

After uploading your figures to PLOS’s NAAS tool - https://ngplosjournals.pagemajik.ai/artanalysis, NAAS will process the files provided and display the results in the "Uploaded Files" section of the page as the processing is complete. If the uploaded figures meet our requirements (or NAAS is able to fix the files to meet our requirements), the figure will be marked as "fixed" above. If NAAS is unable to fix the files, a red "failed" label will appear above. When NAAS has confirmed that the figure files meet our requirements, please download the file via the download option, and include these NAAS processed figure files when submitting your revised manuscript.

**Reproducibility:**

To enhance the reproducibility of your results, we recommend that authors of applicable studies deposit laboratory protocols in protocols.io, where a protocol can be assigned its own identifier (DOI) such that it can be cited independently in the future. Additionally, PLOS ONE offers an option to publish peer-reviewed clinical study protocols. Read more information on sharing protocols at https://plos.org/protocols?utm_medium=editorial-email&utm_source=authorletters&utm_campaign=protocols

# Author comment: Author response to Decision Letter 1

**DOI:** 10.1371/journal.pcbi.1013630.r002

13 May 2026

Attachment Submitted filename: Response to Reviewers.docx

# Peer review: Decision Letter 1

**DOI:** 10.1371/journal.pcbi.1013630.r003

**Authors:** Necibe Tuncer, Benjamin Althouse

24 Jun 2026

PCOMPBIOL-D-25-02114R1

Reconstruction of historical malaria transmission in Senegal using multiplex serocatalytic models.

PLOS Computational Biology

Dear Dr. BAUDEMONT,

Thank you for submitting your manuscript to PLOS Computational Biology. After careful consideration, we feel that it has merit but does not fully meet PLOS Computational Biology's publication criteria as it currently stands. Therefore, we invite you to submit a revised version of the manuscript that addresses the points raised during the review process.

Please submit your revised manuscript by Aug 24 2026 11:59PM. If you will need more time than this to complete your revisions, please reply to this message or contact the journal office at ploscompbiol@plos.org. When you're ready to submit your revision, log on to https://www.editorialmanager.com/pcompbiol/ and select the 'Submissions Needing Revision' folder to locate your manuscript file.

Please include the following items when submitting your revised manuscript:

* A letter that responds to each point raised by the editor and reviewer(s). You should upload this letter as a separate file labeled 'Response to Reviewers'. This file does not need to include responses to formatting updates and technical items listed in the 'Journal Requirements' section below.

* A marked-up copy of your manuscript that highlights changes made to the original version. You should upload this as a separate file labeled 'Revised Manuscript with Track Changes'.

* An unmarked version of your revised paper without tracked changes. You should upload this as a separate file labeled 'Manuscript'.

If you would like to make changes to your financial disclosure, competing interests statement, or data availability statement, please make these updates within the submission form at the time of resubmission. Guidelines for resubmitting your figure files are available below the reviewer comments at the end of this letter.

As the corresponding author, your ORCID iD is verified in the submission system and will appear in the published article. PLOS supports the use of ORCID, and we encourage all coauthors to register for an ORCID iD and use it as well. Please encourage your coauthors to verify their ORCID iD within the submission system before final acceptance, as unverified ORCID iDs will not appear in the published article. Only the individual author can complete the verification step; PLOS staff cannot verify ORCID iDs on behalf of authors.

We look forward to receiving your revised manuscript.

Kind regards,

Necibe Tuncer, Ph.D.

Academic Editor

PLOS Computational Biology

Benjamin Althouse

Section Editor

PLOS Computational Biology

**Additional Editor Comments (if provided):**

The reviewers are satisfied with the revisions and responses provided. Before the manuscript can be accepted for publication, please address the final minor revision requested by Reviewer 2 and submit a revised version of the manuscript.

**Reviewers' comments:**

Reviewer's Responses to Questions

**Comments to the Authors:**

**Please note here if the review is uploaded as an attachment.**

Reviewer #1: I don't have any other suggestions from my end to improve the manuscript.

Reviewer #2: I thank the authors for their careful and comprehensive revision of the manuscript. I appreciate the detailed point-by-point responses and the additional analyses that were added, particularly the sensitivity analyses for seropositivity cutoffs and priors on the seroreversion rate, the expanded description of the digitized incidence data, and the clearer discussion of model identifiability, convergence, seasonality, repeated samples, and generalisability.

The revised manuscript is substantially improved, and I am satisfied that my major concerns have been adequately addressed. I therefore support acceptance of the manuscript. I have only a few minor comments that the authors may wish to address during final revision or copy-editing:

** Please standardize terminology throughout the manuscript and supplementary material. In particular, I noticed remaining variation between “serocatalytic” and “sero-catalytic”, “seropositive/seronegative” and “sero-positive/sero-negative”, and “GlurpR2”, “GLURP”, “PfGLURP.R2”, and “PfGlurp.R2”. A single nomenclature should be used consistently across the title, abstract, results, figures, legends, and supplementary information.

** Some typographical issues appear to remain in the manuscript or supplementary legends. Examples include “determinantion” in the Figure 2 legend, “Kruskall Wallis” instead of “Kruskal-Wallis”, and remaining occurrences of “sensibility” in some supplementary figure legends, where “sensitivity” is intended. A careful final copy-edit is recommended.

Overall, the revised manuscript makes a valuable methodological contribution to multiplex serological surveillance and provides a useful application to malaria transmission reconstruction in Senegal. The additional analyses and expanded limitations have strengthened the manuscript considerably.

Reviewer #3: The authors have done a great job of addressing all of my critiques and comments. This is a nice paper.

**********

**Have the authors made all data and (if applicable) computational code underlying the findings in their manuscript fully available?**

The PLOS Data policy requires authors to make all data and code underlying the findings described in their manuscript fully available without restriction, with rare exception (please refer to the Data Availability Statement in the manuscript PDF file). The data and code should be provided as part of the manuscript or its supporting information, or deposited to a public repository. For example, in addition to summary statistics, the data points behind means, medians and variance measures should be available. If there are restrictions on publicly sharing data or code —e.g. participant privacy or use of data from a third party—those must be specified.

Reviewer #1: Yes

Reviewer #2: Yes

Reviewer #3: Yes

**********

PLOS authors have the option to publish the peer review history of their article (what does this mean?). If published, this will include your full peer review and any attached files.

If you choose “no”, your identity will remain anonymous but your review may still be made public.

**Do you want your identity to be public for this peer review?**  For information about this choice, including consent withdrawal, please see our Privacy Policy.

Reviewer #1: **Yes:** Kazutoyo Miura

Reviewer #2: No

Reviewer #3: No

[NOTE: If reviewer comments were submitted as an attachment file, they will be attached to this email and accessible via the submission site. Please log into your account, locate the manuscript record, and check for the action link "View Attachments". If this link does not appear, there are no attachment files.]

**Figure resubmission:**

-->While revising your submission, we strongly recommend that you use PLOS’s NAAS tool (https://ngplosjournals.pagemajik.ai/artanalysis) to test your figure files. NAAS can convert your figure files to the TIFF file type and meet basic requirements (such as print size, resolution), or provide you with a report on issues that do not meet our requirements and that NAAS cannot fix.

After uploading your figures to PLOS’s NAAS tool - https://ngplosjournals.pagemajik.ai/artanalysis, NAAS will process the files provided and display the results in the "Uploaded Files" section of the page as the processing is complete. If the uploaded figures meet our requirements (or NAAS is able to fix the files to meet our requirements), the figure will be marked as "fixed" above. If NAAS is unable to fix the files, a red "failed" label will appear above. When NAAS has confirmed that the figure files meet our requirements, please download the file via the download option, and include these NAAS processed figure files when submitting your revised manuscript.

**Reproducibility:**

To enhance the reproducibility of your results, we recommend that authors of applicable studies deposit laboratory protocols in protocols.io, where a protocol can be assigned its own identifier (DOI) such that it can be cited independently in the future. Additionally, PLOS ONE offers an option to publish peer-reviewed clinical study protocols. Read more information on sharing protocols at https://plos.org/protocols?utm_medium=editorial-email&utm_source=authorletters&utm_campaign=protocols

# Author comment: Author response to Decision Letter 2

**DOI:** 10.1371/journal.pcbi.1013630.r004

26 Jun 2026

Attachment Submitted filename: Response_to_Reviewers_auresp_2.docx

# Editor report: Decision Letter 2

**DOI:** 10.1371/journal.pcbi.1013630.r005

**Authors:** Necibe Tuncer, Benjamin Althouse

9 Jul 2026

Dear Ms BAUDEMONT,

We are pleased to inform you that your manuscript 'Reconstruction of historical malaria transmission in Senegal using multiplex serocatalytic models.' has been provisionally accepted for publication in PLOS Computational Biology.

Before your manuscript can be formally accepted you will need to complete some formatting changes, which you will receive in a follow up email. A member of our team will be in touch with a set of requests.

Please note that your manuscript will not be scheduled for publication until you have made the required changes, so a swift response is appreciated.

IMPORTANT: The editorial review process is now complete. PLOS will only permit corrections to spelling, formatting or significant scientific errors from this point onwards. Requests for major changes, or any which affect the scientific understanding of your work, will cause delays to the publication date of your manuscript.

Should you, your institution's press office or the journal office choose to press release your paper, you will automatically be opted out of early publication. We ask that you notify us now if you or your institution is planning to press release the article. All press must be co-ordinated with PLOS.

Thank you again for supporting Open Access publishing; we are looking forward to publishing your work in PLOS Computational Biology.

Best regards,

Necibe Tuncer, Ph.D.

Academic Editor

PLOS Computational Biology

Benjamin Althouse

Section Editor

PLOS Computational Biology

***********************************************************

Thank you very much for your revision. The manuscript is nowready for publication.

# Editor report: Acceptance letter

**DOI:** 10.1371/journal.pcbi.1013630.r006

**Authors:** Necibe Tuncer, Benjamin Althouse

PCOMPBIOL-D-25-02114R2

Reconstruction of historical malaria transmission in Senegal using multiplex serocatalytic models.

Dear Dr BAUDEMONT,

I am pleased to inform you that your manuscript has been formally accepted for publication in PLOS Computational Biology. Your manuscript is now with our production department and you will be notified of the publication date in due course.

The corresponding author will soon be receiving a typeset proof for review, to ensure errors have not been introduced during production. Please review the PDF proof of your manuscript carefully, as this is the last chance to correct any errors. Please note that major changes, or those which affect the scientific understanding of the work, will likely cause delays to the publication date of your manuscript.

Soon after your final files are uploaded, unless you have opted out, the early version of your manuscript will be published online. The date of the early version will be your article's publication date. The final article will be published to the same URL, and all versions of the paper will be accessible to readers.

For Research, Software, and Methods articles, you will receive an invoice from PLOS for your publication fee after your manuscript has reached the completed accept phase. If you receive an email requesting payment before acceptance or for any other service, this may be a phishing scheme. Learn how to identify phishing emails and protect your accounts at https://explore.plos.org/phishing.

Thank you again for supporting PLOS Computational Biology and open-access publishing. We are looking forward to publishing your work!

With kind regards,

Sharmila Kamatchi

PLOS Computational Biology | Carlyle House, Carlyle Road, Cambridge CB4 3DN | United Kingdom ploscompbiol@plos.org | Phone +44 (0) 1223-442824 | ploscompbiol.org | @PLOSCompBiol
